# A Functional Approach towards Understanding the Role of the Mitochondrial Respiratory Chain in an Endomycorrhizal Symbiosis

**DOI:** 10.3389/fpls.2017.00417

**Published:** 2017-03-30

**Authors:** Louis Mercy, Eva Lucic-Mercy, Amaia Nogales, Areg Poghosyan, Carolin Schneider, Birgit Arnholdt-Schmitt

**Affiliations:** ^1^INOQ GmbHSchnega, Germany; ^2^ICAAM, University of Évora, ÉvoraPortugal; ^3^Functional Cell Reprogramming and Organism Plasticity (FunCrop), EU Marie Curie Chair, ICAAM, University of ÉvoraÉvora, Portugal; ^4^Functional Genomics and Bioinformatics, Department of Biochemistry and Molecular Biology, Federal University of CearáFortaleza, Brazil; ^5^Science and Technology Park Alentejo (PCTA)Évora, Portugal

**Keywords:** AMF, AOX, COX, phosphorus, mycorrhizal growth dependency, mycorrhizal type, *Rhizoglomus irregulare*, *Solanum tuberosum*

## Abstract

Arbuscular mycorrhizal fungi (AMF) are crucial components of fertile soils, able to provide several ecosystem services for crop production. Current economic, social and legislative contexts should drive the so-called “second green revolution” by better exploiting these beneficial microorganisms. Many challenges still need to be overcome to better understand the mycorrhizal symbiosis, among which (i) the biotrophic nature of AMF, constraining their production, while (ii) phosphate acts as a limiting factor for the optimal mycorrhizal inoculum application and effectiveness. Organism fitness and adaptation to the changing environment can be driven by the modulation of mitochondrial respiratory chain, strongly connected to the phosphorus processing. Nevertheless, the role of the respiratory function in mycorrhiza remains largely unexplored. We hypothesized that the two mitochondrial respiratory chain components, alternative oxidase (AOX) and cytochrome oxidase (COX), are involved in specific mycorrhizal behavior. For this, a complex approach was developed. At the pre-symbiotic phase (axenic conditions), we studied phenotypic responses of *Rhizoglomus irregulare* spores with two AOX and COX inhibitors [respectively, salicylhydroxamic acid (SHAM) and potassium cyanide (KCN)] and two growth regulators (abscisic acid – ABA and gibberellic acid – Ga3). At the symbiotic phase, we analyzed phenotypic and transcriptomic (genes involved in respiration, transport, and fermentation) responses in *Solanum tuberosum/Rhizoglomus irregulare* biosystem (glasshouse conditions): we monitored the effects driven by ABA, and explored the modulations induced by SHAM and KCN under five phosphorus concentrations. KCN and SHAM inhibited *in vitro* spore germination while ABA and Ga3 induced differential spore germination and hyphal patterns. ABA promoted mycorrhizal colonization, strong arbuscule intensity and positive mycorrhizal growth dependency (MGD). In ABA treated plants, *R. irregulare* induced down-regulation of *StAOX* gene isoforms and up-regulation of genes involved in plant COX pathway. In all phosphorus (P) concentrations, blocking AOX or COX induced opposite mycorrhizal patterns *in planta*: KCN induced higher *Arum*-type arbuscule density, positive MGD but lower root colonization compared to SHAM, which favored *Paris*-type formation and negative MGD. Following our results and current state-of-the-art knowledge, we discuss metabolic functions linked to respiration that may occur within mycorrhizal behavior. We highlight potential connections between AOX pathways and fermentation, and we propose new research and mycorrhizal application perspectives.

## Introduction

The political, social and economic context should, in the next years, favor the exploration and exploitation of beneficial soil organisms for crop production. Government policy initiatives (European Directive 2009/128/EC) and consumer demand will lead to alternative production methods in order to reduce the use of phytosanitary products and fertilizer inputs. Thus, agroecological strategies are increasingly explored and recall some basic definitions that (re)integrate the plant in its environment. The soil is the first habitat for plants which continues to interact at all stages of plant’s life cycles (Jeffries et al., 2003). The soil is also one of the main reservoirs of ecosystem services found on Earth, provided by a wide range of microorganisms, including bacteria and some soil fungi, such as arbuscular mycorrhizal fungi (AMF; Gianinazzi et al., 2010). The predominant mutualistic symbiotic relationship between AMF and plant roots was established over 400 million years ago (Brundrett, 2002) in more than 200,000 species, which belong to 74% of plant families (van der Heijden et al., 2015). AMF are obligate biotrophs, represented by at least 289 species^1^ found worldwide under a wide range of ecological conditions. Many positive mycorrhizal effects on host plants have been reported. AMF can (i) improve plant growth by a better transfer of water and inorganic nutrients, especially phosphorus (Smith and Read, 2008); (ii) increase plant-pathogen resistance and plant health (Whipps, 2004; Pozo et al., 2009); (iii) boost plant photosynthesis (Quarles, 1999); (iv) stabilize soil by the excretion of the fungal glycoprotein, glomalin (Rillig and Steinberg, 2002; Bedini et al., 2009); (v) alleviate the impact of abiotic stresses such as cold or heat (Volkmar and Woodbury, 1989; Charest et al., 1993; Zhu et al., 2010, 2011), salinity (Porras-Soriano et al., 2009), drought (Aroca et al., 2007), nutritive starvation (Smith and Read, 2008) or heavy metals (Karimi et al., 2011). In return, it is believed that AMF benefit from the plant’s carbohydrates supply (Bago et al., 2003) associated with the stimulation of fatty acid synthesis in fungal hyphae (Trépanier et al., 2005).

Arbuscular mycorrhizal fungi are probably one of the essential components of the “second green revolution” (Lynch, 2007), but their implementation faces some major difficulties, namely restrictions from plant producer’s perspective, product costs, producer awareness level and variability in mycorrhizal inoculum quality (Vosátka et al., 2008; Ijdo et al., 2011; Berruti et al., 2016). But there are also limitations inherent to the biological system itself since mycorrhizal benefits are not always guaranteed (Vosátka et al., 2008; Ijdo et al., 2011; Malusá et al., 2012) and the physicochemical properties of targeted soils can negatively impact the symbiosis (Smith and Smith, 2011a,b). One of the biggest challenges of mycorrhizal inoculum field application is the high phosphorus (P) content often encountered under conventional cropping, due to P fertilizer input. It is known that high phosphorus concentrations (or its inorganic salt phosphate) systematically inhibit mycorrhizal colonization (Smith and Read, 2008; Breuillin et al., 2010), and the physiological signaling generated by this element appears systemic since foliar application can lead to the same effects (Sanders, 1975; Schreiner and Linderman, 2005; Schreiner, 2010).

Phosphate affects not only the establishment but also the functioning of mycorrhizal symbiosis. Fungal structure development of the internal mycelium can be divided in two general anatomical groups described by Gallaud (1905). The *Arum*-type consists of characteristic highly branched arbuscules within cortical cells, formed from a short side hyphal branch. The *Paris*-type is characterized by the development of extensive intracellular coiled hyphae, which spreads from cell to cell sometimes with only low rates of arbuscule-like branch formation. McArthur and Knowles (1992) have shown in potato root that AMF develop preferentially *Arum*-type under low P while *Paris*-type occurs under high P. These two mycorrhizal types may act in different ways within plant roots. In rice, the symbiotic phosphate transporter (PT) *OsPt11* is preferentially active in arbuscule branches but not around coiled hyphae (Kobae and Hata, 2010). Given that increasing phosphorus concentration is often associated with a decrease of mycorrhizal growth response (MGD; Smith and Smith, 2011a), these data suggest differential plant fitness related to the mycorrhizal type they harbor. Despite numerous studies, there is not yet a complete explanation for the P inhibition. Therefore, there is an urgent need to better understand the physiological bases of this phenomenon in order to define innovative strategies to improve mycorrhizal development and performance, which are *sine qua non* conditions to realize the mycorrhizal implementation under high P crop field conditions.

One obvious connection between P and organism behavior is the mitochondrial respiration activity, in which P plays a crucial role as energetic component of ATP. In most plants and fungi, the respiration yield is modulated by the electron partitioning flow shared between the cytochrome oxidase (COX) and the alternative oxidase (AOX) pathways that take part in the electron transport chain (Vanlerberghe, 2013). Both transfer electrons to O_2_ (which results in water formation), but it is usually assumed that AOX is a non-conserving energy pathway because it does not contribute to ATP formation (Vanlerberghe, 2013) and is regulated by the mitochondrial redox status and the glycolytic flux.

In plants, the COX pathway involves cytochrome *c* reductase, cytochrome *c* and cytochrome *c* oxidase enzymes. Whereas cytochrome *c* (Cytc) is composed of a single small polypeptide, cytochrome *c* oxidase is a multimeric complex composed of several different subunits, encoded by the mitochondrial and the nuclear genome (Welchen et al., 2002). Subunit Vb (*COXVb*) is the most conserved among nuclear-encoded subunits (Rizzuto et al., 1991). *Cytc* is essential for plant growth and survival and the knock-out of both *Cytc* genes in *Arabidopsis* is lethal to the plants while they participate for complex IV stability (Welchen et al., 2012). AOX plays an important role during various stress responses (such as P limitation, Sieger et al., 2005; Plaxton and Tran, 2011) and in specific developmental phases, depending on the expressed isoform (Umbach et al., 2006; Zsigmond et al., 2008; Vanlerberghe, 2013). However, its metabolic significance is much less clear but specific metabolic functions must be involved when the AOX pathway is engaged to sustain basal general metabolic processes associated with the a specific redox status (NAD(P)^+^/NAD(P)H) cell pool in order to cope with energy demand. In this regard, fermentation metabolism activity could play an important role (Sakano, 2001). The best-known function of fermentative metabolism is to recycle NADH to NAD^+^ to avoid the depletion of the cytosolic NAD^+^ pool and inhibition of glycolysis when oxidative phosphorylation is impaired (Sakano, 2001). However, no data is available about the importance of these processes in mycorrhizal symbiosis.

In fungi, AOX plays a role in growth regulation and development, resistance, pathogenesis and pathogenicity, and may contribute to fungal ecological fitness (Umbach and Siedow, 2000; Uribe and Khachatourians, 2008; Ruiz et al., 2011; Grahl et al., 2012; Thomazella et al., 2012; Xu T. et al., 2012). Unlike plants, in which AOX form small multigenic families, the analysis of the fungal genomes currently available reveals that a majority of fungal species possessing the AOX pathway have only one gene sequence, with a maximum of three sequences per genome (Mercy et al., 2015).

In particular, very few studies were conducted to elucidate the role of the two electron pathways in AMF, despite their known importance for the growth of many organisms:

- It was shown that the COX1 protein content is increased in hyphae (Besserer et al., 2006) while the transcript level of *COXIV* is increased in hyphae as compared to spores (Besserer et al., 2008) within days succeeding application of strigolactone analogous (GR24) in *Gigaspora rosea*. Hyphal development seems, therefore, associated with the COX pathway, and it corresponds to a higher NAD(P)H protein activity (concomitant with an increase in NADH dehydrogenase activity) and ATP production observed at hyphal tip (Besserer et al., 2008).

- Existence of the cyanide-insensitive respiration pathway in AMF was highlighted by the presence of an *AOX* sequence in *Rhizoglomus irregulare* genome, close to the *Mucoromycotina AOX 1* (Campos et al., 2015; Mercy et al., 2015), but limited functional data were published. By using SHAM as AOX pathway inhibitor, Besserer et al. (2009) suggested a role of AOX during *Gigaspora rosea* spore germination. Mitochondrial changes (density and respiration) were observed in response to branching factors (Tamasloukht et al., 2003; Besserer et al., 2006), which may suggest a role of the AOX or COX pathways during the pre-symbiotic phase. Campos et al. (2015) observed a coincident of up-regulation of the tomato *AOX1* genes and down-regulation of the *RiAOX* gene during the first six weeks of symbiosis establishment. Expression data obtained under cold-stress conditions showed that the presence of AMF is able to induce an opposite plant mitochondrial respiratory pattern, by potentially reversing the electron route pathway from the AOX to the COX (Liu et al., 2015). Note that the growth regulator abscisic acid (ABA) was shown to play a crucial role in arbuscule formation and functionality (Herrera-Medina et al., 2007; Martin-Rodriguez et al., 2010, 2011; Aroca et al., 2013), while it regulates the AOX gene expression and activity in plants (Finkelstein et al., 1998; Choi et al., 2000; Rook et al., 2006; Giraud et al., 2009; Lynch et al., 2012; Wind et al., 2012). Nevertheless, no roles were clearly defined for the respiratory pathways during spore dormancy or the symbiotic phase.

Although several studies suggest a connection between respiration and P nutrition, little is known about the uptake and transport of P in connection with the respiratory pathways involved. Plant Pi uptake across the plasma membrane is mediated by Pi/H^+^ symporters belonging to the *Pht1* gene family (Bucher, 2007). In mycorrhizal plants, two P uptake pathways were identified: the “direct phosphate uptake” pathway (DPU), mediated by high affinity transporters that are strongly expressed in roots (Smith et al., 2011) and the “mycorrhizal phosphate uptake” pathway (MPU), relying on AM-inducible Pi transporters, crucial for Pi flux across the periarbuscular membrane at the mycorrhizal interface (Javot et al., 2007; Yang et al., 2012). Inorganic phosphate transporters are also present on the inner mitochondrial membrane and are represented by two families: the phosphate/dicarboxylate carrier (DIC) and the phosphate carrier Pht3 (here named ‘MPT’ for mitochondrial phosphate transporter). MPTs deliver most of the Pi required by the mitochondrial ATP synthase complex (Kiiskinen et al., 1997).

This work is an exploration of the functional framework of the cyanide-sensitive and cyanide-insensitive respiration pathways in the mycorrhizal system *Solanum tuberosum*/*Rhizoglomus irregulare* (whose genomes are available). To study this complex aspect in a holobiont system, three strategies were developed: the first assay was implemented to study the impact of respiratory inhibitors SHAM (AOX inhibitor) and KCN (COX inhibitor), as well as, two antagonistic phytohormones (ABA and Ga3) on mycorrhizal spore behavior at the pre-symbiotic phase (axenic condition). The second trial was designed to analyze transcript variations of several genes involved in respiration and fermentation pathways using ABA, a phytohormone known to promote the mycorrhizal symbiosis and also known to be one regulator of the AOX pathway. Then, a third assay consisted to set a non-lethal pharmacological approach using KCN and SHAM treatments, under five different phosphorus concentrations. Our data reveal differential mechanisms that shape plant and fungal behavior by affecting yield, plant FW, mycorrhizal type, hyphal development and MGD. We show that the electron flow partitioning is a key determinant in mycorrhizal behavior and mycorrhizal effects, at least in the *S. tuberosum*/*R. irregulare* biosystem. We discuss its potential relevance in regard to specific metabolic pathways, notably to fermentation, but also for the mycorrhizal application.

## Materials and Methods

### Experiment 1: Spore In vitro Assay

Four viable and mature *in vitro* spores of *R. irregulare* were deposited on four cardinal directions in Petri dishes filled with water + 3.5% Gelrite^TM^ (Duchefa Biochemie, The Netherlands), containing or not ABA (1 mM), Ga3 (1 mM), SHAM (1 or 5 mM) or KCN (1 or 5 mM). Six plates were tested for each treatment and incubated in dark at 28°C in upside down position. At 24 days after inoculation (DAI), hyphal germination patterns were observed and the germination rate was calculated. Spore viability was also assessed by iodonitrotetrazolium salt (INT) and spores, which appeared red due to the presence of formazan, were counted. INT is a marker of mitochondrial activity (Walley and Germida, 1995; Mukerji et al., 2002). It is known that INT reduction (the conversion of INT to formazan by two electrons and two protons) is connected to the electron chain transport (Berridge et al., 2005) but the exact process within cell is not yet clearly identified.

### Experiment 2: Effect of ABA-Driven Pre-trial on Mycorrhizal Performance, Respiration, and Fermentation-Related Genes

The aim of this experiment was to study in a small experimental set the variation of some genes involved in the mitochondrial electron chain and fermentation associated with mycorrhizal performances. *In vitro S. tuberosum* plantlets (cv K19-99-0012) were grown in growth chambers [20°C/17°C (day/night), 16 h d^-1^ photoperiod, 70% relative humidity and 55 μmol m^-2^s^-1^ photon flux density] on MS medium (Murashige and Skoog, 1962), supplemented with 20 g⋅l^-1^ sucrose, 3.5 g l^-1^ Gelrite^TM^ (Duchefa Biochemie, The Netherlands) adjusted to pH 5.6 before autoclaving (121°C for 15 min). Plantlets were pre-treated or not with ABA, a known promoter of the AOX pathway, added in culture medium to 0.1 mM final concentration.

Ten-day-old *in vitro* plantlets were transplanted in 1 L pots containing 100% sterilized sand (baked twice in a dry oven, at 120°C for 6 h). Plants were inoculated (M) or not (NM) at transplanting time with 100 *in vitro* spores of *R. irregulare* INOQ strain QS69 placed at the vicinity of the root and grown in a glasshouse [Loitze, Germany; 32°C/25°C (day/night), natural light and day (July–August)]. In this way, no direct contact between AMF and ABA treatment could occur. Plants were fertilized once a week with 50 ml of a modified Hoagland’s solution without P (1 ppm P was mixed in the sand during pot preparation). The watering was performed when needed with the same volume for all plants. Precautions were taken to avoid watering the shoots. Potato plant and soil harvesting were conducted at 8 weeks after inoculation (WAI). Plant growth parameters such as the shoot, root, and tuber FW were measured. The evaluation of mycorrhizal development was performed according to Trouvelot et al. (1986) method after root staining with China Ink (Vierheilig et al., 1998). The MGD was calculated for several potato parameters (root, shoot, yield and total biomass FW) according to the following formula (Plenchette et al., 1983): [100^∗^((*M* -*NM*)/*M*)], expressed as percentage.

### Experiment 3: Effect of Different Phosphorus Concentrations and Respiratory Inhibitors on Mycorrhizal Performance

A multifactorial experiment was designed to test the effects of different P concentrations and respiratory chain inhibitors on mycorrhizal and plant parameters. Mycorrhizal inoculum was produced in bed cultures with a mix of three plant species (*Trifolium pratense, Zea mays*, and *S. tuberosum*) in sterile sand, containing 92,000 propagules/l [determined by the Most Probable Number of mycorrhizal propagules (MPN) test, Gianinazzi-Pearson et al., 1985]. *In vitro* potato plantlets were grown in growth chambers as described in the Experiment 2 before being transplanted to a glasshouse [Loitze, Germany; 32°C/25°C (day/night), natural light and day (July–August)] and grown for 8 weeks in 1 L pots containing 100% sterilized sand (baked twice in a dry oven, at 120°C for 6 h). Plants were inoculated or not with *R. irregulare* (INOQ strain QS69) at transplanting time, by mixing 4% of a mycorrhizal inoculum containing spores and mycorrhizal root fragments in the growth substrate.

Five P concentrations were tested: 1, 10, 50, 100, and 300 ppm P (respectively, 0.032, 0.323, 1.614, 3.228, and 9.687 mM as final concentration), corresponding to the concentrations of practical reality in crop field soils (50, 100, and 300 ppm) and in mycorrhizal production under glasshouse conditions (1 and 10 ppm). To achieve those concentrations, KH_2_PO_4_ was mixed directly into growth substrate. To test the contribution of each respiratory pathway in mycorrhizal and non-mycorrhizal plants at each P concentration, two respiratory chain inhibitors were used: KCN and SHAM (0.1 mM), which inhibit COX and AOX, respectively. The inhibitors were dissolved in sterile water and added to substrate at 08:00 a.m. in one application 7 DAI, which corresponds to the end of the acclimatization period. The experimental design for the inhibitor studies included (1) non-treated plants, non-inoculated (NM) and inoculated plants (M); (2) KCN treated plants that were non-inoculated (KCN) or inoculated (M KCN) plants; (3) SHAM treated plants that were non-inoculated (SHAM) or inoculated (M SHAM) plants. Plants were fertilized once a week with 50 ml of a modified Hoagland’s solution without P, and were watered as needed with the same volume for all plants. Precautions were taken to avoid watering and treating the shoots.

Potato plant and soil harvesting were conducted at 8 WAI. Plant growth parameters including the shoot, root, and tuber FW, were measured. The evaluation of mycorrhizal development was performed according to the Trouvelot et al. (1986) method after root staining with China Ink (Vierheilig et al., 1998). Parameters investigated (by observing with a microscope 30 root fragments slide per sample) included frequency of mycorrhiza (F %), intensity of mycorrhiza, arbuscule, vesicle and intraradical hyphal colonization in whole root system (respectively, M %, A %, V %, and H %) and within mycorrhizal root fragments (respectively, m %, a %, v %, and h %). The spore production was evaluated in M plants for each repetition by isolating and counting spores after performing the wet sieving method (Gerdemann and Nicolson, 1963) using 3 × 10 g of dried substrate. The MGD was calculated for several potato parameters (root, shoot, yield, and total biomass FW) according to the following formula (Plenchette et al., 1983): [100^∗^((*M* – *NM*)/*M*)], expressed as percentage. The % FW of root, shoot or tuber was calculated as follows: [*Plant organ* (*root, shoot part or tuber FW*)/*Total FW*] × 100.

### Bioinformatic Analyses

Transcript accumulation analyses of plant and fungal genes involved in respiration, nutrient transport and fermentation were performed. Genes of interest are listed in Supplementary Table S1. To obtain the sequences of some targeted genes, a database search was performed using the *R. irregulare* and *S. tuberosum* genomes databases^2,3^ and the INRA *Glomus* database^4^. Full-length amino acid sequences of *R. irregulare, S. tuberosum* and those from fungi and plants were acquired from the JGI database^5^ and GenBank^6^ and were aligned by CLUSTALW and imported into the Molecular Evolutionary Genetics Analysis (MEGA) package version 6 (Tamura et al., 2013). Phylogenetic analyses were conducted using the neighbor-joining (NJ) method implemented in MEGA with the pairwise deletion option for handling alignment gaps and with the Poisson correction model for distance computation. Bootstrap tests were conducted using 1000 replicates.

### Quantitative RT-PCR

For both glasshouse trials, 100 mg of root samples were stored in RNAlater (Qiagen) solution at the time of harvest. Total RNA was extracted from 100 mg of roots conserved in RNAlater (Qiagen) using the RNeasy Plant Mini kit (Qiagen). cDNA synthesis was performed with an oligo(dT) primer (Promega) and reverse transcriptase (Masterscript^TM^ Kit, 5 Prime, Germany) using 250 ng of total RNA. The cDNAs were 1:10 diluted and amplified using the 7500/7500 Fast Real-Time PCR System (Applied Biosystems^®^, USA). Amplification reactions were prepared using a SYBR Green PCR Master kit (Maxima) SYBR Green/ROX qPCR Master Mix (Thermo Scientific) using the following concentrations: 6 μl of PCR water, 9 μL of 2x Maxima SYBR Green/ROX qPCR Master Mix (Thermo Scientific) 0.5 μL of each forward and reverse primer (10 mM) and 2 μl of the cDNA template. Three independent biological replicates were analyzed per treatment and each sample was analyzed in duplicate. The specificity of the different amplicons was checked by a melting curve analysis at the end of the amplification protocol. Several candidates were evaluated for further use as reference gene for normalization of the transcript data of target genes, comprising a set of housekeeping genes, rRNA genes and other sequences (data not shown). After evaluation of expression stability using the applications BestKeeper and NormFinder (Andersen et al., 2004; Pfaffl et al., 2004), two genes for potato (*StEF1α* and *StUbc*, Gallou, 2011, Supplementary Table S1) and one genes for *R*. *irregulare* (*GiICL*, Lammers et al., 2001, Supplementary Table S1) were chosen as reference genes for our experimental conditions. Expression of target genes was evaluated by efficiency corrected relative quantification for *R. irregulare* genes and using the geometric normalization factors for potato where two genes are used for normalization (Pfaffl, 2001; Vandesompele et al., 2002). Standard curves of a fourfold dilution series from pooled cDNAs were used for PCR efficiency calculations. All primers used are listed in Supplementary Table S1.

### Statistical Analyses

In the Experiment 1–3, differences in plant and fungal growth parameters and gene expression between treatments were examined by a one-way analysis of variance (ANOVA), after log or arcsin transformation of values as indicated in figure legends. Dunnett’s test was conducted to identify significant differences (*P* < 0.05, symbolized by stars) compared to a specified standard control and Duncan’s multiple range tests were performed to identify significant differences (*P* < 0.05, symbolized by letters) among P concentrations (after standardization against specified control). Data analysis was performed with the SAS enterprise guide 4.1 (SAS Institute Inc., Cary, NC, USA).

In the Experiment 3, the relationships between P concentration and plant or fungal parameters were investigated by least squares stepwise multiple linear regression with replication using experiment-wise type I error rates of 0.05 for coefficients calculated using the Dunn–Šidák method (Ury, 1976). For each fungal parameter, the complete candidate model included up to the third degree of P concentration or of ln P, three qualitative variables binary coded as 0 or 1 for control, KCN, SHAM plus all first and second level interactions between powers of P and qualitative variables. Whenever considered necessary after graphical exploration of the data, modeling was done with fungal variables or P concentration logarithmically, arcsin transformed or not with the major criteria for model selection being the coefficient of determination (data not shown). Lack of fit was tested for *P* = 0.05 and coefficients of determination (*R*^2^) are presented as proportion of the maximum *R*^2^ possible (Draper and Smith, 1998). Linear regressions and analyses of variances were done with Statgraphics 4.2 (STSC, Inc., Rockville, MD, USA), all other statistics used in regression were performed in Excel^®^ (Microsoft Corporation). Least squares stepwise multiple linear regression data are given in Supplementary Table S2 (under each fungal parameter) and Supplementary Table S3 (under each plant parameter).

## Results

### Experiment 1 – Influences of Respiratory Inhibitors and Two Antagonistic Plant Growth Regulators at the Pre-symbiotic Phase

**FIGURE 1:**
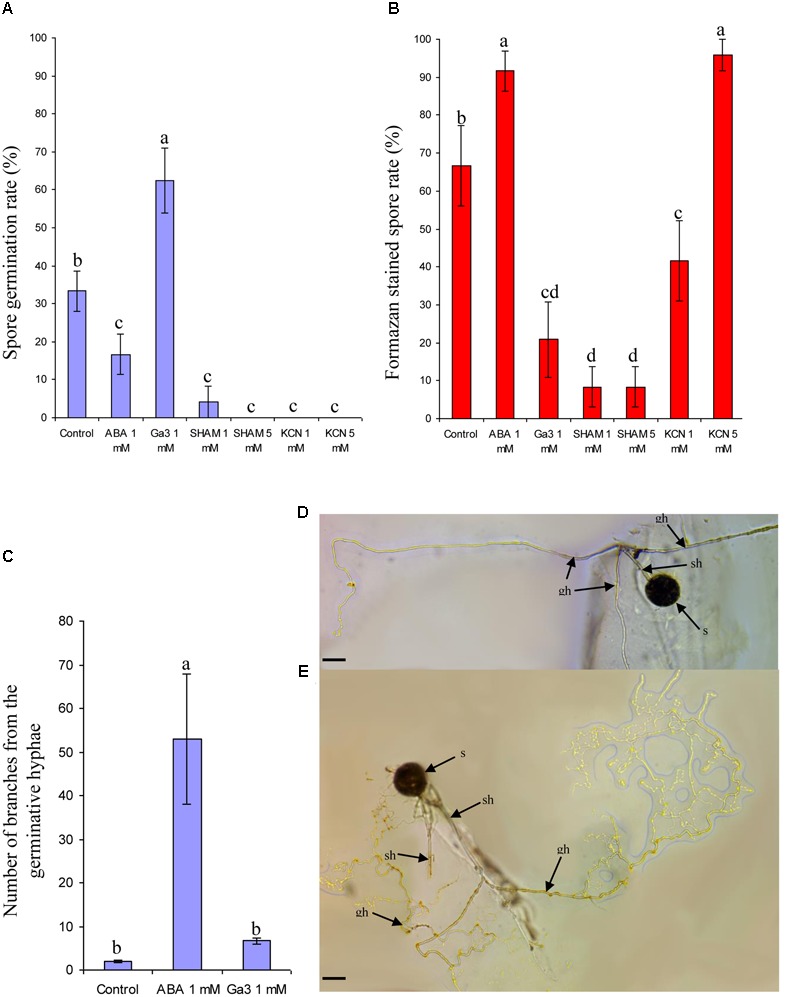
**Effect of two antagonistic plant growth regulators and two respiratory inhibitors on spore germination, spore viability, and germinative hyphal phenotype.** Effect of Ga3 (1 mM), ABA (1 mM), SHAM (1 and 5 mM) and KCN (1 and 5 mM) on spore germination (*R. irregulare*) **(A)** and spore viability **(B)**, number of branches in the germinative hyphae following or not ABA and Ga 3 treatment **(C)** with representative germinative straight hyphal pattern under Ga3 treatment **(D)** or branched hyphal pattern under ABA treatment **(E)**. s, spore; sh, subtending hypha; gh, germinative hypha. Data show *means* (*n* = 24) ± SE Treatments with the same letter are not significantly different (*p* < 0.05 *SNK* multiple-comparison ANOVA), after arcsin transformation of percentage values. Scale bar: 50 μm. Data analysis was performed with the SAS enterprise guide 4.1 (SAS Institute Inc., Cary, NC, USA).

Compared to non-treated spores, ABA treatment decreased the germination rate (**Figure 1A**) but increased significantly the viable spore fraction (containing formazan crystals, **Figure 1B**). Ga3 treatment induced an opposite effect (**Figures 1A,B**). Both SHAM and KCN, at 1 and 5 mM, inhibited spore germination (**Figure 1A**), but induced an opposite reaction on INT reduction ability (**Figure 1B**): red stained spores rate was higher in KCN (for both concentrations) than in SHAM at 1 and 5 mM, and also than in non-treated spores (with 5 mM KCN). Then, ABA induced a significant hyphal branching pattern around the germinated spores (**Figures 1C,E**) compared to spores treated or not with Ga3 (**Figures 1C,D**).

### Gene Sets for Transcriptomic Study in Experiments 2 and 3

The bioinformatic analyses conducted in this study revealed that the *AOX* family of *S. tuberosum* is composed of three *AOX1* and one *AOX2* sequences, called, respectively, *StAOX1a, StAOX1b, StAOX1d* and *StAOX2* according to the AOX classification proposed by Costa et al. (2014). All *StAOX* isoforms were expressed but harbored tissue specificity in our experimental conditions (Supplementary Figure S3). The *R. irregulare* genome contained one single AOX sequence (*RiAOX*).

Two isoforms encoding for cytochrome *c* (*StCytc1* and *StCytc2*) and COXVb (*StCOXIVb1* and *StCOXIVb2*) were found in *S. tuberosum* genome, but *StCytc2* was not expressed in the roots and therefore it was not considered for further analysis (Supplementary Figure S3). The *R. irregulare* genome contained one single gene encoding for cytochrome *c (RiCytc)* and COXVb (*RiCOXIVb*).

The expression of six (*StPT1, 2/6, 3, 4, 5*, and *8*) out of the ten previously described PT genes belonging to the Pht1 family in *S. tuberosum* (Leggewie et al., 1997; Rausch et al., 2001; Nagy et al., 2005; Chen et al., 2014) was studied. The expression of *StPT2* and *StPT6* was not dissociable and was considered as a sum *StPT2/6*. These two genes shared a very high sequence similarity and are tandemly organized on chromosome 3. On chromosome 6, only two complete sequences were found in potato: *StPT7* sequence was incomplete and could not be reconstructed *in silico*. *StPT8, StPT9*, and *StPT10* were all located in the same region of the chromosome 9. The expression of *StPT8*, which shares the highest homology with *SlPT7* (Chen et al., 2014), was studied. In *R. irregulare*, the Pht1 family was divided into four different clusters named after the *S. cerevisiae* sequences therein (Supplementary Figure S4). *ScPHO84*-like cluster groups had putative high affinity P transporters as *ScPHO84* and contained four putative *R. irregulare* sequences previously described (*RiPT1, RiPT2, RiPT3*, and *RiPT4*; Fiorilli et al., 2013; Walder et al., 2016). We found complete and functional sequences only for *RiPT1* and *RiPT3*. Within the putative low affinity P transporters, grouping with the yeast transporters *ScPHO87* and *ScPHO90* described by Pinson et al. (2004), only one *R. irregulare* gene was found (*RiPT7*). The third cluster groups sequences presented homology with the *S. cerevisiae* high affinity Na^+^/Pi cotransporter *ScPHO89* (Sengottaiyan et al., 2013) and two *R. irregulare* sequences (*RiPT5* and *RiPT6*). *RiPT6* presented an incomplete sequence and no expression in our experimental conditions. The last cluster contained *ScPHO88*-like sequences, which were much shorter (about 189 aa) and did not present a transport activity. One *R. irregulare* gene matched with this sequence (*RiPT8*), but as it did not encode a functional transporter, the transcription of this gene was not monitored in this study.

Concerning mitochondrial P transport, four gene sequences encoding a transporter were found in *S. tuberosum* genome, and one *R. irregulare* in genome (Supplementary Figure S5). In potato, three of the four genes were expressed in roots (*StMPT1a, StMPT1b*, and *StMPT3*). *StMPT2* was found to be expressed only in fruits in our experimental conditions (Supplementary Figure S3).

Then, two *ldh* and three *pdc* sequences were found in potato genome (Supplementary Figures S6 and S7). It was noted that transcript level was higher for *StLDH* expression than for *StPDC* in root, comparing relative values. One *ldh* sequence was found in the *R. irregulare* genome (*RiLDH*).

### Experiment 2: Influence of ABA on Mycorrhizal Behavior and Expression Pattern of Genes Involved in Mitochondrial Electron Chain

**FIGURE 2:**
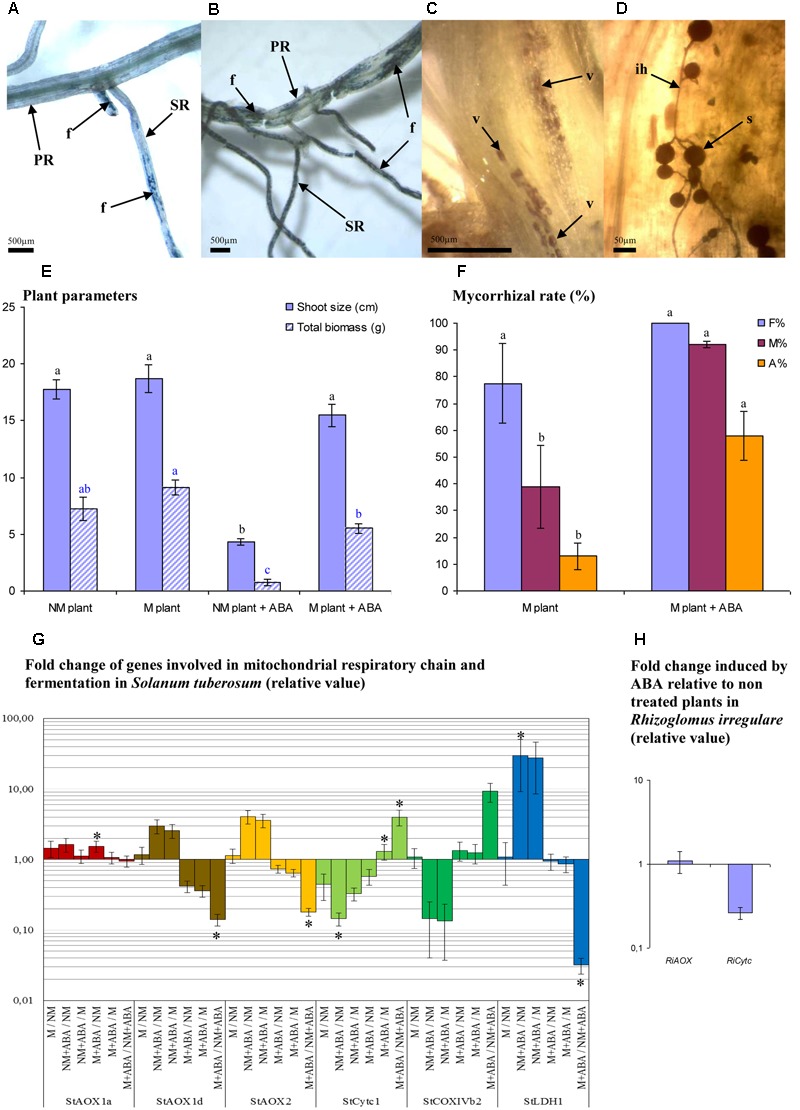
**Influence of ABA on mycorrhizal development and gene expression involved in electron partitioning.** Mycorrhizal development (*R. irregulare*) observed at 8 WAI in potato roots (cv. KK19–0012) pre–treated or not with ABA (0.1 mM) during the *in vitro* phase culture. **(A)** typical fungal development in plants non-treated with ABA **(B)** typical fungal development in plants pretreated with ABA; **(C,D)** mycorrhizal development on a stem of ABA pretreated plant; **(E)** growth parameters of potato plant inoculated or not with *R. irregulare* after ABA pretreatment or not (8 WAI); **(F)** mycorrhizal rate between inoculated plant treated (M plant + ABA) or not pretreated (M plant) with ABA; **(G,H)**: expression pattern of genes involved in electron partitioning. PR, primary adventitious root; SR, secondary adventitious root; f, mycorrhizal structure; v, vesicle; s, spore; ih, intraradical hypha; F%, frequency of mycorrhizal development in root system; M%, intensity of mycorrhizal development in the root; A%, intensity of arbuscule development in whole root system. For plant and fungal parameters, treatments with the same letter are not significantly different (*P* < 0.05 *SNK* multiple-comparison ANOVA, *n* = 4). Statistical tests were performed separately for each plant parameters and each fungal parameters, after arcsin transformation for percentage values. For expression data **(G,H)**, significant difference are indicated by stars above graphs (^∗^*P* < 0.05). Data analysis was performed with the SAS enterprise guide 4.1 (SAS Institute Inc., Cary, NC, USA).

Following ABA pretreatment, mycorrhizal structures were observed in the primary adventitious root and even in the stem base (**Figures 2A–D**), which seems to be a rare case in potato roots under normal conditions (Mercy – non-published observation). AMF increased plant total FW biomass in non-pretreated plants by 1.26-fold (MGD = 20.6%) and in ABA pretreated plants by 7.33 (MGD = 86.4%). Pretreatment with ABA during the *in vitro* phase induced a long-term effect, since a strong plant growth depression was observed (in NM plants, **Figure 2E**). Mycorrhizal colonization and arbuscule intensity were significantly promoted within ABA pretreated plants (**Figure 2F**) compared to non-pretreated plants, consistent with observations performed on tomato plants (Herrera-Medina et al., 2007).

The expression analyses of genes involved in the respiratory chain revealed that *StAOX1d* and *StAOX2* were significantly down-regulated while *StCytc1* was significantly up-regulated in M versus NM plants in presence of ABA (**Figure 2G**). The expression of these genes remained unaffected by the presence of AMF and in the absence of treatment. Comparing to NM and M non-treated plants, ABA treatment induced similar trends of up-regulation for *StAOX1d* and *StAOX2* (although changes are not significant), while *StCytc1* was down-regulated (significant when comparing NM plants). Contrasting with this observation, the presence of AMF in ABA plants induced significant down-regulation for *StAOX1d* and *StAOX2* and up-regulation for *StCytc1* (significant) and *CoxIVb2* (tendency) when compared to non-inoculated ABA plants. It was observed a strong up regulation of *StLDH1* induced by ABA compared to non-treated plants, inoculated (tendency) or not (significant). This gene was significantly down-regulated in presence of AMF (within ABA plants group). Regarding *RiAOX* and *RiCytc* (**Figure 2H**), no significant effect induced by ABA was observed, although *RiCytc* tended to be down-regulated.

### Experiment 3

#### The Fungal Colonization Is Influenced by Phosphorus Level and Respiratory Inhibitors

**FIGURE 3:**
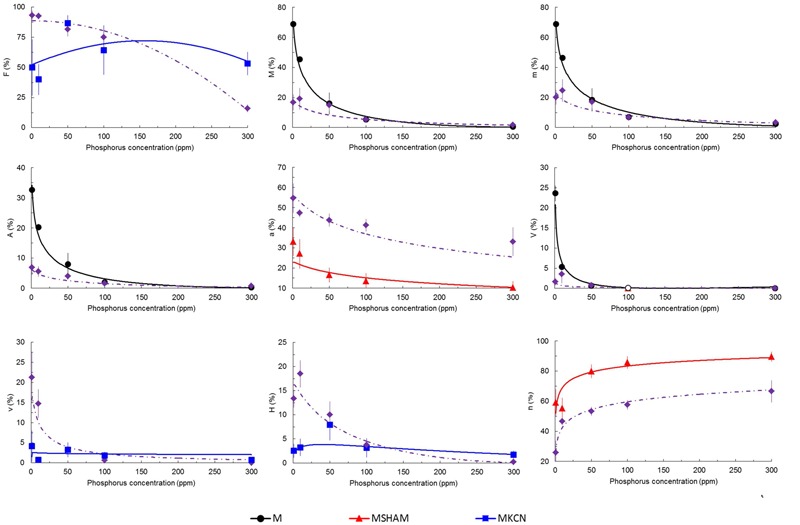
**Predicted and observed (means ± SE) mycorrhizal rate parameters of potato plants treated with respiratory chain inhibitors or not, inoculated with *R. irregulare* under five phosphorus concentrations.** Parameters estimated according to Trouvelot et al. (1986) in potato roots inoculated with *R. irregulare*, harvested at 8 WAI. Plantlets not treated (M, black continuous lines and closed circles), treated by SHAM (at 7 DAI – 0.1 mM; red continuous lines and closed triangles) or by KCN (at 7 DAI – 0.1 mM; blue continuous lines and closed squares) through five phosphorus concentrations (harvesting at 8 WAI, *n* = 3). Whenever treatments did not differ significantly they are represented together as one line by purple dots and dashed lines and closed diamonds. F%, frequency of mycorrhizal development in root system; M%, intensity of the mycorrhizal development in roots; m%, intensity of mycorrhizal development in mycorrhizal root fragments; A%, intensity of arbuscules in whole root system; a%, intensity of arbuscules in mycorrhizal root fragments; V%, intensity of vesicles in whole root system; v%, intensity of vesicles in mycorrhizal root fragments; H%, intensity of hyphal development in whole root system; h%, intensity of hyphal development in mycorrhizal root fragments. Linear regressions and analyses of variances were done with Statgraphics 4.2 (STSC, Inc., Rockville, MD, USA), all other statistics used in regression were performed in Excel^®^ (Microsoft Corporation). Regression coefficients after solving the overall equation fitted are shown in the Supplementary Table S2.

Fungal phenotypic data are indicated in **Figure 3** and Supplementary Figure S1. The mycorrhizal colonization decreased with increasing P concentrations in all treatments. Many fungal structures, such as hyphae, arbuscules, vesicles, and frequency of mycorrhiza, were affected by KCN and SHAM, especially under low P concentration (1 and 10 ppm, **Figure 3** and Supplementary Figure S1). Compared to non-treated mycorrhizal plants (M plants), SHAM treatment significantly reduced the arbuscule (A %) formation to the benefit of an enhanced intraradical hyphal development (h %, **Figure 3**). KCN treatment strongly inhibited the fungal development at 1 and 10 ppm P (Supplementary Figure S1), but a significant higher a % was observed (**Figure 3**) compared to SHAM among P concentrations. At 300 ppm P, highest values were observed for almost all fungal parameters under KCN treatment (Supplementary Figure S1). Hyphal development (H %) in M KCN was reduced compared to M and M SHAM plants at 1 and 10 ppm P (**Figure 3**).

#### The Mycorrhizal Type Is Influenced by Phosphorus Level and Respiratory Inhibitors

**FIGURE 4:**
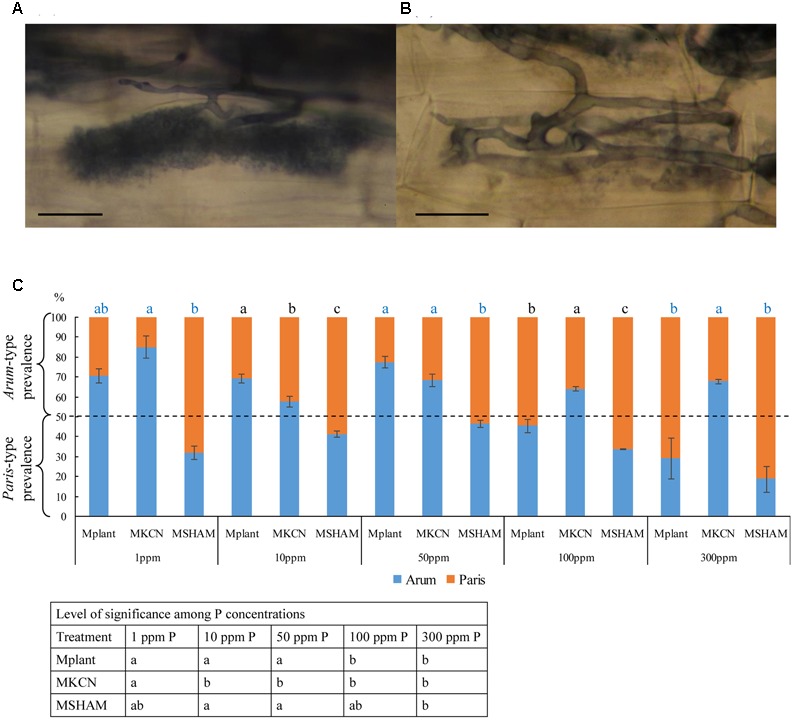
**Mycorrhizal type’s prevalence within potato roots, following treatments or not with respiratory chain inhibitors, under five phosphorus concentrations.** Mycorrhizal types within potato roots: **(A)** *Arum*-type, **(B)** *Paris*-type (staining: China Ink); **(C)** percentages of mycorrhizal type distribution under five different phosphorus concentrations in non-treated control plants and plants treated by respiratory inhibitors. Scale bar: 10 μm. Data show means (*n* = 3) ± SE. Treatments with the same letter are not significantly different (*P* < 0.05, Duncan’s multiple range tests ANOVA). Statistical tests were performed after arcsin transformation of percentage values. The level of significance among treatment per P concentration is given above the graphs, and the one among P concentrations for a given treatment is indicated in the table below graph. Data analysis was performed with the SAS enterprise guide 4.1 (SAS Institute Inc., Cary, NC, USA).

Both *Arum*-type and *Paris*-type structures were observed in roots (**Figures 4A,B**, respectively) across the different treatments, and their occurrences were influenced by both P concentrations and respiratory chain inhibitors (**Figure 4C**). In M plants, *Arum*-type predominated under low P concentrations (1 to 50 ppm) while *Paris*-type predominated at high P concentration (300 ppm). The application of both respiratory inhibitors suppressed this P influence. SHAM treatment induced a disorganization of arbuscule branching similar to the effect of a high P concentration (300 ppm P) in non-treated plants and *Paris*-type hyphal development was predominant for all P concentrations. Under KCN treatment, *Arum*-type was predominant for all P concentrations. The occurrence of *Arum*-type in KCN was significant compared to *Paris*-type in SHAM for all P concentrations.

#### The Spore Production Is not Linked to either with P Concentrations, nor with Mycorrhizal Rate

**FIGURE 5:**
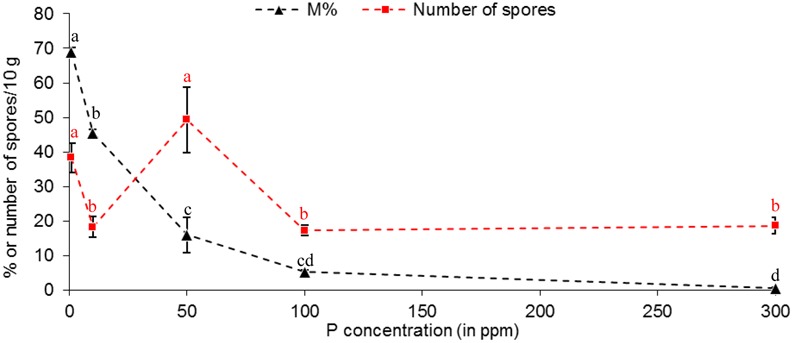
**Effect of phosphorus concentrations in non-treated plants on mycorrhizal sporulation, compared to mycorrhizal development in root.** *R. irregulare* spore number in 10 g of substrate and intensity of mycorrhiza in root (M%, added only to visualize the differences with sporulation profile) at 8WAI through phosphorus concentration after inoculation of potato *in vitro* plantlets. Data show means (*n* = 3) ± SE. Treatments with the same letter are not significantly different (*P* < 0.05, Duncan’s multiple range tests ANOVA). Statistical tests were performed separately for each fungal parameter, and after arcsin transformation of percentage values (M %). Data analysis was performed with the SAS enterprise guide 4.1 (SAS Institute Inc., Cary, NC, USA).

A maximum number of spores was observed at 50 ppm P (**Figure 5**), and then in a lesser extent, at 1 ppm. The number of spores produced at 10, 100 and 300 ppm P was similar, and significantly lower than at 1 and 50 ppm P. No correlation between spore production and P concentration was observed (*r*^2^ = 0.1834). No correlation between spore production and any mycorrhizal parameters was observed among P concentrations, which might indicate the involvement of at least two different metabolic determinants. We noticed that highest values for spore number, a % and *Arum*-type were obtained at 1 and 50 ppm P.

#### Respiratory Chain Inhibitors Reveal Opposite Plant Performance between Inoculated and Non-inoculated Plants

**FIGURE 6:**
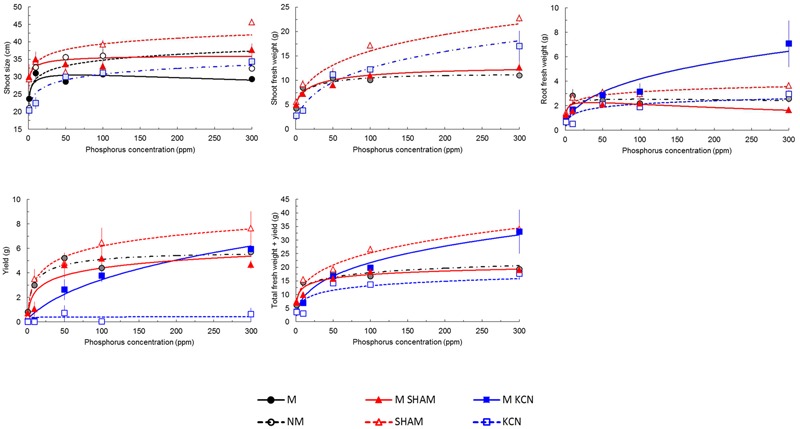
**Predicted and observed (means ± SE) of potato *in vitro* plantlets growth parameters treated with respiratory chain inhibitors or not and inoculated or not with *R. irregulare* under five phosphorus concentrations.** Plantlets were inoculated (M, black continuous lines and closed circles) or not (NM, black dashed lines and open circles) with *R. irregulare*, treated by SHAM (at 7 DAI – 0.1 mM) and inoculated (M SHAM, red continuous lines and closed triangles) or not (SHAM, red dashed lines and open triangles), and treated by KCN (at 7 DAI – 0.1 mM) and inoculated (M KCN, blue continuous lines and closed squares) or not (KCN, blue dashed lines and open squares) through five phosphorus concentrations (harvesting at 8 WAI, *n* = 3). Whenever M and NM treatments did not differ significantly for a given treatment same colors and symbols were used except that dots and dashed lines and half tone symbols were used. Linear regressions and analyses of variances were done with Statgraphics 4.2 (STSC, Inc., Rockville, MD, USA), all other statistics used in regression were performed in Excel^®^ (Microsoft Corporation). Regression coefficients after solving the overall equation fitted are shown in Supplementary Table S3.

Plant phenotypic data are indicated in **Figure 6** and Supplementary Figure S2. Presence of AMF in non-treated plants induced few specific responses to P concentrations on plant vegetative parameters including shoot and root biomass, yield and total biomass. Only shoot size was significantly promoted among P concentrations (**Figure 6**).

Under KCN treatment, the presence of AMF significantly improved all vegetative plant parameters under all P concentrations, whereas under SHAM treatment, root biomass, yield and total biomass were decreased (**Figure 6**). Thus, KCN and SHAM treatments had opposite effects when comparing inoculated and non-inoculated plants for yield and FW total biomass. M KCN plants showed the highest root FW values at 300 ppm P with an increase of 2.34- and 4.24-fold, respectively, compared to M and M SHAM plants (Supplementary Figure S2).

**FIGURE 7:**
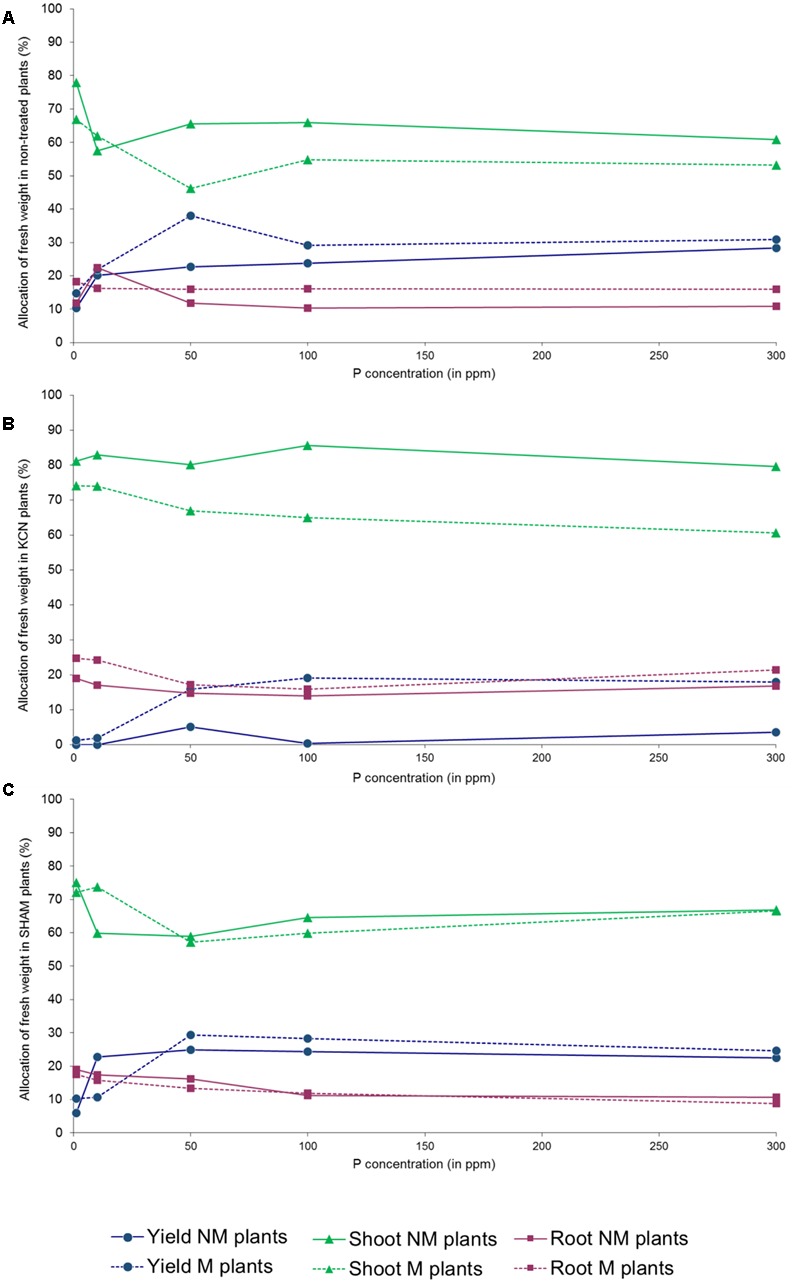
**Percentage of FW attributed to the shoot, root and tuber in potato *in vitro* plantlets.** Plants are inoculated with *R. irregulare* **(A)** without treatment; **(B)** treated by KCN (at 7 DAI – 0.1 mM); **(C)** treated by SHAM (at 7 DAI – 0.1 mM) through five phosphorus concentrations (data obtained after 8 WAI).

We noticed that the tuber FW yield was not proportional to P concentrations in several treatments (Supplementary Figure S2): a peak was observed in NM, M and KCN plants at 50 ppm P, while maximum values were obtained at 100 ppm P in M SHAM plants. Similarly, the total biomass FW increase among P concentration was not proportional in NM plants where a peak was observed at 50 ppm P, but not in M plants. FW biomass data revealed that AMF had an impact on plant FW biomass partitioning (**Figures 7A–C**). The percentage of FW attributed to the root part was relatively stable for a given treatment among P concentrations, with the exception of NM plants (**Figure 7A**) at 10 ppm (higher value). When non-treated plants were inoculated with AMF, the profile of the FW percentage attributed to the shoot was opposite to the one attributed to the tuber yield and an anomaly was observed at 50 ppm P. In all treatments and P concentrations, except SHAM plants at 10 ppm P, the presence of AMF enhanced FW biomass allocation to the yield, to the detriment of the shoot.

#### Impact of Respiratory Inhibitors on the Mycorrhizal Yield Dependency

**FIGURE 8:**
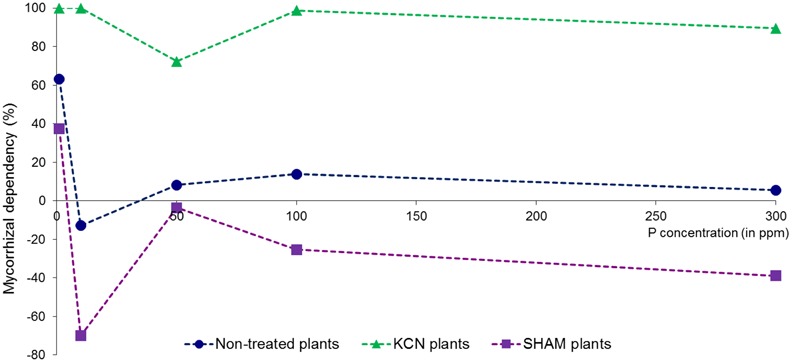
**Mycorrhizal growth dependency (yield) in potato *in vitro* plantlets.** Plants are inoculated with *R. irregular*e without treatment or treated by SHAM (at 7 DAI – 0.1 mM) or treated by KCN (at 7 DAI – 0.1 mM) through five phosphorus concentrations (data obtained after 8 WAI).

The mycorrhizal dependency was affected by P in M plants without any direct correlation with the concentration (**Figure 8**), as the maximum value was observed at 1 ppm P (63.05%) and the lowest at 10 ppm P (-12.48%). The higher P concentration yielded low but positive values. The mycorrhizal dependency presented an opposite pattern between KCN and SHAM treatments. The yield formed under KCN conditions was almost totally dependent (close to 100%) of the presence of AMF, and the lowest value was observed at 50 ppm. In contrast, with the exception of 1 ppm P, a negative mycorrhizal yield response was observed in SHAM treatment in all P concentrations tested.

#### Modulation of Genes Involved in Mitochondrial Electron Chain and Fermentation by Phosphorus and Respiratory Inhibitors

**FIGURE 9:**
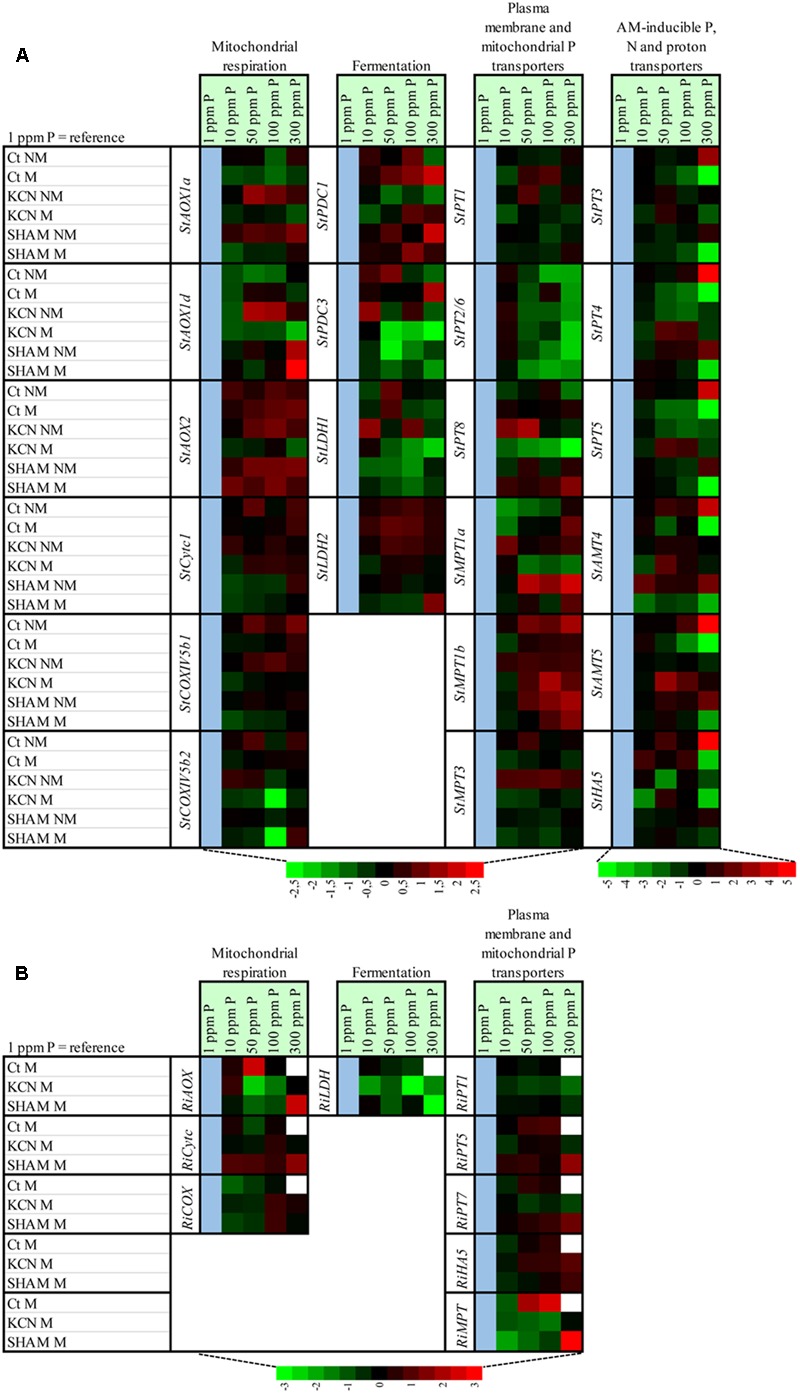
**Heat map showing the effect of P concentration on the relative expression of genes involved in mitochondrial respiratory chain, fermentation, phosphorus, nitrogen and proton plasma membrane transporters and mitochondrial phosphorus transporters in potato root and in R. *irregulare*, following inoculation or not of AMF, treatment or not with two respiratory inhibitors through two phosphorus concentrations.** The heat map shows the real-time quantitative RT-PCR (qRT-PCR) analysis results of genes involved in mitochondrial respiratory chain (three *StAOX* isoforms, *StCytc1, StCOXVb1, StCOXVb2, RiAOX, RiCytc*, and *RiCOXIVb*), fermentation (two *StPDC* and two *StLDH* isoforms and *RiLDH*), phosphorus, nitrogen and proton plasma membrane transporters (six *StPT* isoforms, two *StAMT* isoforms, *StHA1*, three *RiPT* isoforms and *RiHA5*) and mitochondrial phosphorus transporters (three *StMPT* isoforms and *RiMPT*) in potato root **(A)** and in *R. irregulare* **(B)**, under five phosphorus concentrations and following treatment by KCN (at 7 DAI – 0.1 mM) or SHAM (at 7 DAI – 0.1 mM) or not treated (*C*t). Plants were inoculated (M) or not (NM) by *R. irregulare*. The expression levels of genes are presented using fold-change values transformed to Log2 format compared to 1 ppm P as reference (cell in blue). White cells correspond to non-determined analyses. The Log2 (fold-change values) and the color scale are shown at the bottom of heat map.

Variation of the expression of genes involved in the respiratory chain and fermentation among P concentrations (1 ppm used as reference) for each treatment, in potato and *R. irregulare*, are indicated in **Figure 9** and Supplementary Figures S8, S9.

At plant side, data showed that the regulation pattern from genes encoding for AOX, COX, or fermentation pathways were mostly specific to the P concentration (such as a down-regulation of *StAOX1a* at 100 ppm in NM plants, an up-regulation of *StAOX1d* in NM KCN plants at 50 ppm or a down-regulation of *StCOXIVb2* in M SHAM plants at 100 ppm). We noticed that a significant common up-regulation was observed at 50 ppm P for genes involved in cytochrome pathway (*StCytc1, StCOXVb1-2*) in non-inoculated plants but not in inoculated plants (non-treated group). Specific responses to P concentration were also noted for genes involved in fermentation. We observed that *StPDC1* in M plants was more expressed and *StLDH1* was more repressed in M KCN with increasing P concentrations.

Within *R. irregulare*, some transcript variations were observed at specific P concentrations but the tested genes were mostly unaffected. In M plants, when compared to 1 ppm P, a significant up-regulation was observed at 50 ppm for *RiAOX*, while *RiCytc* harboured an opposite profile pattern (although not significant). These data were concomitant with the highest sporulation (**Figure 5**). In contrast with non-treated conditions, a significant down-regulation was observed at 50 ppm for *RiAOX* in M KCN plants. It is noteworthy that no detection was obtained at 300 ppm P from M plants.

**FIGURE 10:**
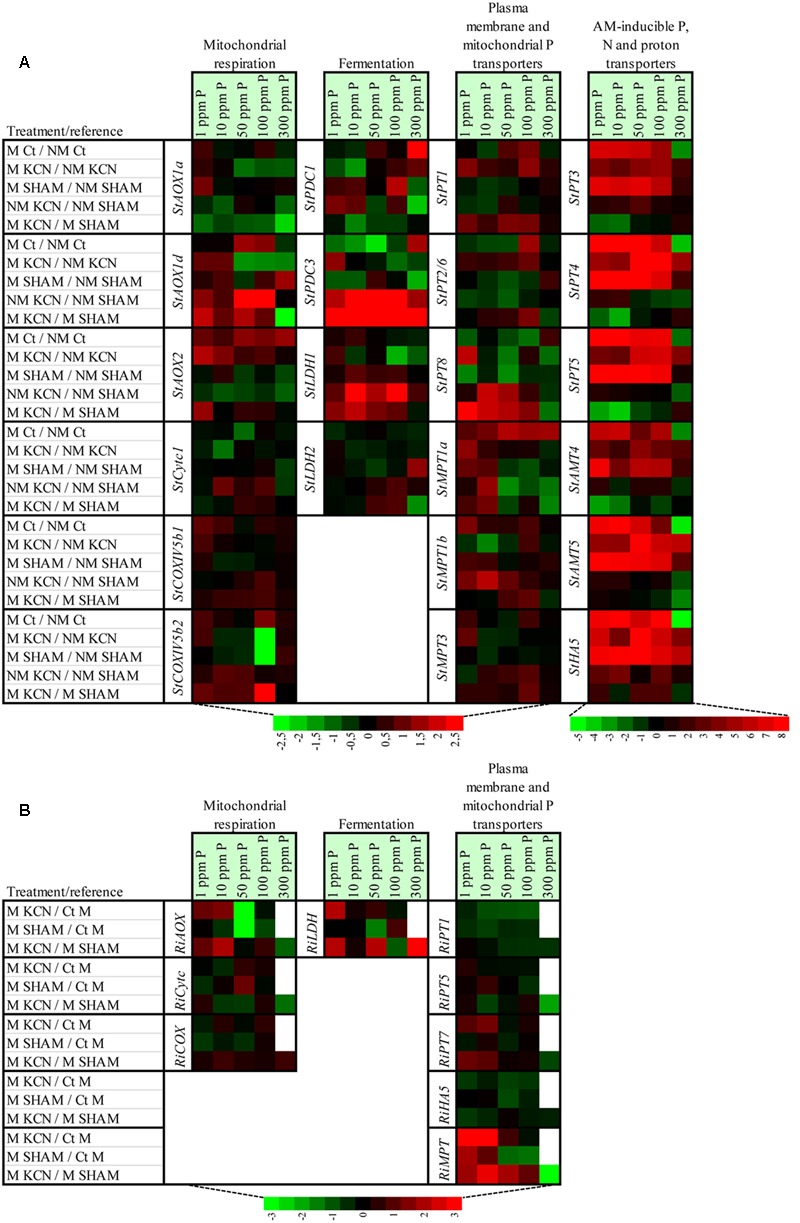
**Heat map showing the effect of treatments on the relative expression of genes involved in mitochondrial respiratory chain, fermentation, phosphorus, nitrogen and proton plasma membrane transporters and mitochondrial phosphorus transporters in potato root and in R. *irregulare*, following inoculation or not of AMF, treatment or not with two respiratory inhibitors through five phosphorus concentrations.** The heat map shows the real-time quantitative RT-PCR (qRT-PCR) analysis results of genes involved in mitochondrial respiratory chain (three *StAOX* isoforms, *StCytc1, StCOXVb1, StCOXVb2, RiAOX, RiCytc* and *RiCOXIVb*), fermentation (two *StPDC* and two *StLDH* isoforms and *RiLDH*), phosphorus, nitrogen and proton plasma membrane transporters (six *StPT* isoforms, two *StAMT* isoforms, *StHA1*, three *RiPT* isoforms and *RiHA5*) and mitochondrial phosphorus transporters (three *StMPT* isoforms and *RiMPT*) in potato root **(A)** and in *R. irregulare* **(B)**, under five phosphorus concentrations and following treatment by KCN (at 7 DAI – 0.1 mM) or SHAM (at 7 DAI – 0.1 mM), or not treated (*C*t). Plants were inoculated (M) or not (NM) by *R. irregulare*. The expression levels of genes are presented using fold-change values transformed to Log2 format compared to mentioned treatment in the table. White cells correspond to non-determined analyses. The Log2 (fold-change values) and the color scale are shown at the bottom of heat map.

Variation of the expression of genes involved in the respiratory chain and fermentation between treatments (non-inoculated plants used as reference) for each P concentration, in potato and *R. irregulare*, are indicated in **Figure 10** and Supplementary Figures S10, S11.

In potato, transcript responses for genes encoding for enzymes involved in the AOX and COX pathways were mostly specific to P concentration. In particular, the presence of AMF compared to their absence (non-treated plants) at 50 ppm P induced an up-regulation of *StAOX2* but a down-regulation of *StCytc1*. Effect of treatments on the expression of genes involved in fermentation pathway were also specific to P concentration. Significant up-regulation was observed for *StPDC1* and *StLDH1* when comparing KCN to SHAM treatments (in plants inoculated or not), but with some specificities regarding P concentrations.

In the fungus, expression levels of *RiAOX, RiCytc*, or *RiCOXIVb* appeared mostly constitutive when comparing KCN to SHAM treatments. In contrast, KCN induced significant strong up-regulation of *RiLDH* at 1, 50, and 300 ppm P compared to SHAM.

To summarize, the inoculation or the use of respiratory inhibitors induced specific expression patterns for genes encoding for enzymes involved in the AOX and COX pathways, but *StAOX* isoforms were unexpectedly not highly induced by KCN compared to SHAM treatments. No obvious interpretation can be easily deduced from these data, underlining complex regulations that were contrasted with those obtained in the Experiment 2. We noticed, nevertheless, that KCN seemed to induce a higher fermentation context compared to SHAM, within potato (inoculated or not) and also in *R. irregulare*.

Several correlations were found between expression of genes and fungal parameters in non-treated plants (Supplementary Table S4) or treated with KCN (Supplementary Table S5) or with SHAM (Supplementary Table S6). Significant negative correlations were observed between M %, m %, A %, and V % and *StAOX2*, but a positive correlation with the H % parameter (non-treated plants) was found. Significant negative correlations were also noticed between *StCytc1* and F % and a %, between *StCOXVb1* and F % and between *StCOXVb2* and h %. Under KCN, positive correlations were observed between *StAOX1a* and A % and V % and also between *StAOX1d* and A % but a negative correlation between *StAOX1d* and H % was detected. Under SHAM, a significant negative correlation was found between F % and *StAOX1d*. No correlation was found between tested genes involved in AOX or COX pathway with mycorrhizal responses on plant phenotype in any treatment.

#### Modulation of Genes Involved in Nutrient and Proton Transport by Phosphorus and Respiratory Inhibitors

Variation of the expression of genes involved in nutrient and proton transport among P concentrations (1 ppm used as reference) for each treatment, in potato and *R. irregulare*, are indicated in **Figure 9** and Supplementary Figures S8, S9.

In potato, a common expression pattern was observed at 300 ppm P for *StPT3, StPT4, StPT5, StAMT4, StAMT5*, and *StHA5*, which were up-regulated in NM plants but repressed in M plants. This response was much less pronounced in plants inoculated and treated with SHAM and disappeared in the other treatments (NM KCN, M KCN, and NM SHAM). *StPT1* remained mostly unaffected by inoculation or treatment, *StPT2/6* was down-regulated by high P in M plants treated or not with respiratory inhibitors, but also in NM SHAM plants. Some specific responses were obtained depending on P concentration and treatment for *StPT8*.

Concerning mitochondrial P transport, *StMPT1a* expression in NM plants tended to increase with P concentration, from 10 to 300 ppm P, although no significant variations were observed when comparing the various P concentrations to 1 ppm P. SHAM treatment tended to up-regulate this gene at high P concentrations (50 to 300 ppm). *StMPT1b* was up-regulated by high P in plants non-treated (non-inoculated) or treated by SHAM (inoculated or not).

Variation of the expression of genes involved in nutrient and proton transport between treatments (non-inoculated plants used as reference) for each P concentration, in potato and *R. irregulare*, are indicated in **Figure 10** and Supplementary Figures S10, S11.

At plant side, as common response, the presence of AMF, in plants treated or not with respiratory inhibitors, up-regulated the expression of *StPT3, StPT4, StPT5, StAMT4, StAMT5*, and *StHA5* for all P concentrations, except at 300 ppm within non-treated plants. Such strong responses were not found for *StPT1, StPT2/6*, and *StPT8* in any treatment. In KCN plants, presence of AMF was associated with up-regulation of *StPT1* at 1, 50, and 100 ppm P and *StPT2/6* at 100 ppm P, compared to plants treated or not with SHAM. Significant strong correlations were found between AM-inducible P transporters and M % or A % (in plants treated or not with SHAM, Supplementary Tables S4, S6) but most of these correlations were not observed in plants treated with KCN (Supplementary Table S5). Tested genes involved in mitochondrial transport were up-regulated in presence of AMF in non-treated plants in all P concentrations, but responses were specific to the isoform. In the presence of respiratory inhibitors, their expression patterns were more complex with specific responses regarding P concentration.

The expressions of *RiPT1, RiPT7*, and *RiHA5* were monitored in this study and highlighted that there were no transcript level changes across all treatments and P concentrations (**Figure 9**). Only *RiPT5* was induced at high P conditions with an up-regulation at 50 and 100 ppm P in control conditions. Regarding *RiMPT*, when compared to 1 ppm P, a significant up-regulation was observed at 300 ppm P (**Figure 9**) in plants treated with SHAM, and non-treated plants at 100 ppm P, and a significant down-regulation was observed at 300 ppm when comparing KCN to SHAM (**Figure 10**).

## Discussion

### Mitochondrial Respiratory Inhibitors Influence Fungal Behavior at Pre-symbiotic Phase

Dormancy is often related to AOX pathway in plant seeds, but also in fungi. Several examples have been reported, especially within *Mucoromycotina* (Cano-Canchola et al., 1988; Salcedo-Hernandez et al., 1994) which are phylogenetically closely related to AMF (Hibbett et al., 2007), showing a respiratory shift characterizing spore germination process from AOX (dormancy) to COX (hyphal growth). It is difficult to define AOX/COX interplay during spore germination in *R. irregulare* as spores can return into dormancy and germinate again several times without obvious phenotypic signs (Giovanetti et al., 2010), and the hyphal tube germination is the physiological consequence of an already implemented respiratory shift, as shown for *Mucor rouxii* (Cano-Canchola et al., 1988).

Nevertheless, our data (Experiment 1) with KCN suggest that AOX is very likely involved in spore dormancy. Application of SHAM or KCN inhibited the spore germination, supporting the importance of both electron pathways and a possible involvement of a respiratory shift from AOX to COX. However, further work is needed to confirm this statement. AMF spores are sensitive to plant hormones, and their germination responses harbor opposite patterns between the antagonistic hormones ABA and Ga3, as ABA maintained spore dormancy while Ga3 broke it, similarly to plant seeds (White and Rivin, 2000; Linkies and Leubner-Metzger, 2012). Therefore, these observations support the need to apply ABA as pre-treatment (Experiment 2) and the need to apply the respiratory inhibitors some days after plant inoculation (Experiment 3) in order to not disturb the pre-symbiotic developmental phase of *R. irregulare*. Promotion of hyphal branching pattern in ABA treatment fits with previous observations: Juge et al. (2002, 2009) showed that spores develop g-type pattern germination (fine branching hyphae) around spores when dormancy is incompletely broken or under stress conditions, while G-type pattern (runner hyphal growth pattern) occurs in favorable conditions. Hyphal branching from germ tube generated by ABA seems similar to its effect on the arbuscule formation (Herrera-Medina et al., 2007).

As a remark, INT staining data suggested a low spore viability in Ga3 and SHAM treatment and higher after ABA and KCN (5 mM) treatment. However, no formazan production was observed in germinated spores following Ga3 application, while the germinative hypha was still growing (data not shown). This suggests that INT staining could correspond to a reducing power marker in the cell, and might be associated to AOX metabolism and spore dormancy, rather than solely to a vital staining.

### Mitochondrial Respiratory Inhibitors Influence Fungal Behavior at Symbiotic Phase

Occurrence of the *Arum*- and *Paris*-types are not well understood and corresponds to extremes, which can coexist in a developmental continuum within the same root structure (Smith and Read, 2008). Their formation depends partly on genotypic factors characterizing both partners (Karandashov et al., 2004), partly on environmental factors like phosphate availability (Smith and Read, 2008). In potato roots, both mycorrhizal types and root colonization are commonly influenced by phosphate concentration (McArthur and Knowles, 1992). This is in agreement with our observations since P concentration inhibited mycorrhizal development in a dose-dependent way, and *Arum-*type structures were occurring at low to medium P concentration (1, 10, and 50 ppm), while *Paris*-type formation appeared at higher P levels (100 and 300 ppm).

Our data on mycorrhized potato suggest that AOX or AOX-related metabolism is involved in arbuscule/hyphal branching and *Arum*-type formation, while COX or COX-related metabolism is associated with higher hyphal growth and hyphal-coiled shape (*Paris*-type) formation. Despite the use of SHAM being controversial because of its possible non-specificity to AOX (Bingham and Stevenson, 1995; Day et al., 1996), our results showed differential and opposite phenotypic patterns in plant and fungal behavior when SHAM and KCN treatments were compared. KCN and ABA are known to stimulate AOX activity (Finkelstein et al., 1998; Choi et al., 2000; Rook et al., 2006; Giraud et al., 2009; Millar et al., 2011; Lynch et al., 2012; Wind et al., 2012). These two molecules promoted both arbuscule intensity and branching, but they caused differential mycorrhizal development (M %), with ABA treated plants yielding a higher M % (with colonization in the primary adventitious root recognized under the experimental conditions even up to the base stem). This could be explained by the fact that COX capacity is not inhibited by ABA unlike KCN. On the other hand, we observed that ABA (Experiment 2) tended to promote the AOX gene transcript levels in non-inoculated potato plantlets, but such response was not clearly identified when using KCN (Experiment 3). It would therefore suggest that the mycorrhizal root colonization, but also transcript regulation of the AOX pathway, are dependent from the functional state on the COX pathway. To summarize, we can deduce that mycorrhizal behavior seems to be linked to the mitochondrial respiratory chain-partitioning environment, probably generated from both partners. As a remark, ethylene is another stress hormone that is able to induce AOX pathway (Simons et al., 1999; Wang et al., 2010; Xu F. et al., 2012), but usually impairs mycorrhizal colonization (using epinastic plant or exogenous application, Zsögön et al., 2008; Fracetto et al., 2013). This phenomenon could be partly explained by the action of cyanide (HCN) produced stoichiometrically (1:1) with ethylene, blocking therefore the COX pathway (Xu F. et al., 2012).

Interpretation of the sporulation rise at 50 ppm P in M plants is challenging, but it seems linked with specific plant metabolism independent of mycorrhizal colonization (M %), as already observed by past studies (Douds and Schenck, 1990), while this last parameter harbored high correlation with P concentration. A particularity is observed at 50 ppm P concentration through the various plant phenotypical parameters studied (plant growth parameters – Supplementary Figure S2; mycorrhizal dependency – **Figure 8**) and corresponds to the highest value of arbuscule intensity (a %, Supplementary Figure S1) with a predominant *Arum*-type, concomitant with an up-regulation of *RiAOX* transcripts (**Figure 9**) and the highest sporulation (**Figure 5**).

**FIGURE 11:**
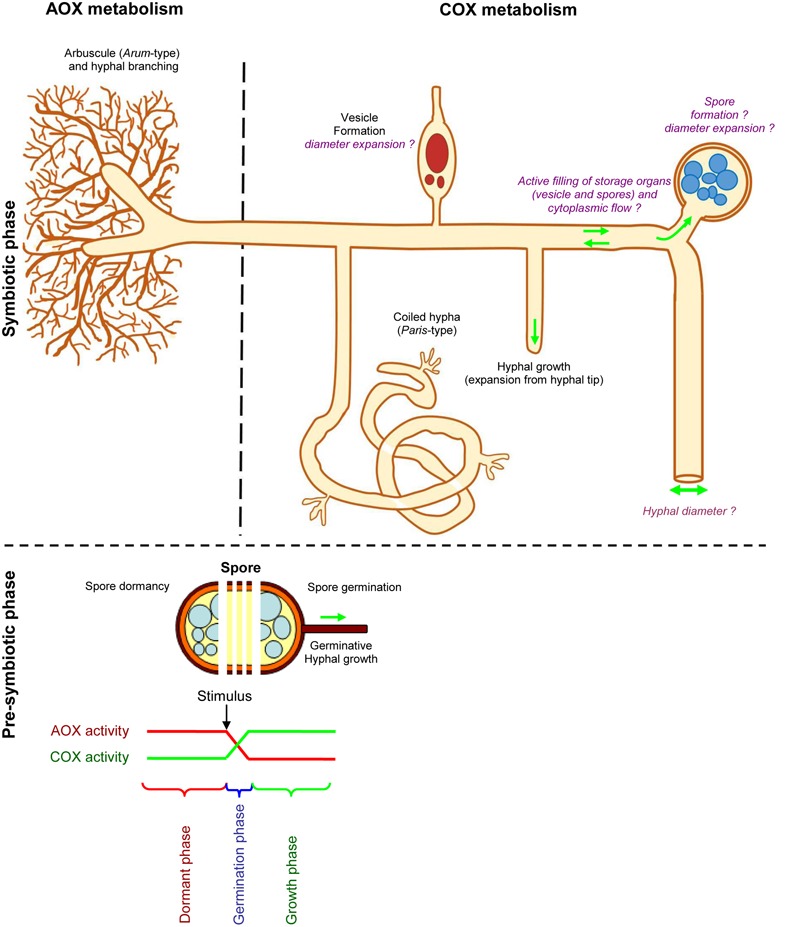
**Scheme describing potential roles of AOX and COX metabolism in arbuscular mycorrhizal fungi.** This scheme was drawn according to the fungal phenotype observed with SHAM and KCN treatments under *in vitro* and *ex vitro* assays. Compared to untreated plants and KCN treatment, SHAM decreases arbuscule intensity within mycorrhizal root fragment (a %), with predominance of *Paris*–type development, while vesicles intensity remains similar to non-treated plants. Compared to SHAM, KCN plants harbored reduced vesicle within root fragment (v %) and hyphal density (H %), but higher arbuscule formation in mycorrhizal root fragments (a %). Note that the inhibitors response on fungal parameters may differ according to specific P concentration. Spore germination is inhibited by ABA, SHAM, and KCN but enhanced by Ga3. We propose that spore germination corresponds likely to a punctual event involving respiratory shift that transfers a dormant state (likely linked with AOX pathway) to an active state (likely linked with COX pathway) allowing the fungus to explore and reach a host plant root (see text in section, Mitochondrial Respiratory Inhibitors Influence Fungal Behavior at Pre-symbiotic Phase). However, the respiratory chain pathway involved in spore formation and size, hyphal diameter, as well as the filling of storage organs and active cytoplasmic flow in AMF remains unknown. We suggest that COX metabolism might be preferentially expressed in these different processes that require energy (suggestions are written in purple).

Taking into account all these observations, we propose a scheme defining the roles of AOX and COX in the different mycorrhizal phenotypical behaviors (**Figure 11**).

### Mitochondrial Respiratory Inhibitors Induced Opposite Mycorrhizal Growth Dependency

Although many factors can influence the MGD, it is usually recognized that high P concentrations decrease MGD, which may become negative (Smith and Smith, 2011a). In our study, no correlation was found between MGD and P concentration, but respiratory inhibitors induced opposite responses. All possible MGD responses (neutral for non-treated plants, positive under KCN and negative under SHAM) were observed within the same AMF-plant biosystem, illustrating the high plasticity of mycorrhizal behavior linked to the physiological context. We show the possibility to obtain increasing yield with increasing P concentration when respiration in the plant–AMF system is associated with AOX pathway (i.e., in KCN treatments), occurring with a low fungal colonization and associated with relatively stable and almost maximal positive MGD. This goes together with a predominant *Arum*-type structures and higher arbuscule intensity when compared to treatments where COX pathway would be engaged (i.e., in SHAM treatments), in which *Paris*-type structure is predominant and associated with a negative MGD. This is consistent with the study of van Aarle et al. (2005), showing that *Arum*-type structures had higher metabolic activity than *Paris*-type ones. Moreover, even if SHAM reduced the MGD and the arbuscule intensity, while favoring *Paris*-type structures, the AMF produced a denser mycelium (h %) and as many vesicles as in non-treated mycorrhizal plants (except at 1 ppm P). AMF responses on plant performance seem therefore to depend mainly on the ability to form hyphal branching (*Arum*-type), possibly by an increased exchange surface for providing nutrients to plants (Kobae and Hata, 2010; Bapaume and Reinhardt, 2012). However, if MGD responses seem related with arbuscule type in KCN and SHAM treatments, no direct relationship was found between arbuscule intensity and MGD. This leads to the hypothesis that although various membrane transporters (phosphate, amino acid, sugar or nitrogen) were characterized in arbuscules, these fungal structures might not constitute preferential sites for nutrient/element exchanges, and a role should be also attributed to intraradical hypha. This assumption is supported by the fact that glucose transporters were observed not only in arbuscules but also on intraradical hyphae (Helber et al., 2011).

The mycorrhizal dependency variation has been attributed to plant species and cultivars, fungal species and isolates, host and symbiont interplay, mycorrhizal rate, soil phosphorus concentration and environmental conditions (Plenchette et al., 1983; Singh, 2001; Smith and Smith, 2011a). Our data suggest that the electron flow partitioning, which is modulated by environmental stimuli and genetic background, corresponds to a main metabolic component determining the MGD related to the mycorrhizal type. Finally, AOX metabolism, linked usually with higher ABA content, is known to be related to plant growth regulation and has been reported to be connected to growth depression (Sieger et al., 2005). This may partly explain the negative MGD phenomenon that AMF might trigger during early developmental stages in some plant species (Koide, 1985; Graham and Abbott, 2000; Smith et al., 2009; Ronsheim, 2012) during the implementation of the induced systemic response in the plant (Pozo et al., 2002; Hause and Fester, 2005; Hause et al., 2007).

### Mitochondrial Respiratory Inhibitors Influence Symbiotic Nutrient Transport

Arbuscular mycorrhizal inducible Pi transporters of the Pht1 family have been described as markers of mycorrhizal development and, in some cases, proposed as markers of mycorrhizal functionality (Javot et al., 2007). Partly in accordance to this statement, our data show a correlation between AM-inducible transporter expression (*StPT3, StPT4*, and *StPT5*) and the mycorrhizal development. A gradual repression of the MPU pathway with increasing P availability in non-treated mycorrhizal plants was also observed, which is in accordance with several previous studies on *Solanaceae* species (Chen et al., 2007; Nagy et al., 2009), suggesting that the MPU is not functional at high P levels. However, the repression of the MPU by P disappeared when plants were treated with KCN, and surprisingly all AM-inducible P transporters were strongly up-regulated at 300 ppm in non-inoculated plants. This last observation deserves further investigations, as no previous works have been done with very high P concentrations to our knowledge.

The mycorrhizal impact on DPU is less clear. A number of studies have shown that AM colonization of plants down-regulates the expression of the DPU Pi transporters (Rausch et al., 2001; Burleigh et al., 2002; Glassop et al., 2005; Requena, 2005; Nagy et al., 2006), while other studies pointed contrasted transcriptional regulations. Chen et al. (2007) showed a mycorrhiza-induced down-regulation of *Pht1;1* and *Pht1;2* expression under low-P conditions, but up-regulation of *Pht1;2* under high-P conditions for pepper, eggplant and tobacco. In tomato, Nagy et al. (2009) did not find any change in the transcript abundance for *SlPT1* and *SlPT2* upon root colonization. Our data are in accordance with these latter observations, as presence of AMF did not necessarily induced down-regulation of non-AM-inducible transporters, but responses were specific to P concentrations. For example, it was noticed that AMF induced an up-regulation at 100 ppm P but a down regulation at 10 ppm P for *StPT1*, while an up-regulation of *StPT2/6* was observed at 100 ppm P. Only *StPT2/6* was regulated by P availability. KCN treatment induced an up-regulation of DPU transporters in mycorrhizal conditions.

Fungal Pht1 genes are known to be expressed in the intraradical phase (Harrison and van Buuren, 1995; Benedetto et al., 2005; Balestrini et al., 2007; Tisserant et al., 2012; Fiorilli et al., 2013; Walder et al., 2016). Fiorilli et al. (2013) showed a slight down-regulation of *RiPT1* when exposed to higher P concentrations, and Walder et al. (2016) reported a positive correlation of *RiPT5* transcript levels and Pi acquisition in Sorghum. In our study, only *RiPT5* was slightly regulated by P levels, with up-regulation in concentrations higher than 50 ppm in non-treated conditions. Few data exist on the translocation of Pi across the inner mitochondrial membrane by *Pht3* family in plants and fungi. In our study, no clear role emerged for these transporters within AMF symbiosis. *StMPT3*, which presented the highest expression levels in the roots, appeared to be constitutive, and only *StMPT1a* was slightly up-regulated by the presence of *R. irregulare* in non-treated conditions. *RiMPT* appeared also to be constitutively expressed in our experimental conditions.

In plants, several transcriptomic analyses revealed that AM establishment can induce the expression of plant N transporters, mainly in arbusculated cells (Gomez et al., 2009; Guether et al., 2009; Kobae et al., 2010; Gaude et al., 2012; Ruzicka et al., 2012). AMTs identified in tomato *LeAMT4* and *LeAMT5* were reported to be exclusively expressed in mycorrhizal roots and not regulated by NH_4_^+^ (Ruzicka et al., 2012). The two potato homologs, *StAMT4* and *StAMT5*, were strongly up-regulated by AMF, and were gradually repressed by P in mycorrhizal plants. This decrease in transcript levels can be attributed to the reduction of fungal structures inside the roots. However, in the case of AM-inducible AMTs, such as AM-inducible Pht1, NM plants presented a strong up-regulation at a high P level (300 ppm). As for P transporters, a suppression of this up-regulation at 300 ppm P in the presence of KCN was observed and could be explained by the need of a functional COX pathway for ammonium and phosphate transport. NH_4_^+^ uptake via AMT seems indeed to be accompanied by H^+^ extrusion by the plasma membrane H^+^-ATPases for maintenance of the cytosolic charge balance, and increased fluxes of NH_4_^+^ would increase the demand for respiratory ATP (Britto and Kronzucker, 2005). Hachiya et al. (2010) suggested that the ammonium-dependent increase of the O_2_ uptake rate can be explained by the up-regulation of the cytochrome pathway, which may be related to the ATP consumption by the plasma-membrane H^+^-ATPases. Similarly, P transport is a proton-dependent process, and the H^+^-ATPase *HA1* of *Medicago truncatula* was shown as essential for P transport in AM symbiosis (Krajinski et al., 2014). *StHA1* is also up-regulated at 300 ppm P in non-inoculated plants compared to 1 ppm P and repressed in mycorrhizal plants. It can be therefore hypothesized that an impaired COX pathway would repress ammonium transport via AMT gene family members.

P and N AM-inducible transporters were found to be connected to the mycorrhizal development in non-treated plants. On the other hand, no correlation was observed between the expression of P and N AM-inducible transporters and the presence of either specific fungal structures (arbuscule or intraradical hypha) or any MGD parameters across the treatments (Supplementary Tables S2–S4). In particular, M KCN plants were associated with positive MGD compared to NM KCN plants but the transcript levels of genes encoding AM-inducible transporters were similar to M SHAM treatments. Several studies showed a lack of relationship between *Pht1* gene expression and mycorrhizal Pi acquisition (Grace et al., 2009; Grønlund et al., 2013; Walder et al., 2015). It would therefore indicate that MGD and that plant’s Pi acquisition through the MPU is not quantitatively regulated by the expression level of AM-inducible *Pht1* genes. These observations, combined with the lack of repression of the MPU by P in presence of KCN, and the strong up-regulation of AM-inducible P transporter genes at 300 ppm in NMs, suggest that these transporters are not suitable markers for a functional symbiosis in field conditions where high P concentrations can occur and natural or anthropogenic source of cyanides can be found in soil or water (with concentrations as high as 100 mg kg DW^-1^).

### What about the Metabolic Role of AOX and COX in AMF?

Despite sugar transporters being found and characterized (Helber et al., 2011), AMF are still unable to complete their life cycle even when carbon sources are added in *in vitro* culture systems without the presence of plant roots. Some publications showed even the opposite: glucose and fructose application under axenic or monoxenic conditions resulted in reduced fungal growth or spore germination rate (Mosse, 1959; Koske, 1981; Hepper, 1982; Siquiera and Hubbell, 1986; Wang et al., 2015), while culture media devoid of sucrose can stimulate spore germination, hyphal growth (D’Souza et al., 2013), as well as, sporulation (St-Arnaud et al., 1996). In many publications, the definition of obligate biotrophy of AMF deals with their dependency on plants for carbohydrate supply (mainly glucose and fructose, Pfeffer et al., 1999). But obviously, this statement seems incomplete since it is not yet strictly proven by the successful implementation of an axenic culture. Genomic and transcriptomic data obtained from *R. irregulare* were not helpful since this fungal species would possess all the necessary genes to harbor saprobe behavior (Tisserant et al., 2012; Tisserant et al., 2013), although no evidence for gene encoding for fungal multi-domain fatty acid synthase was found (Wewer et al., 2014). Therefore, the fungal needs related to the biotrophy of AMF remains still an open question. In this way, it seems important to emphasize that plants provide, at least, a habitat, i.e., a physical growth support associated with a favorable physiological frame allowing uptake and metabolic assimilation of carbon sources that AMF seems not to encounter without host. This questions the definition of the metabolic frame (induced by stress signals) favorable to AMF colonization, arbuscule development and functions on plant performances. We discuss below the role of COX and AOX, which can shed new light that would allow a better understanding and mastering of mycorrhiza.

#### COX Pathway and Aerobic Respiration

In non-inoculated plants, fresh biomass and potato yield are repressed by KCN and enhanced by SHAM (especially at 100 and 300 ppm P). This suggests that the tuberization is an active COX-dependent process needing O_2_, which is consistent with the high O_2_ concentration requirement observed previously for tuber formation (Saini, 1976; Hooker, 1981; Cary, 1985; Geigenberger et al., 2000). This fits also with the reported effect of SHAM, known to optimize the O_2_ flow through plant tissues (Sesay et al., 1986; Spreen Brouwer et al., 1986; Møller et al., 1988; Gupta et al., 2009). The positive MGD on yield observed in KCN treatment suggests that AMF improve plant respiration, not only related to P concentration, but also to tissue oxygenation, in order to sustain both COX needs and a better carbon allocation in the tuber. However, our expression data obtained at harvest for *StCOXVb* and *StCytc1* genes are difficult to interpret in link with these assertions. *StCytc1* expression is up-regulated in NM KCN plants compared to NM SHAM plants at 10 and 100 ppm P, but it remains similar for the other P concentrations tested, suggesting probable other regulation levels related to COX pathway.

Potassium cyanide impacted negatively intraradical hyphal growth and vesicle formation, likely by blocking O_2_ consumption needed for fungal COX metabolism. It is known that anaerobic conditions in flooded soils is associated with reduced mycorrhizal colonization (Miller and Sharitz, 2000). Moreover, arbuscules are resistant to KCN when compared to non-treated plants, but their intensity is promoted compared to SHAM in all P concentrations, increasing, therefore, the arbuscule/intraradical hypha ratio. This is consistent with the results of Saif (1981), where soil O_2_ content was correlated with the mycorrhizal development and vesicles production, and arbuscule occurrence was observed only at specific O_2_ concentration but not under soil O_2_ excess. Depression of arbuscule intensity by SHAM might be related to a higher O_2_ availability for AMF respiration in roots. The sensitivity of arbuscules to O_2_ raises the possibility that they might be a respiration structure where gas exchanges between both partners might occur. Soils have usually less available O_2_ (Clark, 1967; Bhattarai et al., 2005) and are enriched in CO_2_ compared to ambient atmosphere (Bouma and Bryla, 2000; Pfanz et al., 2004). As Miller and Bever (1999) suggested, O_2_ could be provided by the plant and mobilized for COX-metabolism needs for fungal growth. The increase of mycorrhizal development in ABA treated plants could be linked to a promoted plant COX pathway potential, this hypothesis being supported by the induction of *StCytc1* gene in M+ABA treatment.

#### AOX Pathway and Fermentation

Our data showed that AOX is involved in specific plant and fungal behavior. However, its energetic metabolic significance is not yet well understood. In order to better interpret its functions within mycorrhizal symbiosis, we thought it was relevant to enlighten possible connections with fermentation, as one “AOX-related key metabolism.”

Fermentation pathways use pyruvate as source that can be reduced to lactate (by LDH) in presence of reducing potential (NADH) or processed into CO_2_ and acetaldehyde (by PDC, a thiamine pyrophosphate-dependent enzyme), which is then reduced into ethanol (alcohol dehydrogenase) or acetate (aldehyde dehydrogenase), also in presence of NADH. Pyruvate can be synthesized from glucose (classic glycolytic pathway) or from malate (alternative glycolytic pathway) depending on the pH-stat and therefore redox potential (Sakano, 2001). Several clues from previous metabolic studies suggest a possible link between AOX and fermentation in plants but also in fungi and are detailed in Supplementary Material (Section 3).

In our study, P concentration was not directly correlated with fermentation in non-treated conditions. But we noticed in Experiment 3 an induction of *StLDH1* and *StPDC3* occurring at several P concentrations, when comparing KCN to SHAM treatments. Moreover, our data from the ABA test showed that expression of genes involved in fermentation in non-inoculated plants were associated with the higher expression of the genes encoding for AOX isoforms and lower *StCytc1*. These observations would indicate persistent implementation of the fermentative system under stressed conditions, which was expected in these situations.

From our data, the sporulation pattern in non-treated plants was not correlated with mycorrhizal colonization, while this last parameter harbored high correlation with P concentration. This could suggest that spore formation and mycorrhizal development, requiring obviously energy for their development, may need the involvement of at least two carbon source partitioning. We noticed a significant correlation between sporulation and the *StLDH1* profile, both showing, with the *RiAOX* transcripts, maximum values at 50 ppm P (non-treated plants). At the same time, the presence of AMF reduced significantly the total biomass only at 50 ppm P without disturbing the potato yield biomass indicating influence of a specific stress. This assumption is supported by a down-regulation of *StCytc1* but an up-regulation of *StAOX2* (comparing M/NM plants in Experiment 3). Then, ABA data suggest that a higher mycorrhizal colonization is the consequence of an initial physiological stress state implemented in plant prior to root contact by AMF, in which AOX and fermentation seems to take place.

This connection between AOX-metabolism and fermentation leads to the hypothesis that AMF might partly stimulate plants to provide – or generate a metabolic environment that favors the fungal synthesis of – fermentation products as possible carbon sources for its storage needs. This assumption seems, to our knowledge, underestimated since no publication discusses directly these aspects on AMF, although it is known that presence of fermentative organisms/fermented products in soil can stimulate mycorrhizal development (Medina et al., 2007, 2010; Azcón et al., 2013), providing a clue. In particular, our data suggest that lactate, as one energy source for fungal needs, seems to be a good candidate, involved perhaps in sporulation potentials and arbuscule formation. Attempting to give a little more insight, first tests using exogenous lactate on potato plants under greenhouse conditions showed that it was possible to increase significantly the mycorrhizal colonization and arbuscule intensity associated with a positive MGD (Mercy, personal observation). As a remark, attention should be paid in further works to the role of the alternative glycolytic pathway (NAD/NADP malic enzymes) in mycorrhizal behavior, as it involves malate as source for fermentation and AOX pathway, both engaged during P-deficiency, a stress condition usually favoring the mycorrhizal development.

### Open Questions and Perspectives

This is the first work that extensively studies phenotypic patterns by using respiratory chain inhibitors in mycorrhizal symbiotic associations. Plant growth parameters suggest that “AOX plants” (plants that are able to engage AOX pathway to a higher extent) are more positively responsive to AMF and have higher potentials in carbon allocation to the potato tuber than “COX plants” (plants that show a higher engagement of COX pathway), and plants treated with ABA or with KCN indicated that AMF inoculation is efficient to counteract extreme stresses in terms of growth performance. Under stress-related physiology, AOX might represent the main (or a more engaged) respiratory chain pathway in plant roots, concomitant with higher fermentation that might favor arbuscule development. In analogy to previous observations (Liu et al., 2015), we observed that the presence of AMF reduces plant stress and seems to favor the COX pathway, which is consistent with higher energetic metabolism that associates to positive MGD. Therefore, artificial soft and transitory induction techniques of AOX metabolism in plants, without disturbing COX potential, could represent a physiological target for producers for improving mycorrhizal plant susceptibility and mycorrhizal dependency.

It is very likely that the modulation of respiration partitioning occurs at different stages of AMF life cycle, as shown during presymbiotic phase by the induction of mitochondrial respiration by several plant root exudates (Tamasloukht et al., 2003; Besserer et al., 2008) and AOX expression along the symbiotic establishment (Campos et al., 2015). Optimal mycorrhizal development needs obviously oxygen (KCN data) but seems also related to fermentation (ABA data). This opens the possibility that AMF, at a whole, might belong to obligate aerobic fermentative organisms. Therefore, the role of fermentation in mycorrhizal behavior deserves further investigations providing potentially exciting new avenues for next studies.

While the application of respiratory inhibitors induced significant changes from phenotypical parameters and the expression of some genes involved in transport and fermentation, interpretation of transcriptomic data related to AOX and COX pathways was challenging. However, respiration genes are hierarchically superimposed to specific metabolic pathway genes. Increasing data supports the hypothesis that the most crucial responses of AOX genes to changing environments are induced early during cell reprogramming before specific pathways are initiated and interfere with growth performance (Arnholdt-Schmitt et al., 2006). Recently, this was confirmed for the carrot anti-freezing protein gene under cold treatment (Campos et al., 2016). Furthermore, in *A. thaliana*, it was shown that limitation of the capacity of the alternative-, cytochrome-, or both termini-oxidases results in unique and overlapping transcriptional responses depending on growth conditions. Nevertheless, this research presented the first clues for the implication of respiratory metabolism in a mycorrhizal symbiosis seen as a holobiont level system. Thus, our research supports further deeper molecular-physiological studies on respiration traits and exploiting genetic approaches through overexpressing and knock-out or respiratory gene editing techniques. However, final progress in order to associate complex respiration metabolism to robust growth performance will absolutely depend also on the development of adequate predictive and phenotyping-screening technologies (Fiorani and Schurr, 2013; Arnholdt-Schmitt et al., 2014, 2016; Nogales et al., 2016).

## Author Contributions

LM and EL-M conceptualized the experimental design and contributed equally to research, trial conductance and data interpretation. BA-S initiated research on the role of AOX on plant–AMF interaction, developed and coordinated together with CS the supporting IAPP research project (AGRO-AMF-AOX). AN contributed to data interpretations. AP was involved in the trial performance and contributed to data interpretations. LM and EL-M designed and wrote the manuscript and all the authors revised and approved it.

## Conflict of Interest Statement

The authors declare that the research was conducted in the absence of any commercial or financial relationships that could be construed as a potential conflict of interest. The reviewer AS and handling Editor declared their shared affiliation, and the handling Editor states that the process nevertheless met the standards of a fair and objective review.

## Footnotes

1 http://glomeromycota.wixsite.com/lbmicorrizas/cpia

2 http://genome.jgi.doe.gov/Gloin1/Gloin1.home.html

3 http://potato.plantbiology.msu.edu/integrated_searches.shtml

4 http://mycor.nancy.inra.fr/IMGC/GlomusGenome/index3.html

5 http://genome.jgi.doe.gov/

6 http://www.ncbi.nlm.nih.gov/genbank/

## Abbreviations

ABA: abscisic acid; AM-inducible transporter, arbuscular mycorrhizal inducible transporter
AMF: arbuscular mycorrhizal fungi
AMT: ammonium transporter
AOX: alternative oxidase
COX: cytochrome oxidase
DAI: day after inoculation
DPU: direct phosphate uptake
FW: fresh weight
Ga3: gibberellic acid
HA: H+-ATPase
INT: iodonitrotetrazolium salt
KCN: potassium cyanide
LDH: lactate dehydrogenase
M: mycorrhizal plant
MGD: mycorrhizal growth dependency
MPT: mitochondrial phosphate transporter
MPU: mycorrhizal phosphate uptake
NM: non-mycorrhizal plant
P: phosphorus
PDC: pyruvate decarboxylase
ppm: part per million
PT: phosphate transporter
SHAM: salicylhydroxamic acid
WAI: week after inoculation
Mycorrhizal parameters: F%: frequency of mycorrhiza
M%: intensity of mycorrhiza in whole root system
m%: intensity of mycorrhiza in mycorrhizal root fragment
A%: intensity of arbuscules in whole root system
a%: intensity of arbuscules in mycorrhizal root fragment
V%: intensity of vesicles in whole root system
v%: intensity of vesicles in mycorrhizal root fragment
H%: intensity of hyphae in whole root system
h%: intensity of hyphae in mycorrhizal root fragment

## References

[B1] Andersen C. L. Jensen J. L. Orntoft T. F. (2004). Normalization of real-time quantitative reverse transcription-PCR data: a model-based variance estimation approach to identify genes suited for normalization, applied to bladder and colon cancer data sets. *Cancer Res.* 64 5245–5250. 10.1158/0008-5472.CAN-04-0496 15289330

[B2] Arnholdt-Schmitt B. Costa J. H. Fernandes de Melo D. (2006). AOX - a functional marker for efficient cell reprogramming under stress? *Trends Plant Sci.* 11 281–287. 16713324 10.1016/j.tplants.2006.05.001

[B3] Arnholdt-Schmitt B. Hansen L. D. Nogales A. (2016). Calorespirometry, oxygen isotope analysis and functional-marker-assisted selection (’CalOxy-FMAS’) for genotype screening: a novel concept and tool kit for predicting stable plant growth performance and functional marker identification. *Brief. Funct. Genomics* 5 10–15. 10.1093/bfgp/elv008 25818699

[B4] Arnholdt-Schmitt B. Valadas V. Doering M. (2014). Functional marker development is challenged by the ubiquity of endophytes – a practical perspective. *Brief. Funct. Genomics* 15 16–21. 10.1093/bfgp/elu049 25526729

[B5] Aroca R. Porcel R. Ruiz-Lozano J. M. (2007). How does arbuscular mycorrhizal symbiosis regulate root hydraulic properties and plasma membrane aquaporins in *Phaseolus vulgaris* under drought, cold or salinity stresses? *New Phytol.* 173 808–816. 10.1111/j.1469-8137.2006.01961.x 17286829

[B6] Aroca R. Ruiz-Lozano J. M. Zamarreño A. M. Paz J. A. García-Mina J. M. Pozo M. J. (2013). Arbuscular mycorrhizal symbiosis influences strigolactone production under salinity and alleviates salt stress in lettuce plants. *J. Plant Physiol.* 170 47–55. 10.1016/j.jplph.2012.08.020 23102876

[B7] Azcón R. Medina A. Aroca R. Ruiz-Lozano J. M. (2013). “Abiotic stress remediation by the arbuscular mycorrhizal symbiosis and rhizosphere bacteria/yeast interactions,” in *Molecular Microbial Ecology of the Rhizosphere* 1st Edn Vol. 2 ed. de Bruijn F. (Oxford: John Wiley & Sons, Inc.) 991–1002.

[B8] Bago B. Pfeffer P. E. Abubaker J. Jun J. Allen J. W. Brouillette J. (2003). Carbon export from arbuscular mycorrhizal roots involves the translocation of carbohydrate as well as lipid. *Plant Physiol.* 131 1–11. 10.1104/pp.102.007765 PMC166909 12644699

[B9] Balestrini R. Gomez-Ariza J. Lanfranco L. Bonfante P. (2007). Laser microdissection reveals that transcripts for five plant and one fungal phosphate transporter genes are contemporaneously present in arbusculated cells. *Mol. Plant Microbe Interact.* 20 1055–1062. 10.1094/MPMI-20-9-1055 17849708

[B10] Bapaume L. Reinhardt D. (2012). How membranes shape plant symbioses: signaling and transport in nodulation and arbuscular mycorrhiza. *Front. Plant Sci.* 3:223 10.3389/fpls.2012.00223 PMC3464683 23060892

[B11] Bedini S. Pellegrino E. Avio L. Pellegrini S. Bazzoffi P. Argese E. (2009). Changes in soil aggregation and glomalin–related soil protein content as affected by the arbuscular mycorrhizal fungal species *Glomus mosseae* and *Glomus intraradices*. *Soil Biol. Biochem.* 41 1491–1496. 10.1016/j.soilbio.2009.04.005

[B12] Benedetto A. Magurno F. Bonfante P. Lanfranco L. (2005). Expression profiles of a phosphate transporter gene (GmosPT) from the endomycorrhizal fungus *Glomus mosseae*. *Mycorrhiza* 15 620–627. 10.1007/s00572-005-0006-9 16133249

[B13] Berridge M. V. Tan A. S. Herst P. M. (2005). Tetrazolium dyes as tools in cell biology: new insights into their cellular reduction. *Biotechnol. Annu. Rev.* 11 127–152. 10.1016/S1387-2656(05)11004-7 16216776

[B14] Berruti A. Lumini E. Balestrini R. Bianciotto V. (2016). Arbuscular mycorrhizal fungi as natural biofertilizers: let’s benefit from past successes. *Front. Microbiol.* 6:1559 10.3389/fmicb.2015.01559 PMC4717633 26834714

[B15] Besserer A. Becard G. Jauneau A. Roux C. Séjalon-Delmas N. (2008). GR24, a synthetic analog of strigolactones, stimulates the mitosis and growth of the arbuscular mycorrhizal fungus *Gigaspora rosea* by boosting its energy metabolism. *Plant Physiol.* 148 402–413. 10.1104/pp.108.121400 18614712 PMC2528133

[B16] Besserer A. Becard G. Roux C. Sejalon-Delmas N. (2009). Role of mitochondria in the response of arbuscular mycorrhizal fungi to strigolactones. *Plant Signal. Behav.* 4 75–77. 10.4161/psb.4.1.7419 19704715 PMC2634080

[B17] Besserer A. Puech-Pages V. Kiefer P. Gomez-Roldan V. Jauneau A. Roy S. (2006). Strigolactones stimulate arbuscular mycorrhizal fungi by activating mitochondria. *PLoS Biol.* 4:E226 10.1371/JOURNAL.PBIO PMC1481526 16787107

[B18] Bhattarai S. P. Su N. Midmore D. J. (2005). Oxygenation unlocks yield potentials of crops in oxygen limited soil environments. *Adv. Agron.* 88 313–377. 10.1016/S0065-2113(05)88008-3

[B19] Bingham I. J. Stevenson E. A. (1995). Causes and location of non–specific effects of SHAM on O2 uptake by wheat roots. *Physiol. Plant.* 93 427–434. 10.1111/j.1399-3054.1995.tb06839.x

[B20] Bouma T. J. Bryla D. R. (2000). On the assessment of root and soil respiration for soils of different textures: interactions with soil moisture contents and soil CO2 concentrations. *Plant and Soil* 227 215–221. 10.1023/A:1026502414977

[B21] Breuillin F. Schramm J. Hajirezaei M. Ahkami A. Favre P. Druege U. (2010). Phosphate systemically inhibits development of arbuscular mycorrhiza in Petunia hybrida and represses genes involved in mycorrhizal functioning. *Plant J.* 64 1002–1017. 10.1111/j.1365-313X.2010.04385.x 21143680

[B22] Britto D. T. Kronzucker H. J. (2005). Nitrogen acquisition, PEP carboxylase, and cellular pH homeostasis: new views on old paradigms. *Plant Cell Environ.* 28 1396–1409. 10.1111/j.1365-3040.2005.01372.x

[B23] Brundrett M. C. (2002). Coevolution of roots and mycorrhizas of land plants. *New Phytol.* 154 275–304. 10.1046/j.1469-8137.2002.00397.x 33873429

[B24] Bucher M. (2007). Functional biology of plant phosphate uptake at root and mycorrhiza interfaces. *New Phytol.* 173 11–26. 10.1111/j.1469-8137.2006.01935.x 17176390

[B25] Burleigh S. H. Cavagnaro T. Jakobsen I. (2002). Functional diversity of arbuscular mycorrhizas extends to the expression of plant genes involved in P nutrition. *J. Exp. Bot.* 53 1593–1601. 10.1093/jxb/erf013 12096098

[B26] Campos C. Cardoso H. Nogales A. Svensson J. Lopez-Ráez J. A. Pozo M. J. (2015). Intra and inter-spore variability in *Rhizophagus irregularis* AOX gene. *PLoS ONE* 10:e0142339 10.1371/journal.pone PMC4634980 26540237

[B27] Campos M. D. Nogales A. Cardoso H. G. Rajeev Kumar S. Nobre T. Sathishkumar R. (2016). Stress-induced accumulation of DcAOX1 and DcAOX2a transcripts coincides with critical time point for structural biomass prediction in carrot primary cultures (*Daucus carota* L.). *Front. Genet* 7:1 10.3389/fgene.2016.00001 PMC4731517 26858746

[B28] Cano-Canchola C. Escamilla E. Ruiz-Herrera J. (1988). Environmental control of the respiratory system in the dimorphic fungus *Mucor rouxii*. *J. Gen. Microbiol.* 134 2993–3000. 10.1099/00221287-134-11-2993

[B29] Cary J. W. (1985). Potato tubers and soil aeration. *Agron. J.* 77 379–383. 10.2134/agronj1985.00021962007700030007x

[B30] Charest C. Dalpé Y. Brown A. (1993). The effect of vesicular arbuscular mycorrhizae and chilling on two hybrids of *Zea mays* L. *Mycorrhiza* 4 89–92. 10.1007/BF00204064

[B31] Chen A. Chen X. Wang H. Liao D. Gu M. Qu H. (2014). Genome-wide investigation and expression analysis suggest diverse roles and genetic redundancy of Pht1 family genes in response to Pi deficiency in tomato. *BMC Plant Biol.* 14:61 10.1186/1471-2229-14-61 PMC4007770 24618087

[B32] Chen A. Hu J. Sun S. Xu G. (2007). Conservation and divergence of both phosphate- and mycorrhiza-regulated physiological responses and expression patterns of phosphate transporters in solanaceous species. *New Phytol.* 173 817–831. 10.1111/j.1469-8137.2006.01962.x 17286830

[B33] Choi H. Hong J. Ha J. Kang J. Kim S. Y. (2000). ABFs, a family of ABA–responsive element binding factors. *J. Biol. Chem.* 275 1723–1730. 10.1074/jbc.275.3.1723 10636868

[B34] Clark F. E. (1967). “Bacteria in soil,” in *Soil Biology* eds Burges A. Raw F. (London: Academic Press) 15–49.

[B35] Costa J. H. McDonald A. E. Arnholdt-Schmitt B. Fernandes de Melo D. (2014). A classification scheme for alternative oxidases reveals the taxonomic distribution and evolutionary history of the enzyme in angiosperms. *Mitochondrion* 19 172–183. 10.1016/j.mito.2014.04.007 24751423

[B36] Day D. A. Krab K. Lambers H. Moore A. L. Siedow J. N. Wagner A. M. (1996). The cyanide resistant oxidase: to inhibit or not to inhibit, that is the question. *Plant Physiol.* 110 1–2. 10.1104/pp.110.1.1 12226168 PMC157687

[B37] Douds D. D. Schenck N. C. (1990). Relationship of colonization and sporulation by VA mycorrhizal fungi to plant nutrient and carbohydrate contents. *New Phytol.* 116 621–627. 10.1111/j.1469-8137.1990.tb00547.x

[B38] Draper N. R. Smith H. (1998). *Applied Regression Analysis* 3rd Edn. New York, NY: Wiley 10.1002/9781118625590

[B39] D’Souza J. Rodrigues K. M. Rodrigues B. F. (2013). Modified Strullu and Romand (MSR) medium devoid of sucrose promotes higher in vitro germination in *Rhizophagus irregularis*. *J. Mycol. Plant Pathol.* 43 240–242.

[B40] Finkelstein R. R. Wang M. L. Lynch T. J. Rao S. Goodman H. M. (1998). The Arabidopsis abscisic acid response locus ABI4 encodes an APETALA 2 domain protein. *Plant Cell* 10 1043–1054. 10.1105/tpc.10.6.1043 9634591 PMC144030

[B41] Fiorani F. Schurr U. (2013). Future scenarios for plant phenotyping. *Annu. Rev. Plant Biol.* 64 267–291. 10.1146/annurev-arplant-050312-120137 23451789

[B42] Fiorilli V. Lanfranco L. Bonfante P. (2013). The expression of GintPT, the phosphate transporter of *Rhizophagus irregularis*, depends on the symbiotic status and phosphate availability. *Planta* 237 1267–1277. 10.1007/s00425-013-1842-z 23361889

[B43] Fracetto G. G. M. Peres L. E. P. Mehdy M. C. Lambais M. R. (2013). Tomato ethylene mutants exhibit differences in arbuscular mycorrhiza development and levels of plant defense-related transcripts. *Symbiosis* 60 155–167. 10.1007/s13199-013-0251-1

[B44] Gallaud J. (1905). Etude sur les mycorrhizes endotrophes. *Rev. Gén. Bot.* 17 5–48.

[B45] Gallou A. (2011). *Impact of Rhizophagus sp. (syn. Glomus sp.) and Trichoderma harzianum on the Potato Resistance Against Rhizoctonia solani and Phytophthora infestans, Two Major Potato Pathogens.* Ph.D. thesis, Université catholique de Louvain Louvain.

[B46] Gaude N. Bortfeld S. Duensing N. Lohse M. Krajinski F. (2012). Arbuscule-containing and non-colonized cortical cells of mycorrhizal roots undergo extensive and specific reprogramming during arbuscular mycorrhizal development. *Plant J.* 69 510–528. 10.1111/j.1365-313X.2011.04810.x 21978245

[B47] Geigenberger P. Fernie A. R. Gibon Y. Christ M. Stitt M. (2000). Metabolic activity decreases as an adaptive response to low internal oxygen in growing potato tubers. *Biol. Chem.* 381 723–740. 10.1515/BC.2000.093 11030430

[B48] Gerdemann J. W. Nicolson T. H. (1963). Spores of mycorrhizal *Endogone* species extracted from soil by wet sieving and decanting. *Trans. Br. Mycol. Soc.* 46 235–244. 10.1016/S0007-1536(63)80079-0

[B49] Gianinazzi S. Gollotte A. Binet M. N. van Tuinen D. Redecker D. Wipf D. (2010). Agroecology: the key role of arbuscular mycorrhizas in ecosystem services. *Mycorrhiza* 20 519–530. 10.1007/s00572-010-0333-3 20697748

[B50] Gianinazzi-Pearson V. Gianinazzi S. Trouvelot A. (1985). Evaluation of the infectivity and effectiveness of indigenous vesicular–arbuscular fungal populations in some agricultural soils in Burgundy. *Can. J. Bot.* 63 1521–1524. 10.1139/b85-210

[B51] Giovanetti M. Avio L. Sbrana C. (2010). “Fungal spore germination and pre–symbiotic mycelial growth – physiological and genetic aspects,” in *Arbuscular Mycorrhizas: Physiology and Function* eds Koltai H. Kapulnik Y. (Berlin: Springer) 3–32.

[B52] Giraud E. Van Aken O. Ho L. H. Whelan J. (2009). The transcription factor ABI4 is a regulator of mitochondrial retrograde expression of Alternative oxidase 1a. *Plant Physiol.* 150 1–33. 10.1104/pp.109.139782 PMC2705018 19482916

[B53] Glassop D. Smith S. E. Smith F. W. (2005). Cereal phosphate transporters associated with the mycorrhizal pathway of phosphate uptake into roots. *Planta* 222 688–698. 10.1007/s00425-005-0015-0 16133217

[B54] Gomez K. S. Javot H. Deewatthanawong P. Torres-Jerez I. Tang Y. Blancaflor E. B. (2009). Medicago truncatula and Glomus intraradices gene expression in cortical cells harboring arbuscules in the arbuscular mycorrhizal symbiosis. *BMC Plant Biol.* 9:10 10.1186/1471-2229-9-10 PMC2649119 19161626

[B55] Grace E. J. Cotsaftis O. Tester M. Smith F. A. Smith S. E. (2009). Arbuscular mycorrhizal inhibition of growth in barley cannot be attributed to extent of colonization, fungal phosphorus uptake or effects on expression of plant phosphate transporter genes. *New Phytol.* 181 938–949. 10.1111/j.1469-8137.2008.02720.x 19140934

[B56] Graham J. H. Abbott L. K. (2000). Wheat responses to aggressive and non–aggressive arbuscular mycorrhizal fungi. *Plant Soil* 220 207–218. 10.1023/A:1004709209009

[B57] Grahl N. Dinamarco T. M. Willger S. D. Goldman G. H. Cramer R. A. (2012). *Aspergillus fumigatus* mitochondrial electron transport chain mediates oxidative stress homeostasis, hypoxia responses and fungal pathogenesis. *Mol. Microbiol.* 84 383–399. 10.1111/j.1365-2958.2012.08034.x 22443190 PMC3323727

[B58] Grønlund M. Albrechtsen M. Johansen I. E. Hammer E. C. Nielsen T. H. Jakobsen I. (2013). The interplay between P uptake pathways in mycorrhizal peas: a combined physiological and gene-silencing approach. *Physiol. Plant.* 149 234–248. 10.1111/ppl.12030 23387980

[B59] Guether M. Neuhäuser B. Balestrini R. Dynowski M. Ludewig U. Bonfante P. (2009). A mycorrhizal-specific ammonium transporter from lotus japonicus acquires nitrogen released by Arbuscular Mycorrhizal Fungi. *Plant Physiol.* 150 73–83. 10.1104/pp.109.136390 19329566 PMC2675747

[B60] Gupta K. J. Zabalza A. van Dongen J. T. (2009). Regulation of respiration when the oxygen availability changes. *Physiol. Plant.* 137 383–391. 10.1111/j.1399-3054.2009.01253.x 19549068

[B61] Hachiya T. Watanabe C. K. Boom C. Tholen D. Takahara K. Kawai-Yamada M. (2010). Ammonium-dependent respiratory increase is dependent on the cytochrome pathway in *Arabidopsis thaliana* shoots. *Plant Cell Environ.* 33 1888–1897. 10.1111/j.1365-3040.2010.02189.x 20545883

[B62] Harrison M. J. van Buuren M. L. (1995). A phosphate transporter from the mycorrhizal fungus *Glomus versiforme*. *Nature* 378 626–629. 10.1038/378626a0 8524398

[B63] Hause B. Fester T. (2005). Molecular and cell biology of arbuscular mycorrhizal symbiosis. *Planta* 221 184–196. 10.1007/s00425-004-1436-x 15871030

[B64] Hause B. Mrosk C. Isayenkov S. Strack D. (2007). Jasmonates in arbuscular mycorrhizal interactions. *Phytochemistry* 68 101–110. 10.1016/j.phytochem.2006.09.025 17097695

[B65] Helber N. Wippel K. Sauer N. Schaarschmidt S. Hause B. Requena N. (2011). A versatile monosaccharide transporter that operates in the arbuscular mycorrhizal fungus *Glomus* sp is crucial for the symbiotic relationship with plants. *Plant Cell* 23 3812–3823. 10.1105/tpc.111.089813 21972259 PMC3229151

[B66] Hepper C. (1982). Limited independant growth of a vesicular-arbuscular mycorhizal fungus in vitro. *New Phytol.* 93 537–542. 10.1111/j.1469-8137.1983.tb02704.x

[B67] Herrera-Medina M. J. Steinkellner S. Vierheilig H. Ocampo Bote J. A. García Garrido J. M. (2007). Abscisic acid determines arbuscule development and functionality in the tomato arbuscular mycorrhiza. *New Phytol.* 175 554–564. 10.1111/j.1469-8137.2007.02107.x 17635230

[B68] Hibbett D. S. Binder M. Bischoff J. F. Blackwell M. Cannon P. F. Eriksson O. E. (2007). A higher–level phylogenetic classification of the Fungi. *Mycol. Res.* 111 509–547. 10.1016/j.mycres.2007.03.004 17572334

[B69] Hooker W. J. (1981). *Compendium of Potato Diseases.* Saint Paul, MN: American Phytopathological Society.

[B70] Ijdo M. Cranenbrouck S. Declerck S. (2011). Methods for large–scale production of AM fungi: past, present, and future. *Mycorrhiza* 21 1–16. 10.1007/s00572-010-0337-z 20803040

[B71] Javot H. Pumplin N. Harrison M. J. (2007). Phosphate in the arbuscular mycorrhizal symbiosis: transport properties and regulatory roles. *Plant Cell Environ.* 30 310–322. 10.1111/j.1365-3040.2006.01617.x 17263776

[B72] Jeffries P. Gianinazzi S. Perotto S. (2003). The contribution of arbuscular mycorrhizal fungi in sustainable maintenance of plant health and soil fertility. *Biol. Fertil. Soils* 37 1–16.

[B73] Juge C. Champagne A. Coughlan A. P. Juge N. Parrott L. Piché Y. (2009). Quantifying the growth of arbuscular mycorrhizal fungi: usefulness of the fractal dimension. *Botany* 87 387–400. 10.1139/B09-006

[B74] Juge C. Samson J. Bastien C. Vierheilig H. Coughlan A. Piché Y. (2002). Breaking dormancy in spores of the arbuscular mycorrhizal fungus *Glomus intraradices*: a critical cold–storage period. *Mycorrhiza* 12 37–42. 10.1007/s00572-001-0151-8 11968945

[B75] Karandashov V. Nagy R. Wegmüller S. Amrhein N. Bucher M. (2004). Evolutionary conservation of a phosphate transporter in the arbuscular mycorrhizal symbiosis. *Proc. Natl. Acad. Sci. U.S.A.* 101 6285–6290. 10.1073/pnas.0306074101 15075387 PMC395961

[B76] Karimi A. Khodaverdiloo H. Sepehri M. Sadaghiani M. R. (2011). Arbuscular mycorrhizal fungi and heavy metal contaminated soils. *Afr. J. Microbiol. Res.* 5 1571–1576.

[B77] Kiiskinen M. Korhonen M. Kangasja Èrvi J. (1997). Isolation and characterization of cDNA for a plant mitochondrial phosphate translocator (Mpt1). Ozone stress induces Mpt1 mRNA accumulation in birch (*Betula pendula* Roth). *Plant Mol. Biol.* 35 271–279. 10.1023/A:1005868715571 9349251

[B78] Kobae Y. Hata S. (2010). Dynamics of periarbuscular membranes visualized with a fluorescent phosphate transporter in arbuscular mycorrhizal roots of rice. *Plant Cell Physiol.* 51 341–353. 10.1093/pcp/pcq013 20097910

[B79] Kobae Y. Tamura Y. Takai S. Banba M. Hata S. (2010). Localized expression of arbuscular mycorrhiza-inducible ammonium transporters in soybean. *Plant Cell Physiol.* 51 1411–1415. 10.1093/pcp/pcq099 20627949

[B80] Koide R. T. (1985). The nature of growth depressions in sunflower caused by vesicular– arbuscular mycorrhizal infection. *New Phytol.* 99 445–462. 10.1111/j.1469-8137.1985.tb03672.x

[B81] Koske R. E. (1981). Gigaspora gigantea: obervations on spore germination of a vesicular-arbuscular mycorrhizal fungus. *Mycologia* 730 288–300. 10.2307/3759650

[B82] Krajinski F. Courty P.-E. Sieh D. Franken P. Zhang H. Bucher M. (2014). The H+-ATPase HA1 of *Medicago truncatula* is essential for phosphate transport and plant growth during arbuscular mycorrhizal symbiosis. *Plant Cell* 26 1808–1817. 10.1105/tpc.113.120436 24781114 PMC4036587

[B83] Lammers P. J. Jun J. Abubaker J. Arreola R. Gopalan A. Bago B. (2001). The glyoxylate cycle in an arbuscular mycorrhizal fungus. Carbon flux and gene expression. *Plant Physiol.* 127 1287–1298. 10.1104/pp.010375 11706207 PMC129296

[B84] Leggewie G. Willmitzer L. Riesmeier J. W. (1997). Two cDNAs from potato are able to complement a phosphate uptake-deficient yeast mutant: identification of phosphate transporters from higher plants. *Plant Cell* 9 381–392. 10.1105/tpc.9.3.381 9090882 PMC156925

[B85] Linkies A. Leubner-Metzger G. (2012). Beyond gibberellins and abscisic acid: how ethylene and jasmonates control seed germination. *Plant Cell Rep.* 31 253–270. 10.1007/s00299-011-1180-1 22044964

[B86] Liu Z. Li Y. Wang J. He X. Tian C. (2015). Different respiration metabolism between mycorrhizal and non-mycorrhizal rice under low-temperature stress: a cry for help from the host. *J. Agric. Sci.* 153 602–614. 10.1017/S0021859614000434

[B87] Lynch J. P. (2007). Roots of the second green revolution. *Austr. J. Bot.* 55 493–512. 10.1071/BT06118

[B88] Lynch T. Erikson B. J. Finkelstein R. R. (2012). Direct interactions of ABA–insensitive (ABI) clade protein phosphatase (PP)2Cs with calcium–dependent protein kinases and ABA response element–binding bZIPs may contribute to turning off ABA response. *Plant Mol. Biol.* 80 647–658. 10.1007/s11103-012-9973-3 23007729

[B89] Malusá E. Sas-Paszt L. Ciesielska J. (2012). Technologies for beneficial microorganisms inocula used as biofertilizers. *Sci. World J.* 2012:491206 10.1100/2012/491206 PMC3324119 22547984

[B90] Martin-Rodriguez J. León-Morcillo R. Vierheilig H. Ocampo J. A. Ludwig-Müller J. García-Garrido J. M. (2011). Ethylene–dependent/ethylene independent ABA regulation of tomato plants colonized by arbuscular mycorrhiza fungi. *New Phytol.* 190 193–205. 10.1111/j.1469-8137.2010.03610.x 21232061

[B91] Martin-Rodriguez J. A. Leon-Morcillo R. Vierheilig H. Ocampo J. A. Ludwig-Müller J. Garrido J. M. (2010). Mycorrhization of the notabilis and sitiens tomato mutants in relation to abscisic acid and ethylene content. *J. Plant Physiol.* 167 606–613. 10.1016/j.jplph.2009.11.014 20079554

[B92] McArthur D. A. Knowles N. R. (1992). Resistance responses of potato to vesicular–arbuscular mycorrhizal fungi under varying abiotic phosphorus levels. *Plant Physiol.* 100 341–351. 10.1104/pp.100.1.341 16652967 PMC1075557

[B93] Medina A. Jakobsen I. Vassilev N. Azcón R. Larsen J. (2007). Fermentation of sugar beet waste by *Aspergillus niger* facilitates growth and P uptake of external mycelium of mixed populations of arbuscular mycorrhizal fungi. *Soil Biol. Biochem.* 39 485–492. 10.1016/j.soilbio.2006.08.019

[B94] Medina A. Vassilev N. Azcón R. (2010). The interactive effect of an AM fungus and an organic amendment with regard to improving inoculum potential and the growth and nutrition of Trifolium repens in Cd-contaminated soils. *Appl. Soil Ecol.* 44 181–189. 10.1016/j.apsoil.2009.12.004

[B95] Mercy L. Svensson J. T. Lucic E. Cardoso H. G. Nogales A. Döring M. (2015). “AOX gene diversity in arbuscular mycorrhizal fungi (AMF) products – a special challenge”. Subchapter in Arnholdt-Schmit B. “From AOX diversity to functional marker development,” in *Alternative Respiratory Pathways in Higher Plants* eds Gupta K. J. Mur L. A. J. Neelwarne B. (Oxford: John Wiley & Sons, Inc.).

[B96] Millar A. H. Whelan J. Soole K. L. Day D. A. (2011). Organization and regulation of mitochondrial respiration in plants. *Annu. Rev. Plant Biol.* 62 79–104. 10.1146/annurev-arplant-042110-103857 21332361

[B97] Miller S. P. Bever J. D. (1999). Distribution of arbuscular mycorrhizal fungi in stands of the wetland grass *Panicum hemitomon* along a wide hydrologic gradient. *Oecologia* 119 586–592. 10.1007/s004420050823 28307718

[B98] Miller S. P. Sharitz R. R. (2000). Manipulation of flooding and arbuscular mycorrhiza formation influences growth and nutrition of two semiaquatic grass species. *Func. Ecol.* 14 738–748. 10.1046/j.1365-2435.2000.00481.x

[B99] Møller I. M. Berczi A. Van der Plas L. H. W. Lambers H. (1988). Measurement of the activity and capacity of the alternative pathway in intact plant–tissues: identification of problems and possible solutions. *Physiol. Plant.* 72 642–649. 10.1111/j.1399-3054.1988.tb09176.x

[B100] Mosse B. (1959). The regular germination of resting spores and some observations on the growth requirements of an *Endogone* sp causing vesicular-arbuscular mycorrhiza. *Trans. Br. Mycorrhizal Soc.* 42 273–286. 10.1016/S0007-1536(56)80033-8

[B101] Mukerji K. G. Manoharachary C. Chamola B. P. (2002). *Techniques in Mycorrhizal Studies.* London: Kluwer Academic Publishers 559 10.1007/978-94-017-3209-3

[B102] Murashige T. Skoog F. (1962). A revised medium for rapid growth and bioassays with tobacco tissue cultures. *Physiol. Plant.* 15 473–497. 10.1111/j.1399-3054.1962.tb08052.x

[B103] Nagy R. Drissner D. Amrhein N. Jakobsen I. Bucher M. (2009). Mycorrhizal phosphate uptake pathway in tomato is phosphorus-repressible and transcriptionally regulated. *New Phytol.* 181 950–959. 10.1111/j.1469-8137.2008.02721.x 19140941

[B104] Nagy R. Karandashov V. Chague V. Kalinkevich K. Tamasloukht M. Xu G. (2005). The characterization of novel mycorrhiza-specific phosphate transporters from *Lycopersicon esculentum* and *Solanum tuberosum* uncovers functional redundancy in symbiotic phosphate transport in solanaceous species. *Plant J.* 42 236–250. 10.1111/j.1365-313X.2005.02364.x 15807785

[B105] Nagy R. Vasconcelos M. J. Zhao S. McElver J. Bruce W. Amrhein N. (2006). Differential regulation of five Pht1 phosphate transporters from maize (*Zea mays* L.). *Plant Biol.* 8 186–197. 10.1055/s-2005-873052 16547863

[B106] Nogales A. Nobre T. Valadas V. Ragonezi C. Döring M. Polidoros A. (2016). Can functional hologenomics aid tackling current challenges in plant breeding? *Br. Funct. Genomics* 15 288–297. 10.1093/bfgp/elv030 26293603

[B107] Pfaffl M. W. (2001). A new mathematical model for relative quantification in real-time RT-PCR. *Nucleic Acids Res.* 29 2002–2007. 10.1093/nar/29.9.e45 PMC55695 11328886

[B108] Pfaffl M. W. Tichopad A. Prgomet C. Neuvians T. P. (2004). Determination of stable housekeeping genes, differentially regulated target genes and sample integrity: bestKeeper – Excel-based tool using pair-wise correlations. *Biotechnol. Lett.* 26 509–515. 10.1023/B:BILE.0000019559.84305.47 15127793

[B109] Pfanz H. Vodnik D. Wittmann C. Aschan G. Raschi A. (2004). “Plants and geothermal CO2 exhalations. Survival and adaptation to a high CO2 environment,” in *Progress in Botany 65* eds Esser K. Lüttge U. Kadereit J. W. Beyschlag W. (Berlin: Springer–Verlag) 499–538.

[B110] Pfeffer P. E. Douds D. D. Bécard G. Shachar–Hill Y. (1999). Carbon uptake and the metabolism and transport of lipids in an arbuscular mycorrhiza. *Plant Physiol.* 120 587–598. 10.1104/pp.120.2.587 10364411 PMC59298

[B111] Pinson B. Merle M. Franconi J.-M. Daignan-Fornier B. (2004). Low affinity orthophosphate carriers regulate *PHO* gene expression independently of internal orthophosphate concentration in *Saccharomyces cerevisiae*. *J. Biol. Chem.* 279 35273–35280. 10.1074/jbc.M405398200 15194704

[B112] Plaxton W. Tran H. (2011). Metabolic adaptations of phosphate-starved plants. *Plant Physiol.* 156 1006–1015. 10.1104/pp.111.175281 21562330 PMC3135920

[B113] Plenchette C. Fortin J. A. Furlan V. (1983). Growth responses of several plant species to mycorrhizae in a soil of moderate P–fertility. II. Soil fumigation induced stunting of plants corrected by reinoculation of the wild endomycorrhiza flora. *Plant Soil* 70 211–217. 10.1007/BF02374781

[B114] Porras-Soriano A. Soriano-Martín M. L. Porras-Piedra A. Azcón R. (2009). Arbuscular mycorrhizal fungi increased growth, nutrient uptake and tolerance to salinity in olive trees under nursery conditions. *J. Plant Physiol.* 166 1350–1359. 10.1016/j.jplph.2009.02.010 19342122

[B115] Pozo M. J. Cordier C. Dumas-Gaudot E. Gianinazzi S. Barea J. M. Azcón-Aguilar C. (2002). Localized vs systemic effect of arbuscular mycorrhizal fungi on defence responses to *Phytophthora* infection in tomato plants. *J. Exp. Bot.* 53 525–534. 10.1093/jexbot/53.368.525 11847251

[B116] Pozo M. J. Verhage A. Garcıa-Andrade J. García J. M. Azcón-Aguilar C. (2009). “Priming plant defence against pathogens by arbuscular mycorrhizal fungi,” in *Mycorrhizas – Functional Processes and Ecological Impact* Vol. 9 eds Azcón-Aguilar C. Barea J. M. Gianinazzi S. Gianinazzi-Pearson V. (Berlin: Springer) 123–136.

[B117] Quarles W. (1999). Plant disease biocontrol and VAM fungi. *IPM Pract.* 21 1–9. 10.2174/1874120701509010301

[B118] Rausch C. Daram P. Brunner S. Jansa J. Laloi M. Leggewie G. (2001). A phosphate transporter expressed in arbuscule-containing cells in potato. *Nature* 414 462–466. 10.1038/35106601 11719809

[B119] Requena N. (2005). Measuring quality of service: phosphate “a la carte” by arbuscular mycorrhizal fungi. *New Phytol.* 168 268–271. 10.1111/j.1469-8137.2005.01563.x 16219066

[B120] Rillig M. C. Steinberg P. D. (2002). Glomalin production by an arbuscular mycorrhizal fungus: a mechanism of habitat modification? *Soil Biol. Biochem.* 34 1371–1374. 10.1016/s0038-0717(02)00060-3

[B121] Rizzuto R. Sandonà D. Brini M. Capaldi R. A. Bisson R. (1991). The most conserved nuclear-encoded polypeptide of cytochrome c oxidase is the putative zinc-binding subunit: primary structure of subunit V from the slime mold *Dictyostelium discoideum*. *Biochim. Biophys. Acta* 1129 100–104. 10.1016/0167-4781(91)90220-G 1661610

[B122] Ronsheim M. L. (2012). The effect of mycorrhizae on plant growth and reproduction varies with soil phosphorus and developmental stage. *Am. Midl. Nat.* 167 28–39. 10.1674/0003-0031-167.1.28

[B123] Rook F. Hadingham S. A. Li Y. Bevan M. W. (2006). Sugar and ABA response pathways and the control of gene expression. *Plant Cell Environ.* 29 426–434. 10.1111/j.1365-3040.2005.01477.x 17080596

[B124] Ruiz O. H. Gonzalez A. Almeida A. J. Tamayo D. Garcia A. M. Restrepo A. (2011). Alternative oxidase mediates pathogen resistance in *Paracoccidioides brasiliensis* infection. *PLoS Negl. Trop. Dis.* 5:e1353 10.1371/journal.pntd.0001353 PMC3201906 22039556

[B125] Ruzicka D. R. Hausmann N. T. Barrios-Masias F. H. Jackson L. E. Schachtman D. P. (2012). Transcriptomic and metabolic responses of mycorrhizal roots to nitrogen patches under field conditions. *Plant Soil* 350 145–162. 10.1007/s11104-011-0890-z

[B126] Saif S. R. (1981). The influence of soil aeration on the efficiency of vesicular–arbuscular mycorrhizae. 1. Effect of soil oxygen on the growth and mineral uptake of *Eupatorium odoratum* L. inoculated with *Glomus macrocarpus*. *New Phytol.* 88 649–659. 10.1111/j.1469-8137.1981.tb01741.x

[B127] Saini G. R. (1976). Relationship between potato yield and oxygen diffusion rate of subsoil. *Agron. J.* 68 823–825. 10.2134/agronj1976.00021962006800050036x

[B128] Sakano K. (2001). Metabolic regulation of pH in plant cells: role of cytoplasmic pH in defense reaction and secondary metabolism. *Int. Rev. Cytol.* 206 1–44. 10.1016/S0074-7696(01)06018-1 11407758

[B129] Salcedo-Hernandez R. Escamilla E. Ruiz-Herrera J. (1994). Organization and regulation of the mitochondrial oxidative pathway in *Mucor rouxii*. *Microbiology* 140 399–407. 10.1099/13500872-140-2-399

[B130] Sanders F. E. (1975). “The effect of foliar–applied phosphate on the mycorrhizal infection of onion roots,” in *Endomycorrhizas* eds Sanders F. E. Mosse B. Tinker P. B. (London: Academic Press) 261–276.

[B131] Schreiner R. P. (2010). Foliar sprays containing phosphorus (P) have minimal impact on ‘Pinot noir’ growth and P status, mycorrhizal colonization, and fruit quality. *HortScience* 45 815–820.

[B132] Schreiner R. P. Linderman R. G. (2005). Mycorrhizal colonization in dryland vineyards of the Willamette Valley, Oregon. *Small Fruits Rev.* 4 41–55. 10.1300/J301v04n03_04

[B133] Sengottaiyan P. Ruiz-Pavon L. Persson B. L. (2013). Functional expression, purification and reconstitution of the recombinant phosphate transporter Pho89 of *Saccharomyces cerevisiae*. *FEBS J.* 280 965–975. 10.1111/febs.12090 23216645 PMC3633241

[B134] Sesay A. Stewart C. R. Shibles R. M. (1986). Effects of KCN and salicylhydroxamic acid on respiration of soybean leaves at different ages. *Plant Physiol.* 82 443–447. 10.1104/pp.82.2.443 16665048 PMC1056137

[B135] Sieger S. M. Kristensen B. K. Robson C. A. Amirsadeghi S. Eng E. W. Y. Abdel-Mesih A. (2005). The role of alternative oxidase in modulating carbon use efficiency and growth during macronutrient stress in tobacco cells. *J. Exp. Bot.* 56 1499–1515. 10.1093/jxb/eri146 15824074

[B136] Simons B. H. Millenaar F. F. Mulder L. Van Loon L. C. Lambers H. (1999). Enhanced expression and activation of the alternative oxidase during infection of Arabidopsis with *Pseudomonas syringae* pv tomato. *Plant Physiol.* 120 529–538. 10.1104/pp.120.2.529 10364404 PMC59291

[B137] Singh S. (2001). Role of mycorrhiza in tree plantings in the field, Part II: field inoculation, fungal succession, and effect of climatic and edaphic factors. *Mycorrhiza News* 12 2–12.

[B138] Siquiera J. O. Hubbell D. H. (1986). Effect of organic substrates on germination and germ tube growth of vesicular-arbuscular mycorrhizal fungus spores in vitro. *Pesqui. Agropecu. Bras.* 21 523–527.

[B139] Smith F. A. Grace E. J. Smith S. E. (2009). More than a carbon economy: nutrient trade and ecological sustainability in facultative arbuscular mycorrhizal symbioses. *New Phytol.* 182 347–358. 10.1111/j.1469-8137.2008.02753.x 19207688

[B140] Smith F. A. Smith S. E. (2011a). What is the significance of the arbuscular mycorrhizal colonization of many economically important crop plants? *Plant Soil* 348 63–79. 10.1007/s11104-011-0865-0

[B141] Smith S. E. Jakobsen I. Grønlund M. Smith F. A. (2011). Roles of arbuscular mycorrhizas in plant phosphorus nutrition: interactions between pathways of phosphorus uptake in arbuscular mycorrhizal roots have important implications for understanding and manipulating plant phosphorus acquisition. *Plant Physiol.* 156 1050–1057. 10.1104/pp.111.174581 21467213 PMC3135927

[B142] Smith S. E. Read D. J. (2008). *Mycorrhizal Symbiosis.* Cambridge: Academic Press.

[B143] Smith S. E. Smith F. A. (2011b). Roles of arbuscular mycorrhizas in plant nutrition and growth: new paradigms from cellular to ecosystem scales. *Annu. Rev. Plant Biol.* 62 227–250. 10.1146/annurev-arplant-042110-103846 21391813

[B144] Spreen Brouwer K. van Valen T. Day D. A. Lambers H. (1986). Hydroxamate–stimulated O2 uptake in roots of *Pisum sativum* and *Zea mays*, mediated by a peroxidase. *Plant Physiol.* 82 236–240. 10.1104/pp.82.1.236 16664999 PMC1056096

[B145] St-Arnaud M. Hamel C. Vimard B. Caron M. Fortin J. A. (1996). Enhanced hyphal growth and spore production of the arbuscular mycorrhizal fungus *Glomus* intraradices in an in vitro system in absence of host roots. *Mycol. Res.* 100 328–332. 10.1016/S0953-7562(96)80164-X

[B146] Tamasloukht M. Séjalon-Delmas N. Kluever A. Jauneau A. Roux C. Bécard G. (2003). Root factors induce mitochondrial related gene expression and fungal respiration during the developmental switch from asymbiosis to presymbiosis in the arbuscular mycorrhizal fungus Gigaspora rosea. *Plant Physiol.* 131 1468–1478. 10.1104/pp.012898 12644696 PMC166906

[B147] Tamura K. Stecher G. Peterson D. Filipski A. Kumar S. (2013). MEGA6: molecular evolutionary genetics analysis version 6.0. *Mol Biol Evol.* 30 2725–2729. 10.1093/molbev/mst197 24132122 PMC3840312

[B148] Thomazella D. P. Teixeira P. J. Oliveira H. C. Saviani E. E. Rincones J. Toni I. M. (2012). The hemibiotrophic cacao pathogen *Moniliophthora perniciosa* depends on a mitochondrial alternative oxidase for biotrophic development. *New Phytol.* 194 1025–1034. 10.1111/j.1469-8137.2012.04119.x 22443281 PMC3415677

[B149] Tisserant E. Kohler A. Dozolme-Seddas P. Balestrini R. Benabdellah K. Colard A. (2012). The transcriptome of the arbuscular mycorrhizal fungus *Glomus* intraradices (DAOM197198) reveals functional tradeoffs in an obligate symbiont. *New Phytol.* 193 755–769. 10.1111/j.1469-8137.2011.03948.x 22092242

[B150] Tisserant E. Malbreil M. Kuo A. Kohler A. Symeonidi A. Balestrini R. (2013). Genome of an arbuscular mycorrhizal fungus provides insight into the oldest plant symbiosis. *Proc. Natl. Acad. Sci. U.S.A.* 110 20117–20122. 10.1073/pnas.1313452110 24277808 PMC3864322

[B151] Trépanier M. Bécard G. Moutoglis P. Willemot C. Gagné S. Avis T. J. (2005). Dependence of arbuscular–mycorrhizal fungi on their plant host for palmitic acid synthesis. *Appl. Environ. Microbiol.* 71 5341–5347. 10.1128/AEM.71.9.5341-5347.2005 16151123 PMC1214663

[B152] Trouvelot A. Kough J. L. Gianinazzi-Pearson V. (1986). “Mesure du taux de mycorhization ayant une signification fonctionnelle,” in *Aspects Physiologiques et Génétiques des Mycorhizes* eds Gianinazzi-Pearson V. Gianinazzi S. (Paris: INRA Press) 217–222.

[B153] Umbach A. L. Ng V. S. Siedow J. N. (2006). Regulation of plant alternative oxidase activity: a tale of two cysteines. *Biochim. Biophys. Acta* 1757 135–142. 10.1016/j.bbabio.2005.12.005 16457775

[B154] Umbach A. L. Siedow J. N. (2000). The cyanide-resistant alternative oxidases from the fungi *Pichia stipitis* and *Neurospora crassa* are monomeric and lack regulatory features of the plant enzyme. *Arch. Biochem. Biophys.* 378 234–245. 10.1006/abbi.2000.1834 10860541

[B155] Uribe D. Khachatourians G. G. (2008). Identification and characterization of an alternative oxidase in the entomopathogenic fungus *Metarhizium anisopliae*. *Can. J. Microbiol.* 54 119–127. 10.1139/w07-127 18388981

[B156] Ury H. K. (1976). A comparison of four procedures for multiple comparisons among means (pairwise contrasts) for arbitrary sample sizes. *Technometrics* 18 89–97. 10.2307/1267921

[B157] van Aarle I. Cavagnaro T. R. Smith S. E. Smith F. A. Dickson S. (2005). Metabolic activity of *Glomus* intraradices in *Arum*- and Paris-type arbuscular mycorrhizal colonization. *New Phytol.* 166 611–618. 10.1111/j.1469-8137.2005.01340.x 15819923

[B158] van der Heijden M. G. A. Martin F. M. Selosse M.-A. Sanders I. R. (2015). Mycorrhizal ecology and evolution: the past, the present, and the future. *New Phytol.* 205 1406–1423. 10.1111/nph.13288 25639293

[B159] Vandesompele J. De Preter K. Pattyn F. Poppe B. Van Roy N. De Paepe A. (2002). Accurate normalization of real-time quantitative RT-PCR data by geometric averaging of multiple internal control genes. *Genome Biol.* 3 RESEARCH0034 10.1186/gb-2002-3-7-research0034 PMC126239 12184808

[B160] Vanlerberghe G. C. (2013). Alternative oxidase: a mitochondrial respiratory pathway to maintain metabolic and signaling homeostasis during abiotic and biotic stress in plants. *Int. J. Mol. Sci.* 14 6805–6847. 10.3390/ijms14046805 23531539 PMC3645666

[B161] Vierheilig H. Coughlan A. P. Wyss U. Piche Y. (1998). Ink and vinegar, a simple staining technique for arbuscular–mycorrhizal fungi. *Appl. Environ. Microbiol.* 64 5004–5007. 9835596 10.1128/aem.64.12.5004-5007.1998 PMC90956

[B162] Volkmar K. M. Woodbury W. (1989). Effects of soil temperature and depth on colonization and root and shoot growth of barley inoculated with vesicular–arbuscular mycorrhizae indigenous to Canadian prairie soil. *Can. J. Bot.* 67 1702–1707. 10.1139/b89-215

[B163] Vosátka M. Albrechtová J. Patten R. (2008). “The international market development for mycorrhizal technology,” in *Mycorrhiza* ed. Varma A. (Berlin: Springer) 419–438.

[B164] Walder F. Boller T. Wiemken A. Courty P. E. (2016). Regulation of plants’ phosphate uptake in common mycorrhizal networks: role of intraradical fungal phosphate transporters. *Plant Signal. Behav.* 11:e1131372 10.1080/15592324.2015.1131372 PMC4883902 26751110

[B165] Walder F. Brulé D. Koegel S. Wiemken A. Boller T. Courty P. E. (2015). Plant phosphorus acquisition in a common mycorrhizal network: regulation of phosphate transporter genes of the Pht1 family in sorghum and flax. *New Phytol.* 205 1632–1645. 10.1111/nph.13292 25615409

[B166] Walley F. L. Germida J. J. (1995). Estimating the viability of vesicular—arbuscular mycorrhizae fungal spores using tetrazolium salts as vital stains. *Mycologia* 87 273–279. 10.2307/3760914

[B167] Wang H. Huang J. Bi Y. (2010). Induction of alternative respiratory pathway involves nitric oxide, hydrogen peroxide and ethylene under salt stress. *Plant Signal. Behav.* 5 1636–1637. 10.4161/psb.5.12.13775 21139431 PMC3115120

[B168] Wang Y. Tang S. Jin H. (2015). Effect of glucose, root exudates and N forms in mycorrhizal symbiosis using *Rhizophagus intraradices*. *J. Soil Sci. Plant Nutr.* 15 726–736. 10.4067/s0718-95162015005000049

[B169] Welchen E. Chan R. L. Gonzalez D. H. (2002). Metabolic regulation of genes encoding cytochrome c and cytochrome c oxidase subunit Vb in *Arabidopsis*. *Plant Cell Environ.* 25 1605–1615. 10.1046/j.1365-3040.2002.00940.x

[B170] Welchen E. Hildebrandt T. M. Lewejohann D. Gonzalez D. H. Braun H.-P. (2012). Lack of cytochrome c in Arabidopsis decreases stability of Complex IV and modifies redox metabolism without affecting Complexes I and III. *Biochim. Biophys. Acta* 1817 990–1001. 10.1016/j.bbabio.2012.04.008 22551905

[B171] Wewer V. Brands M. Dörmann P. (2014). Fatty acid synthesis and lipid metabolism in the obligate biotrophic fungus *Rhizophagus irregularis* during mycorrhization of *Lotus japonicus*. *Plant J.* 79 398–412. 10.1111/tpj.12566 24888347

[B172] Whipps J. M. (2004). Prospects and limitations for mycorrhizas in biocontrol of root pathogens. *Can. J. Bot.* 82 1198–1227. 10.1139/b04-082

[B173] White C. N. Rivin C. J. (2000). Gibberellins and seed development in maize. II. Gibberellin synthesis inhibition enhances abscisic acid signaling in cultured embryos. *Plant Physiol.* 122 1089–1098. 10.1104/pp.122.4.1089 10759504 PMC58943

[B174] Wind J. J. Peviani A. Snel B. Hanson J. Smeekens S. C. (2012). ABI4: versatile activator and repressor. *Trends Plant Sci.* 19 1360–1385. 10.1016/j.tplants.2012.10.004 23182343

[B175] Xu F. Yuan S. Zhang D. W. Lv X. Lin H. H. (2012). The role of alternative oxidase in tomato fruit ripening and its regulatory interaction with ethylene. *J. Exp. Bot.* 63 5705–5716. 10.1093/jxb/ers226 22915749 PMC3444281

[B176] Xu T. Yao F. Liang W. S. Li Y. H. Li D. R. Wang H. (2012). Involvement of alternative oxidase in the regulation of growth, development, and resistance to oxidative stress of *Sclerotinia sclerotiorum*. *J. Microbiol.* 50 594–602. 10.1007/s12275-012-2015-7 22923107

[B177] Yang S.-Y. Grønlund M. Jakobsen I. Grotemeyer M. S. Rentsch D. Miyao A. (2012). Nonredundant regulation of rice arbuscular mycorrhizal symbiosis by two members of the PHOSPHATE TRANSPORTER1 gene family. *Plant Cell* 24 4236–4251. 10.1105/tpc.112.104901 23073651 PMC3517247

[B178] Zhu X. C. Song F. B. Liu S. Q. Liu T. D. (2011). Effects of arbuscular mycorrhizal fungus on photosynthesis and water status of maize under high temperature stress. *Plant Soil* 346 189–199. 10.1007/s11104-011-0809-8

[B179] Zhu X. C. Song F. B. Xu W. (2010). Arbuscular mycorrhizae improves low temperature stress in maize via alterations in host water status and photosynthesis. *Plant Soil* 331 129–137. 10.4161/psb.11498

[B180] Zsigmond L. Rigó G. Szarka A. Székely G. Ötvös K. Darula Z. (2008). Arabidopsis PPR40 connects abiotic stress responses to mitochondrial electron transport. *Plant Physiol.* 149 1721–1737. 10.1104/pp.107.111260 PMC2287346 18305213

[B181] Zsögön A. Lambais M. R. Benedito V. A. Figueira A. V. O. Peres L. E. P. (2008). Reduced arbuscular mycorrhizal colonization in tomato ethylene mutants. *Sci. Agric.* 65 259–267. 10.1590/S0103-90162008000300006

