# Supplementary material for: A Functional Approach towards Understanding the Role of the Mitochondrial Respiratory Chain in an Endomycorrhizal Symbiosis
Source: Front Plant Sci. 2017 Mar 30;8:417. doi: 10.3389/fpls.2017.00417 (PMC5371606; doi:10.3389/fpls.2017.00417)
Supplement: Supplementary file 1 [file Data_Sheet_1.PDF]

Supplementary Material

A functional approach of the mitochondrial respiratory chain in an endomycorrhizal symbiosis

Louis Mercy[1‡*], Eva Lucic-Mercy[1‡], Amaia Nogales[2, 3], Areg Poghosyan[1], Carolin Schneider[1] & Birgit Arnholdt-Schmitt[3]

[1]INOQ GmbH, Solkau 2, 29465 Schnega, Germany
[2] Current address: Linking Landscape, Environment, Agriculture and Food (LEAF)-Instituto Superior de Agronomia (ISA), University of Lisbon, 1349-017 Lisbon, Portugal
[3]EU Marie Curie Chair, ICAAM – Instituto de Ciências Agrárias e Ambientais Mediterrânicas, IIFA - Instituto de Investigação e Formação Avançada - Universidade de Évora, Núcleo da Mitra, Ap. 94, 7002-554 Évora, Portugal

[‡] These authors contributed equally to this work

*** Correspondance:**
Mercy Louis, INOQ GmbH, Solkau 2, 29465 Schnega, Germany
mercy@inoq.de

1 Supplementary Figures

1.1 Supplementary Figure S1

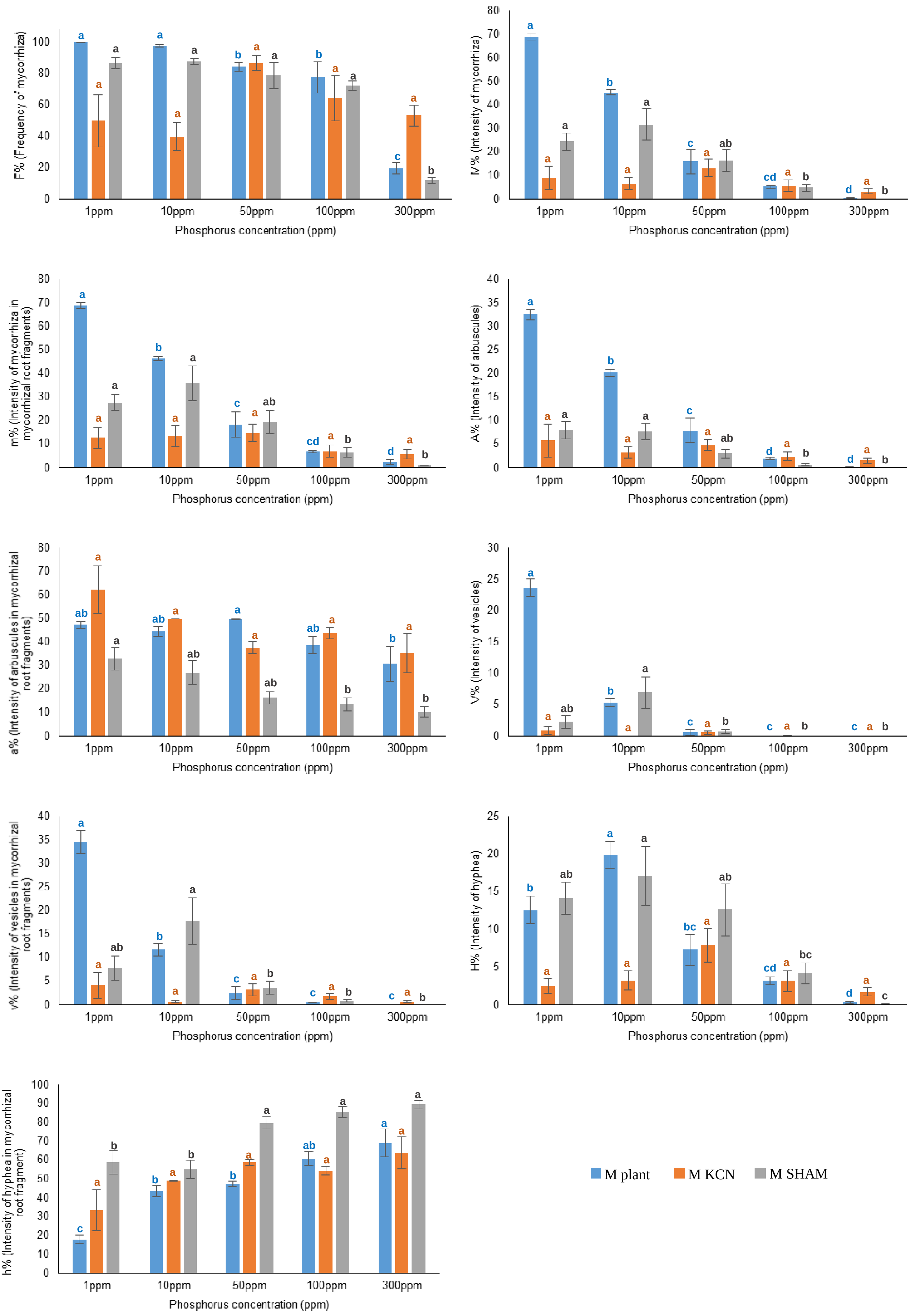
F% (Frequency of mycorrhiza)
M% (Intensity of mycorrhiza)
m% (Intensity of mycorrhiza in mycorrhizal root fragments)
A% (Intensity of arbuscules)
a% (Intensity of arbuscules in mycorrhizal root fragments)
V% (Intensity of vesicles)
v% (Intensity of vesicles in mycorrhizal root fragments)
H% (Intensity of hyphea)
h% (Intensity of hyphea in mycorrhizal root fragment)
1ppm
10ppm
50ppm
100ppm
300ppm
Phosphorus concentration (ppm)
M plant
M KCN
M SHAM

Figure S1. Mycorrhizal rate parameters of potato plants treated or not with respiratory chain inhibitors under 5 phosphorus concentrations. Parameters estimated according to Trouvelot *et al.* (1986) in potato roots inoculated with *R. irregulare*, harvested at 8 WAI. Plantlets were treated or not by SHAM (at 7 DAI – 0.1 mM) or by KCN (at 7 DAI – 0.1 mM). Values represent means ±SE (n= 3 repetitions). Differences between treatments were examined by a one-way analysis of variance (ANOVA) after arcsin transformation of values. Duncan's multiple range tests were performed to identify significant differences (P < 0.05, symbolized by letters) among P concentrations. Statistical data which identify significant differences between treatments along P concentrations are developed in Figure S1. Data analysis was performed with the SAS enterprise guide 4.1 (SAS Institute Inc., Cary, USA).

1.2 Supplementary Figure S2

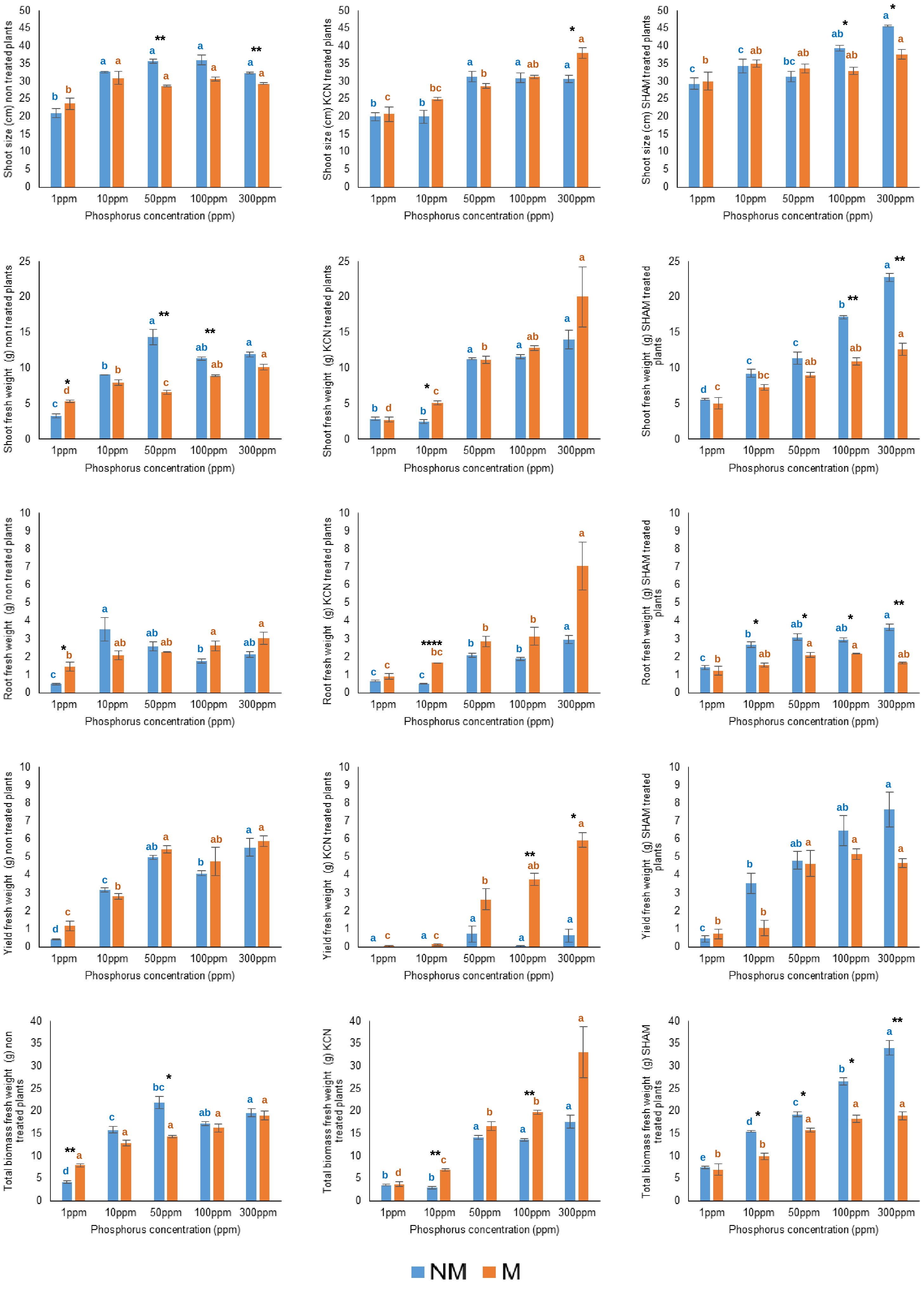

Figure S2. Plant growth parameters of potato plants inoculated with *R. irregulare* or not, treated with respiratory chain inhibitors or not under 5 phosphorus concentrations. Potato roots were harvested at 8 WAI. Plantlets were treated or not by SHAM (at 7 DAI – 0.1 mM) or by KCN (at 7 DAI – 0.1 mM). Values represent means (n=3) ± s.e. Differences between treatments were examined by a one-way analysis of variance (ANOVA) after a log transformation of values. Duncan's multiple range tests were performed to identify significant differences ($P < 0.05$, symbolized by letters) among P concentrations. Statistical data which identify significant differences between treatments along P concentrations are developed in Figure S2. Data analysis was performed with the SAS enterprise guide 4.1 (SAS Institute Inc., Cary, USA)

1.3 Supplementary Figure S3

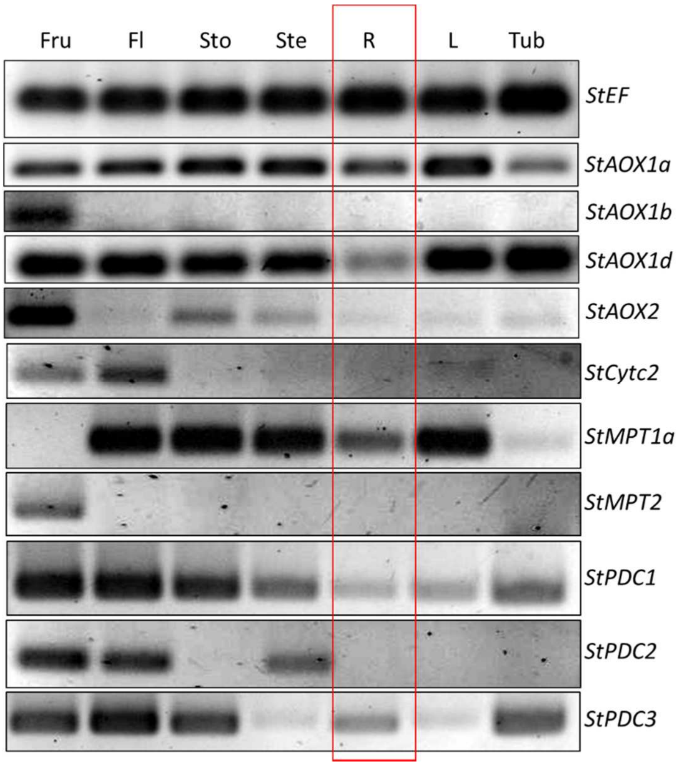

Figure S3. Semi quantitative RT-PCR assays on potato tissues. RT-PCR analyses were performed on potato tissue samples. 250ng of total RNA extracted from 100 mg of tissues conserved in RNAlater (Qiagen) using the RNeasy Plant Mini kit (Qiagen) were used for cDNA synthesis with an oligo(dT) primer (Promega) and reverse transcriptase (Masterscript^{TM} Kit, 5 Prime, Germany). Semi-quantitative RT-PCR analysis were performed with the following composition for each 25 µl assay: 5µl of 5X Green GoTaq® Reaction Buffer (Promega), 0,5µl of dNTP (10mM, Promega) 0,5µl of each forward and reverse primer (10µM), 1µl of cDNA template and 0,1µl of GoTaq® DNA Polymerase (Promega). All primers used are listed in Table S1. Fru: fruit, Fl: flower, Sto: stolon, Ste: stem, R: root, L: leaves, Tub: tuber.

1.4 Supplementary Figure S4

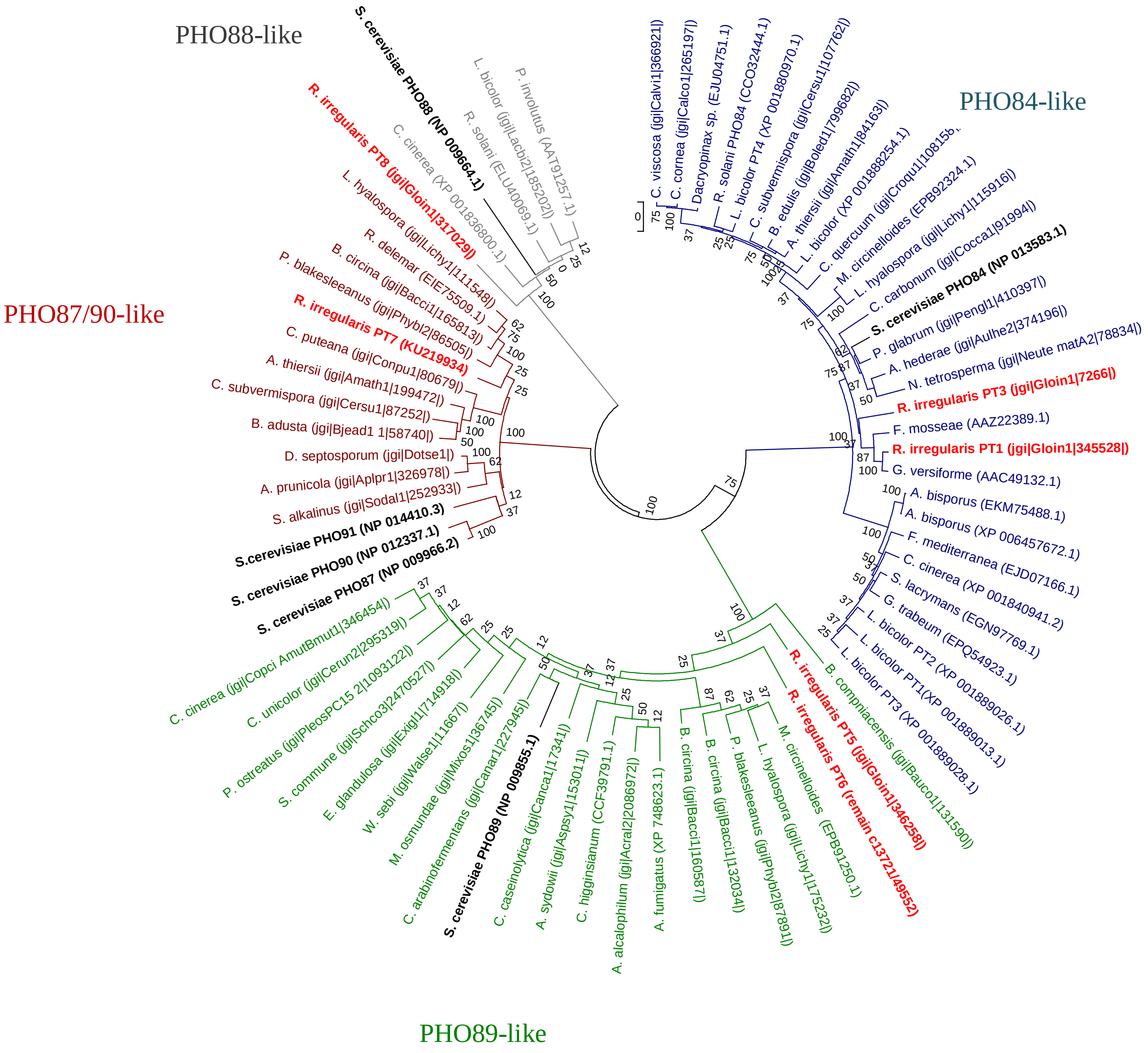

Figure S4. Neighbour-joining tree for fungal PHS family. Protein ID are indicated, either as GeneBank accessions, or as JGI fungal genome identifiants (indicated by jgi/), and for PT6, refers also to sequences Remain_c13721 and Remain_c49552 from *Rhizophagus irregularis* DB (http://mycor.nancy.inra.fr/IMGC/GlomusGenome/search3.html). Full-length amino acid sequences were aligned by CLUSTALW and imported into the Molecular Evolutionary Genetics Analysis (MEGA) package version 6 (Tamura *et al.*, 2013). Phylogenetic analyses were conducted using the neighborjoining (NJ) method implemented in MEGA with the pairwise deletion option for handling alignment gaps, and with the Poisson correction model for distance computation. Bootstrap tests were conducted using 1000 replicates.

1.5 Supplementary Figure S5

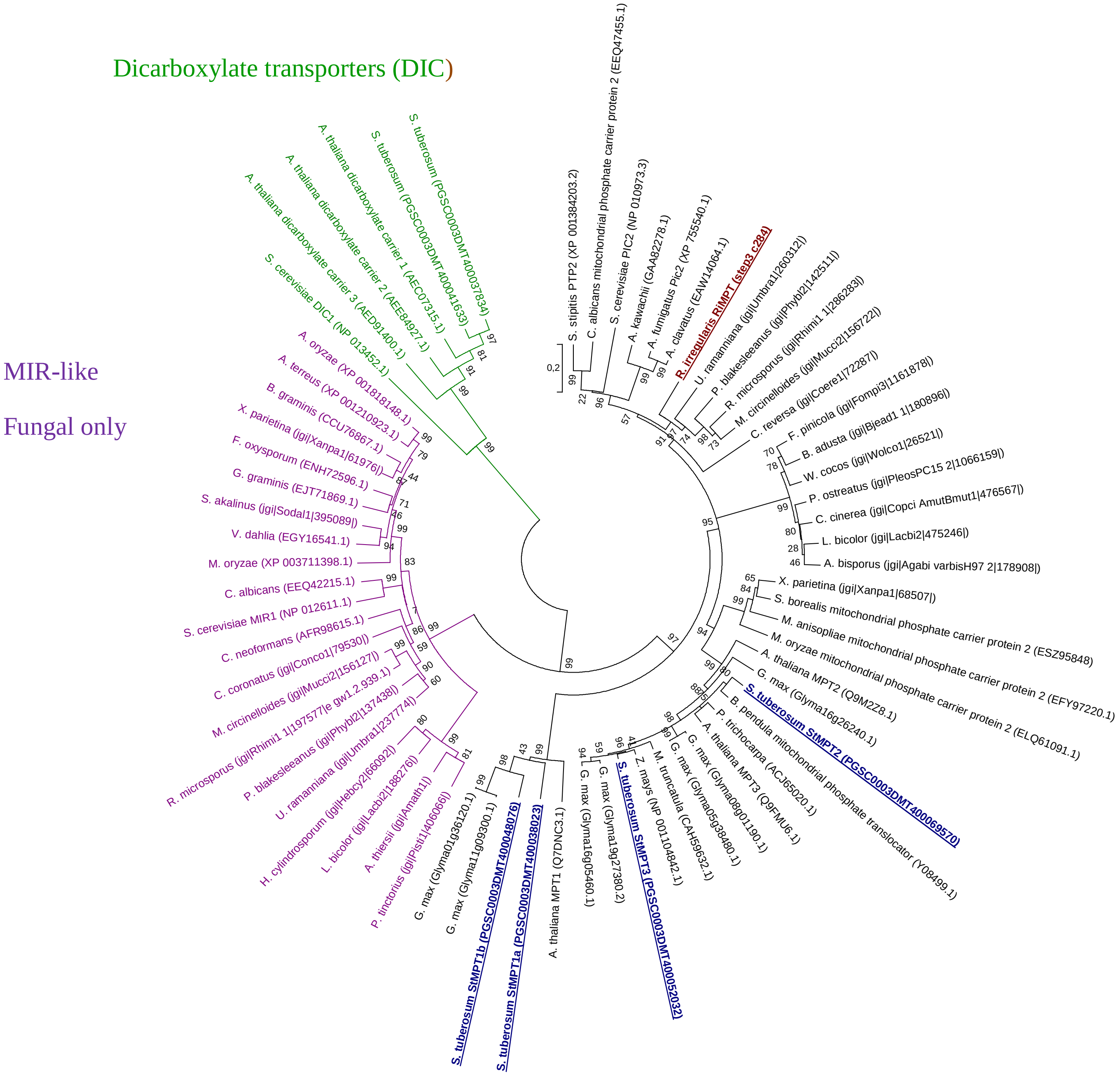

Figure S5. Neighbour-joining tree for for Pht3 mitochondrial P transporter family. Protein ID are indicated, either as GeneBank accessions, or as JGI fungal genome identifiants (indicated by jgi/). Full-length amino acid sequences were aligned by CLUSTALW and imported into the Molecular Evolutionary Genetics Analysis (MEGA) package version 6 (Tamura *et al.*, 2013). Phylogenetic analyses were conducted using the neighborjoining (NJ) method implemented in MEGA with the pairwise deletion option for handling alignment gaps, and with the Poisson correction model for distance computation. Bootstrap tests were conducted using 1000 replicates.

1.6 Supplementary Figure S6

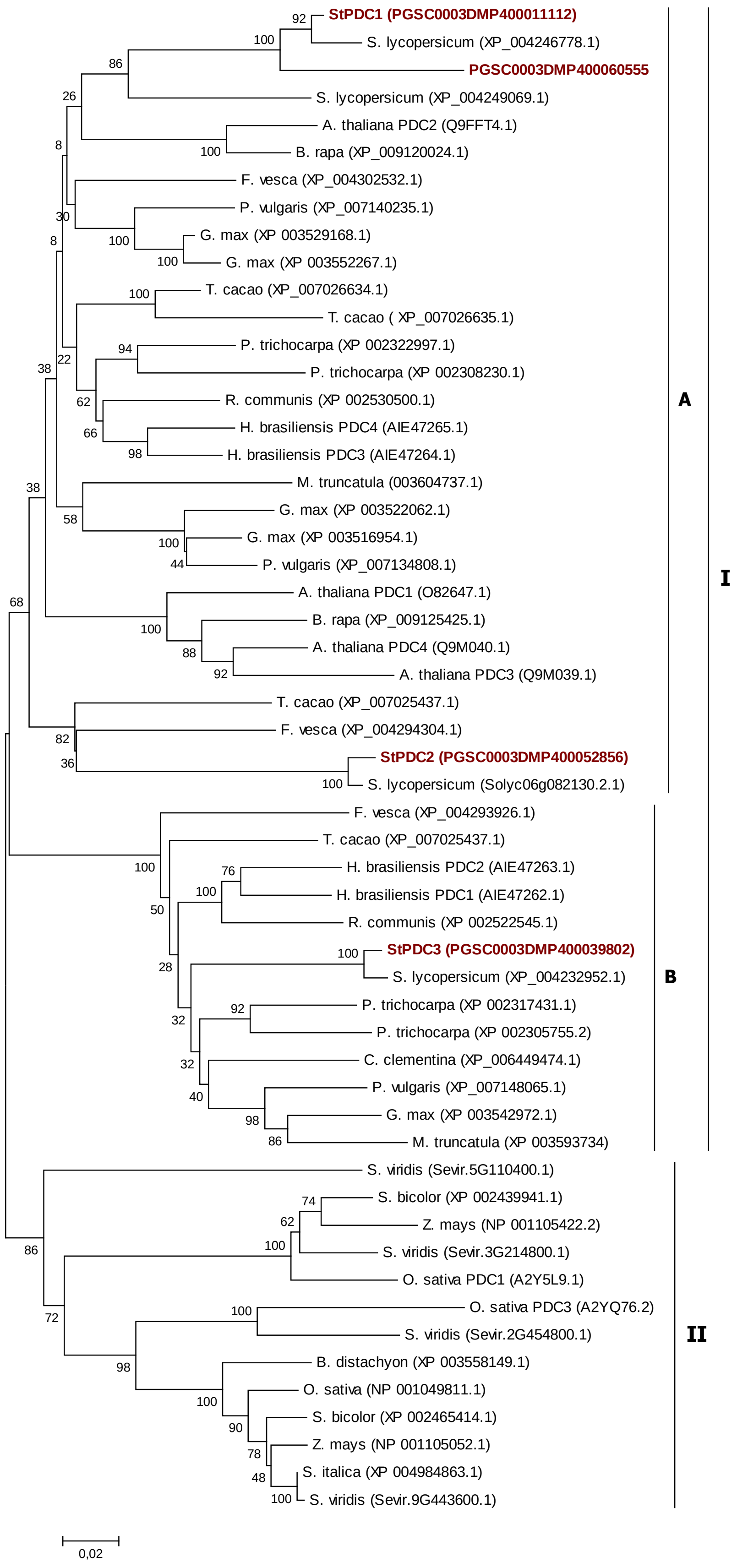

Figure S6. Neighbour-joining tree of plant Pyruvate Decarboxylase (PDC, EC: 4.1.1.1) family. Protein ID are indicated, either as GeneBank accessions, or as Phytozome identifiants (https://phytozome.jgi.doe.gov/pz/portal.html) for *S. viridis* and one *S. lycopersicum* sequence. Full-length amino acid sequences were aligned by CLUSTALW and imported into the Molecular Evolutionary Genetics Analysis (MEGA) package version 6 (Tamura *et al.*, 2013). Phylogenetic analyses were conducted using the neighborjoining (NJ) method implemented in MEGA with the pairwise deletion option for handling alignment gaps, and with the Poisson correction model for distance computation. Bootstrap tests were conducted using 1000 replicates.

1.7 Supplementary Figure S7

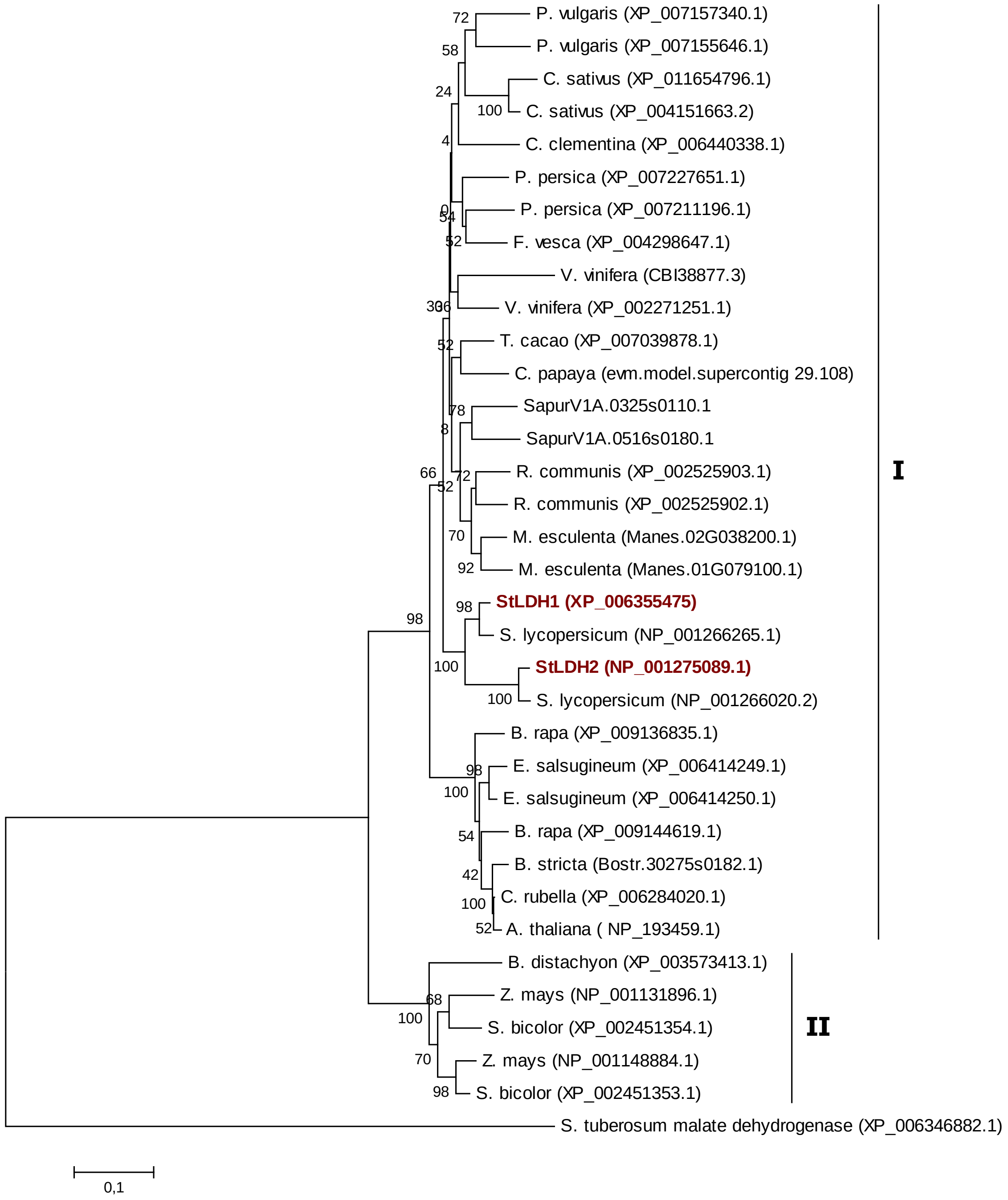

Figure S7. Neighbour-joining tree for the plant Lactate Dehydrogenase (LDH - EC 1.1.1.27) family. Protein ID are indicated, either as GeneBank accessions, or as Phytozome identifiants (https://phytozome.jgi.doe.gov/pz/portal.html) for *C. papaya, M. esculenta* and *B. stricta* sequences. Full-length amino acid sequences were aligned by CLUSTALW and imported into the Molecular Evolutionary Genetics Analysis (MEGA) package version 6 (Tamura *et al.*, 2013). Phylogenetic analyses were conducted using the neighborjoining (NJ) method implemented in MEGA with the pairwise deletion option for handling alignment gaps, and with the Poisson correction model for distance computation. Bootstrap tests were conducted using 1000 replicates. Malate dehydrogenase sequence was used as outgroup.

1.8 Supplementary Figure S8

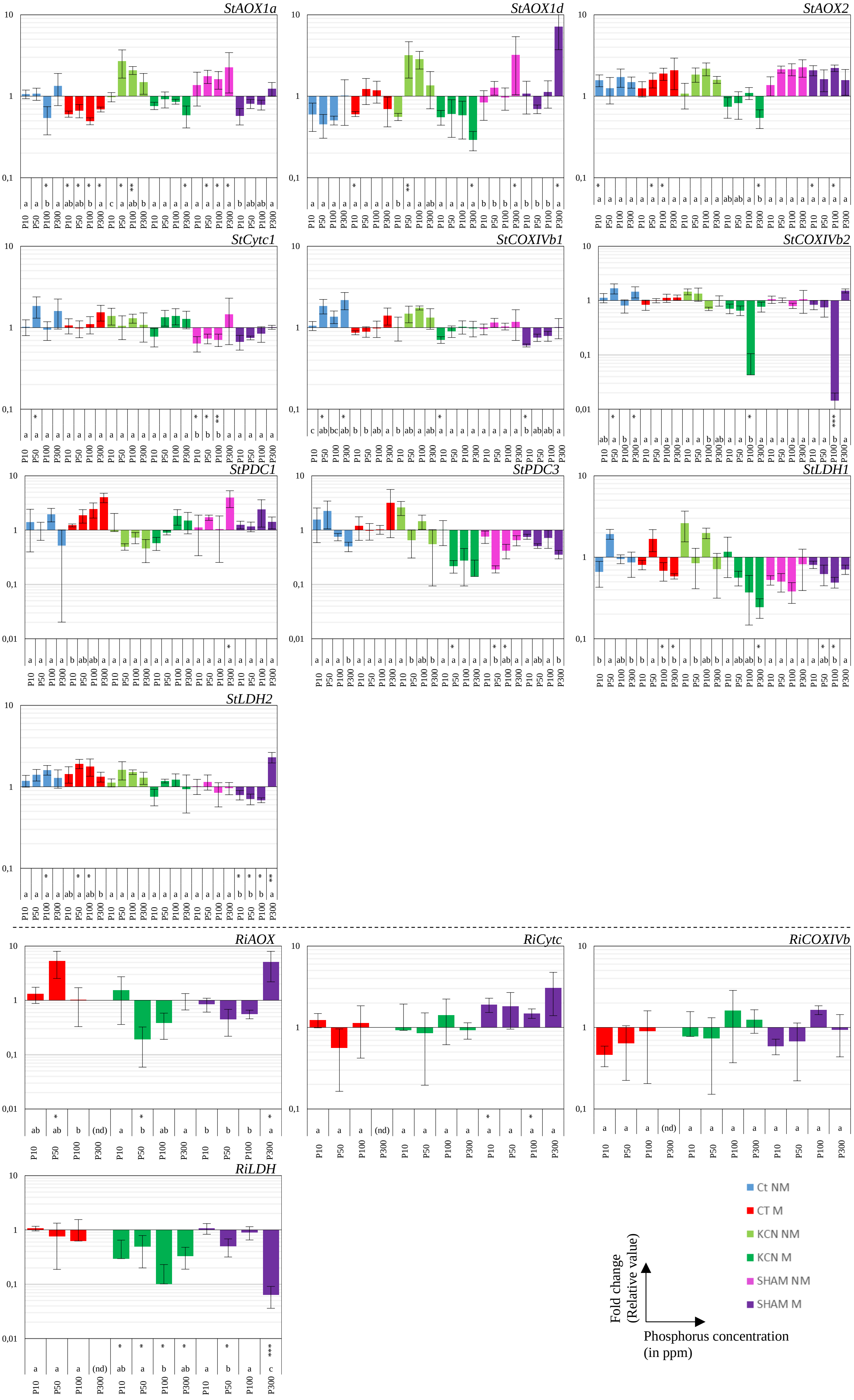

Figure S8. Effect of P concentration on the relative expression of genes involved in mitochondrial respiratory chain (3 *StAOX* isoforms, *StCytc1*, *StCOXVb1*, *StCOXVb2*, *RiAOX*, *RiCytc and RiCOXIVb*) and fermentation (2 *StPDC* and 2 *StLDH* isoforms and *RiLDH*) in potato root and in *R. irregulare*, following inoculation or not of AMF, treatment or not with two respiratory inhibitors through 5 phosphorus concentrations. Relative expressions are represented relative to P concentration (compared to 1 ppm P as reference respective to each treatment, root samples harvested after 8 WAI). Differences in the mean expression level (n=3), where each sample was analyzed in duplicate of target genes between treatments were examined by a one-way analysis of variance (ANOVA) after a log transformation of the normalized relative gene expression levels. Dunnett's test was conducted to identify significant differences (symbolized by stars placed above P concentrations in x-axis; NS: P>0.05, *: P<0.05, **: P<0.005, ***: P<0.0005) compared to 1 ppm P as reference respective to each treatment. Duncan's multiple range tests were performed to identify significant differences ($P < 0.05$, symbolized by letters indicated above graphs) among P concentrations (after standardization against specified control). Data analysis was performed with the SAS enterprise guide 4.1 (SAS Institute Inc., Cary, USA).

1.9 Supplementary Figure S9

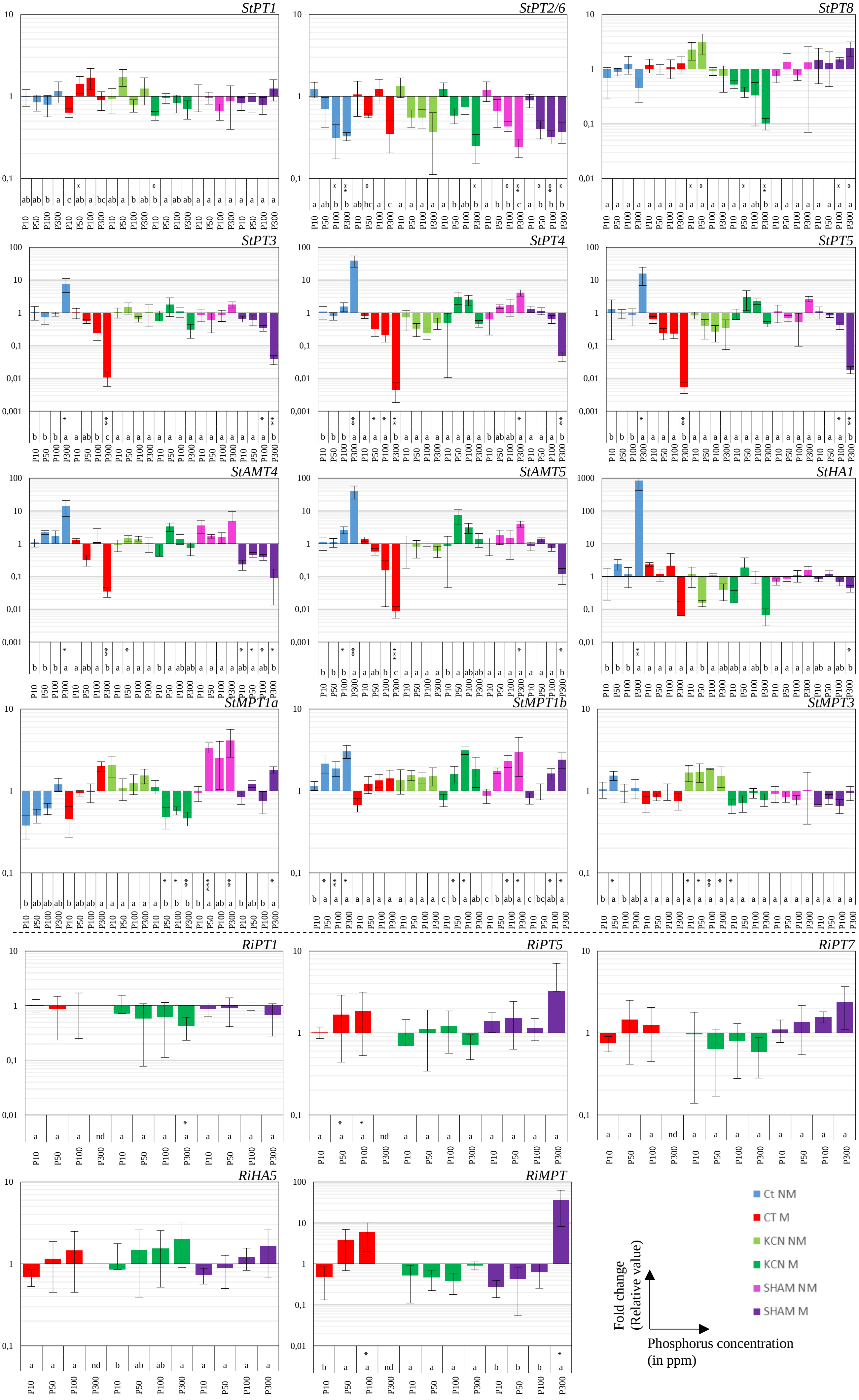

Figure S9. Effect of P concentration on the relative expression of genes involved in phosphorus, nitrogen and proton plasma membrane transporters (6 *StPT* isoforms, 2 *StAMT* isoforms, *StHA1*, 3 *RiPT* isoforms and *RiHA5*) and mitochondrial phosphorus transporters (3 *StMPT* isoforms and *RiMPT*) in potato root and in *R. irregulare*, following inoculation or not of AMF, treatment or not with two respiratory inhibitors through 5 phosphorus concentrations. Relative expressions are represented relative to P concentration (compared to 1 ppm P as reference respective to each treatment, root samples harvested after 8 WAI). Differences in the mean expression level (*n*=3), where each sample was analyzed in duplicate of target genes between treatments were examined by a one-way analysis of variance (ANOVA) after a log transformation of the normalized relative gene expression levels. Dunnett's test was conducted to identify significant differences (symbolized by stars placed above P concentrations in x-axis; NS: P>0.05, *: P<0.05, **: P<0.005, ***: P<0.0005) compared to 1 ppm P as reference respective to each treatment. Duncan's multiple range tests were performed to identify significant differences ($P < 0.05$, symbolized by letters indicated above graphs) among P concentrations (after standardization against specified control). Data analysis was performed with the SAS enterprise guide 4.1 (SAS Institute Inc., Cary, USA).

1.10 Supplementary Figure S10

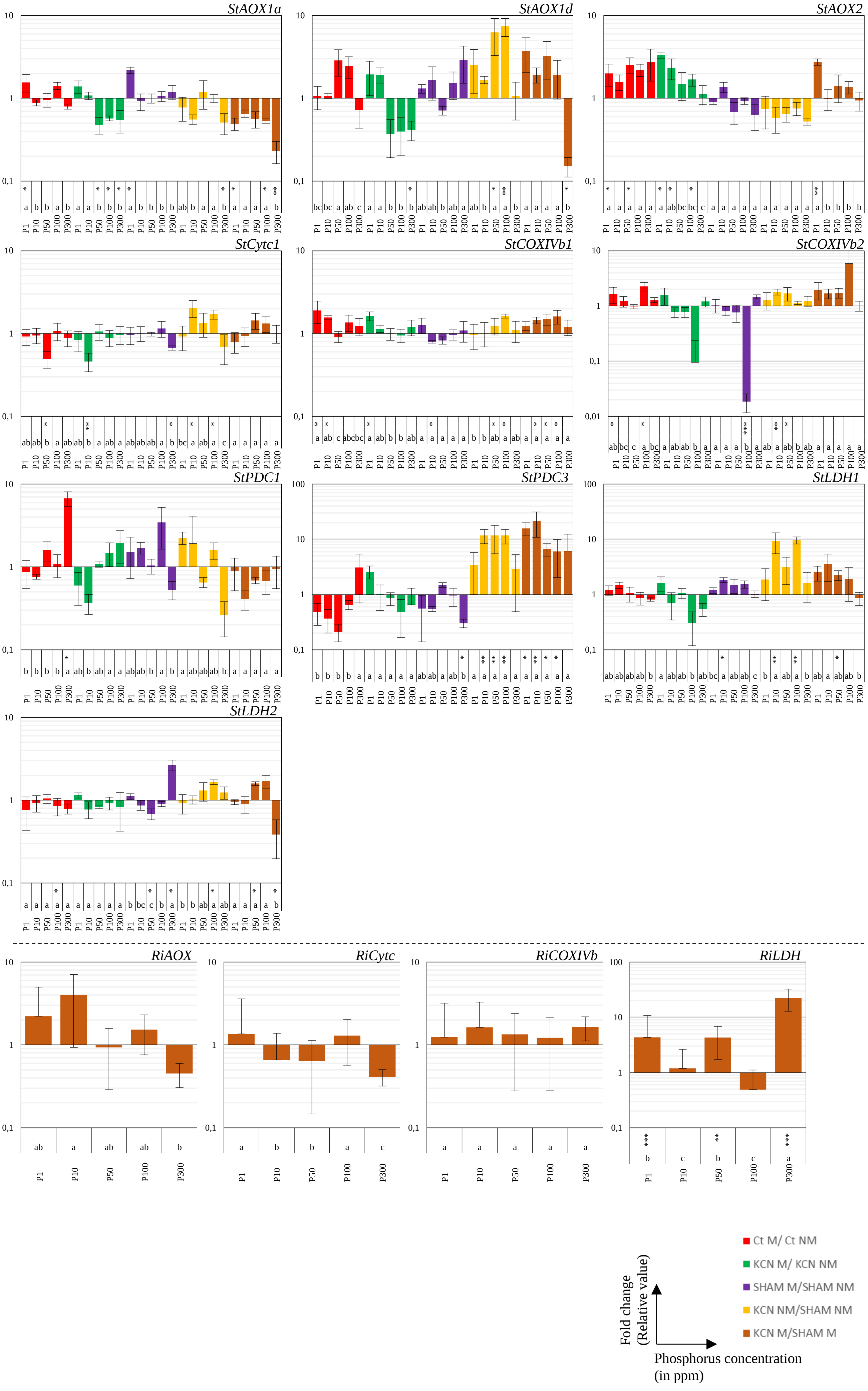

Figure S10. Effect of treatments on the relative expression of genes involved in mitochondrial respiratory chain (3 *StAOX* isoforms, *StCytc1, StCOXVb1, StCOXVb2, RiAOX, RiCytc and RiCOXIVb)* and fermentation (2 *StPDC* and 2 *StLDH* isoforms and *RiLDH*) in potato root and in *R. irregulare*, following inoculation or not of AMF, treatment or not with two respiratory inhibitors through 5 phosphorus concentrations. Relative expressions are represented relative to references specified in the legend for each P concentration (root samples harvested after 8 WAI). Differences in the mean expression level (*n*=3), where each sample was analyzed in duplicate of target genes between treatments were examined by a one-way analysis of variance (ANOVA) after a log transformation of the normalized relative gene expression levels. Dunnett's test was conducted to identify significant differences (symbolized by stars placed above P concentrations in x-axis; NS: P>0.05, *: P<0.05, **: P<0.005, ***: P<0.0005) compared to references specified in the legend for each P concentration. Duncan's multiple range tests were performed to identify significant differences ($P < 0.05$, symbolized by letters indicated above graphs) among P concentrations (after standardization against specified control). Data analysis was performed with the SAS enterprise guide 4.1 (SAS Institute Inc., Cary, USA).

1.11 Supplementary Figure S11

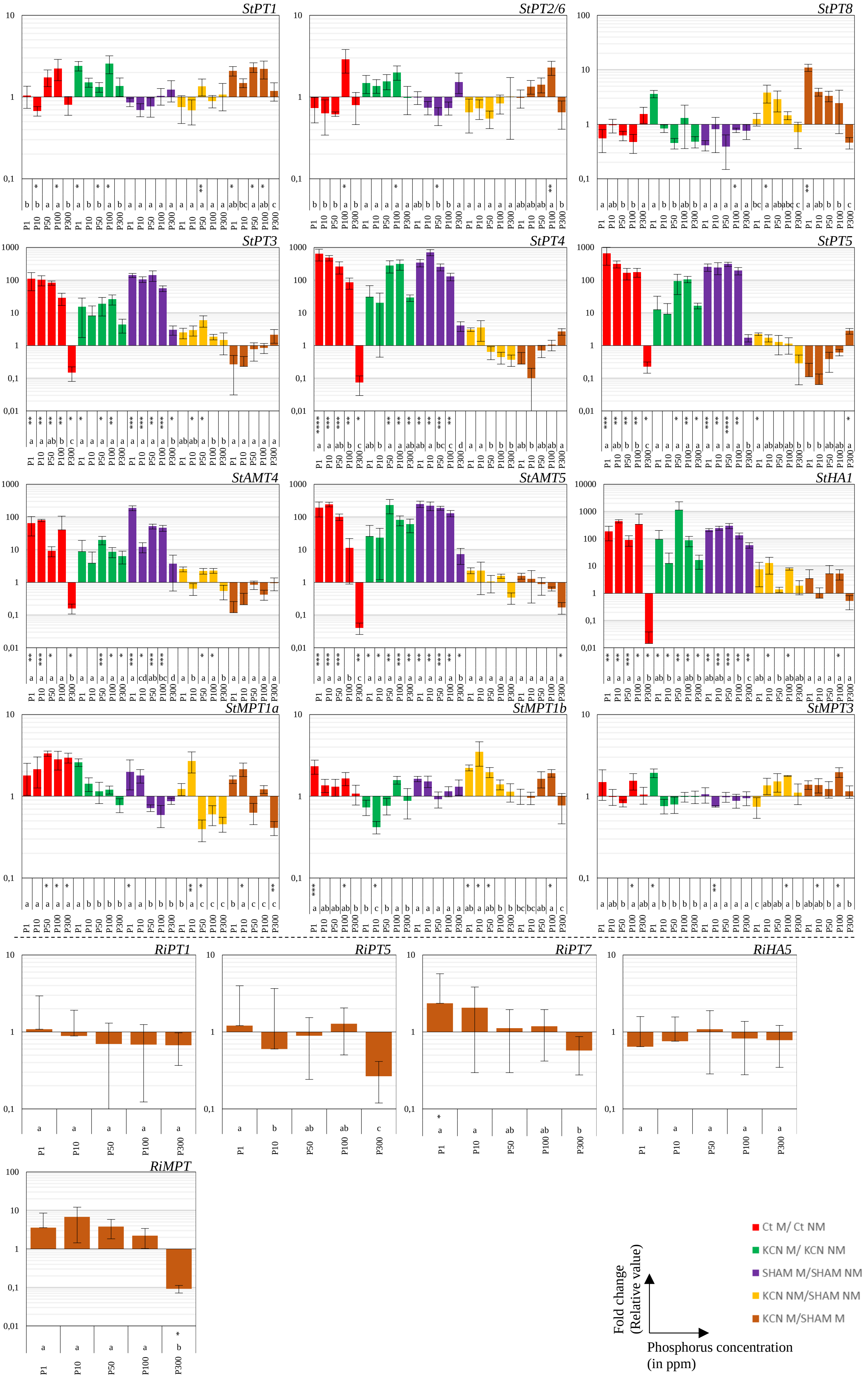

Figure S11. Effect of treatments on the relative expression of genes involved in phosphorus, nitrogen and proton plasma membrane transporters (6 *StPT* isoforms, 2 *StAMT* isoforms, *StHA1*, 3 *RiPT* isoforms and *RiHA5*) and mitochondrial phosphorus transporters (3 *StMPT* isoforms and *RiMPT*) in potato root and in *R. irregulare*, following inoculation or not of AMF, treatment or not with two respiratory inhibitors through 5 phosphorus concentrations. Relative expressions are represented relative to references specified in the legend for each P concentration (root samples harvested after 8 WAI). Differences in the mean expression level (n=3), where each sample was analyzed in duplicate of target genes between treatments were examined by a one-way analysis of variance (ANOVA) after a log transformation of the normalized relative gene expression levels. Dunnett's test was conducted to identify significant differences (symbolized by stars placed above P concentrations in x-axis; NS: P>0.05, *: P<0.05, **: P<0.005, ***: P<0.0005) compared to references specified in the legend for each P concentration. Duncan's multiple range tests were performed to identify significant differences ($P < 0.05$, symbolized by letters indicated above graphs) among P concentrations (after standardization against specified control). Data analysis was performed with the SAS enterprise guide 4.1 (SAS Institute Inc., Cary, USA).

2 Supplementary Tables

2.1 Supplementary Table S1: Primers used in this work

Name	Function	Accession	Primer sequence 5'-3'	Size (bp)	Reference for the primers
GintS4	Ribosomal protein from the small ribosomal subunit	ESA09463[a]	F: TCTTGTGAAGGTTGATGGCAAA<br>R: CGCCATTTCTTTCGATCGA		Govindarajulu *et al.*, 2005
GintEF1a	Elongation factor		F: GCTATTTTGATCATTGCCGCC<br>R: TCATTAAAACGTTCTTCCGACC	167	Fiorilli *et al.*, 2013
Giβ-tub	β-tubulin		F: AGAAAGTCTACCACGGAAAATAGTAGCT<br>R: TTCACGTAATATGATGGCTGCAT	88	Lammers *et al.*, 2001
RMR-RMF	18S rRNA of R. irregulare		F: TGTTAATAAAAATCGGTGCGTTGC<br>R: AAAACGCAAATGAT CAACCGGAC	465	González-Guerrero *et al.*, 2005
GintICL	Isocitrate lyase	BE603746.1[a]	F: TGCTACTCTTCTCACATCTAACATCGA<br>R: CAAGAGGGCGAAGGTTAGGA	83	Lammers *et al.*, 2001
Stβ-tub	β-tubulin	Z33382[a]	F: ATGTTCAGGCGCAAGGCTT<br>R: TCTGCAACCGGGTCATTCAT	101	Gallou 2009
StEF1	Elongation factor		F: ATTGGAAACGGATATGCTCCA<br>R: TCCTTACCTGAACGCCTGTCA	101	Gallou 2009
StGADPH	Glyceraldehyde 3-phosphate dehydrogenase		F: GGACATTGTCTCCAACGC<br>R: ATGAGACCCTCCACAATGC	93	Gallou 2009
StUbc	Ubiquitin		F: TGATGGTTACCCATTTGAGCC<br>R: ACTGGTCCTTCAGGATGTC	110	Gallou 2009
StAOX1a	Alernative oxidase	XM_006366820.1[a]	F: CCCTGGTTTTCGCAGTGC<br>R: GGAGAGTGGCATCCTTAGGC	219	This study
StAOX1b	Alernative oxidase	PGSC0003DMT400019707[c]	F: TCTCAATATGAGTCGTAGTGCAGC<br>R: GAGTTTTCACCCCGTGGAG	83	This study
StAOX1d	Alernative oxidase	XM_006361172[a]	F: TTGGCGCGATCTATGATGTC<br>R: AAATTGAACGATGCACCGGA	110	This study
StAOX2	Alernative oxidase	PGSC0003DMT400032705 [c]	F: CCGTTGCATATCGTCTGTGT<br>R: TTTCTCGTCCTCCTTCCTCA	199	This study
StCytC1	Cytochrome C	PGSC0003DMT400063997 [c]	F: GTAATCCTAAAGCCGGAGAAAAA<br>R: CGCAGTTGCTTCCTTGAGATAA	308	This study
StCytC2	Cytochrome C	PGSC0003DMT400002609 [c]	F: TTTGGAAGGCAGTCTGGAAC<br>R: CAGTAGCAGATTTCAGGTACGCA	202	This study
StCOX5b1	Cytochrome C oxidase	XP_006346591[a]	F: GCATCTCCGAACCCTACACCTAC<br>R: GCCAGTTGCAATGGGCATTA	199	This study
StCOX5b2	Cytochrome C oxidase	PGSC0003DMT400073483 [c]	F: CAGGTTCGGACTCTAAGCCC<br>R: GCGTTCGTGACCAGTAGCAA	195	This study
StPT1	Plasma membrane phosphate transporter	PGSC0003DMT400044369 [c]	F: ATTCAAGGGAGCATTCCCTG<br>R: TCGTTAGCTGCCTGTTTCAAGT	201	This study
StPT2/6	Plasma membrane phosphate transporter	PGSC0003DMT400034874 [c]	F: ACTTTCGCGGAAAATGCTG<br>R: TCAACTTGTAGTACCCTCCCCAT	200	This study
StPT3	Plasma membrane phosphate transporter	AJ318822[a]	F: ACTTGTTGTTAGTGGTGCATTTAA<br>R: GGAAGCAGCCTTAGTAGCATT	217	This study, Gallou 2009
StPT4	Plasma membrane phosphate transporter	PGSC0003DMT400006059 [c]	F: GACAGCTATGATACTGATGGCCG<br>R: GGCCAGAGACAGGTTTGCTAGT	155	This study
StPT5	Plasma membrane phosphate transporter	PGSC0003DMT400006063 [c]	F: GAAAGCCATGTTGTTTTTGGC<br>R: CGCCCAGAGTCAGTCCTATTACTC		This study
StPT8	Plasma membrane phosphate transporter	PGSC0003DMT400026632 [c]	F: GTCGATGGCTCTTCAAAGCC<br>R: CATGACGGTCTTAGGCTCCC	216	This study
StMPT1a	Mitochondrial phosphate transporter	PGSC0003DMT400038023 [c]	F: AGGAGTTCTCGGCTGGGTATTA<br>R: CTCCATAGTGTTATAAGCCCTGATG	163	This study
StMPT1b	Mitochondrial phosphate transporter	PGSC0003DMT400048076 [c]	F: CACATGCTTTACGACTTTGTTG<br>R: AATCTACAAGCACCCTGAACAC	102	This study
StMPT2	Mitochondrial phosphate transporter	PGSC0003DMT400069570 [c]	F: CACCTGCTTATTACGGTGCTTG<br>R: CACCAAAACCAGAGGAAATGC		This study
StMPT3	Mitochondrial phosphate transporter	PGSC0003DMT400052032 [c]	F: TACTCCCCGCAGTTCTACGC<br>R: GATGCCAAATCCAGATGATATAC	150	This study
StHA1	Plasma membrane H+-ATPase	PGSC0003DMP400060260 [c]	F: AGGAGAGCCTTCGGATATCATT<br>R: AGTGCTTCTTGCTTTCTTCTTGTC	224	This study
StAMT4	Ammonium transporter	PGSC0003DMT400049775 [c]	F: ACTGGCTTAGTTTGCATTACGCCT<br>R: AACCATGGAATGCATCCGGACATT	92	Ruzicka *et al.*, 2012

StAMT5	Ammonium transporter	PGSC0003DMT400059578 [c]	F: CTTTGCTCACCCGAGACTATGTTA<br>R: TCCCAAGAAGTTGGAGACCCATTT	128	Ruzicka *et al.*, 2012
StPDC1	Pyruvate decarboxylase	PGSC0003DMT400016038 [c]	F: GCGGCTGAGTTCTTGAACAA<br>R: ACAGAAGGTCGTGCTCACTG	207	This study
StPDC2	Pyruvate decarboxylase	PGSC0003DMT400078075 [c]	F: GACCCAAAAGGTAAGGACGG<br>R: CGTCCATTGGCAGAACAGA	162	This study
StPDC3	Pyruvate decarboxylase	PGSC0003DMT400059095 [c]	F: GTCCCAAAGTAAGAGTGGCAAA<br>R: CCTTTTTAATCAGCAATGAGTATCC	255	This study
StLDH1	Lactate dehydrogenase	PGSC0003DMT400012233 [c]	F: AGTGCCAGTCCTCAGTTTCCTT<br>R: CCTTTGCAAGAACCGAAACA	212	This study
StLDH2	Lactate dehydrogenase	PGSC0003DMT400032491 [c]	F: GAGTGTATATTGTTGATTGTGTCGAA<br>R: CAGAAAGCTAAGAACCGGAATC	258	This study
RiAOX	Alternative oxidase	KT423114.1[a]	F: AAAATGAACGTATGCACTTGATGAC<br>R: GCGTTCCCACCAGGTAGGT	62	Campos *et al.*, 2015
RiCOX5b	Cytochrome C oxidase	ESA20394[a]	F: TTGTCGGCTGTACTGGGTTC<br>R: ACCACATTCAGGGCATCTGT	95	This study
RiCytC	Cytochrome C	ESA22931[a]	F: GCTGCAAGTGCTGATGCTAA<br>R: GGAACCTCCTGCTTCGACTG	84	This study
RiPT1	Plasma membrane phosphate transporter	AF359112.1[a]	F: AACACGATGTCAACAAAGCAAC<br>R: AAGACCGATTCCATAAAAAGCA	242	Fiorilli *et al.*, 2013
RiPT3	Plasma membrane phosphate transporter	ESA01683.1[a]	F: AAAGGCGTGGAGCAATGA<br>R: CGGGAATAATACCGACACCA	171	This study
RiPT5	Plasma membrane phosphate transporter	ESA15049[a]	F: CCGCCCGTAGTGTGAATAAA<br>R: GAAGCGAATGAGGCAGTAAGAAT	139	This study
RiPT6	Plasma membrane phosphate transporter	Remain_c13721[b]	F: AACCGGAGCTTTCGCTTCA<br>R: AGCATCGATAGCAGCTCCAC	166	This study
RiPT7	Plasma membrane phosphate transporter	KU219934[a]	F: CCAGTCTCAGGATTCCCAAA<br>R: CCGATCGTGACAACACAAAG	137	This study
RiMPT	Mitochondrial phosphate transporter	Step3_c284[b]	F: GGCGAAGAAACTGCTCACA<br>R: AGAACTTCGGCACTTGCACT	68	This study
RiHA5	Plasma membrane H+-ATPase	AF420481[a]	F: AATGATGCAGCCACTCTCGT<br>R: CCAAGACGCCATTTATCAGG	74	This study
RiLDH	Lactate dehydrogenase	ERZ99847[a]	F: CGCTGTAATTGGAGCTGGTT<br>R: TTGACCAGCCTCTTGGAAAG	190	This study

a: GenBank (http://www.ncbi.nlm.nih.gov/genbank/) ; b: *Rhizophagus irregularis* DB (http://mycor.nancy.inra.fr/IMGC/GlomusGenome/search3.html) c: Potato Genomics Resource (http://solanaceae.plantbiology.msu.edu/pgsc_download.shtml)

2.2 Supplementary Table S2

Table S2. Regression coefficients after solving the overall equation fitted for predicted and observed mycorrhizal rate parameters of potato plants treated with respiratory chain inhibitors or not, inoculated with *R. irregulare* under 5 phosphorus concentrations. Larger significance of coefficients, F and P values for the regressions (degrees of freedom between brackets), coefficients of determination and percentage of the model explained by terms in ln P alone of the overall equation are also presented. Candidate models included up to the third degree of ln P, dummy variables binary coded for treatments and for inoculation and all first and second level interactions between variables. Data were subjected to transformation or not before processing, as indicated below each mycorrhizal parameter.

Variables	Treatments	Y intercept	ln P	$(\ln P)^2$	$(\ln P)^3$	P	P^2	P^3	P coefficients	F	P	R^2	Explanation by P or ln P (%)
F%	M & MSHAM	88.689	−	−	−	0	8.25×10^{-4}	0	$<10^{-5}$	26.8 (3,41)	$<10^{-5}$	0.883	63.8
	MKCN	52.067	−	−	−	0.258	8.25×10^{-4}	0					
M%	M	57.121	-6.435	-0.551	0	−	−	−	$<10^{-5}$	35.6 (3,41)	$<10^{-5}$	0.851	63.3
arcsin	MKCN & MSHAM	25.190	0	-0.551	0	−	−	−					
m%	M	56.467	-5.576	-0.573	0	−	−	−	$<10^{-4}$	35.4 (3,41)	$<10^{-5}$	0.870	68.1
arcsin	MKCN & MSHAM	28.629	0	-0.573	0	−	−	−					
A%	M	35.798	-4.019	-0.335	0	−	−	−	$<10^{-5}$	48.3 (3,41)	$<10^{-5}$	0.933	57.9
arcsin	MKCN & MSHAM	14.254	0	-0.335	0	−	−	−					
a%	M & MKCN	4.021	0	0	-0.004	−	−	−	$<10^{-5}$	36.2 (2,42)	$<10^{-5}$	0.931	31.3
ln	MSHAM	3.130	0	0	-0.004	−	−	−					
V%	M	27.113	-5.369	0	0	−	−	−	<0.011	38.5 (3,41)	$<10^{-5}$	0.837	51.4
arcsin	MKCN & MSHAM	7.322	-1.051	0	0	−	−	−					
v%	M & MSHAM	2.818	0	-0.094	0	−	−	−	$<10^{-5}$	20.4 (3,41)	$<10^{-5}$	0.850	65.5
ln (v+1)	MKCN	0.946	0	-0.007	0	−	−	−					
H %	M & MSHAM	23.776	0	0	-0.118	−	−	−	$<10^{-5}$	29.6 (3,41)	$<10^{-5}$	0.905	59.6
arcsin	MKCN	8.392	0	0.650	-0.118	−	−	−					
h %	M & MKCN	30.818	4.279	0	0	−	−	−	$<10^{-5}$	49.1 (2,42)	$<10^{-5}$	0.907	57.7
arcsin	MSHAM	46.250	4.279	0	0	−	−	−					

2.3 Supplementary Table S3

Table S3. Regression coefficients after solving the overall equation fitted for predicted and observed (means±SE) of potato *in vitro* plantlets growth parameters treated with respiratory chain inhibitors or not and inoculated or not with *R. irregulare* under 5 phosphorus concentrations. Larger significance of coefficients, F and P values for the regressions (degrees of freedom between brackets), coefficients of determination and percentage of the model explained by terms in ln P alone of the overall equation are also presented. Candidate models included up to the third degree of ln P, dummy variables binary coded for treatments and for inoculation and all first and second level interactions between variables. Data were subjected to transformation or not before processing, as indicated below each mycorrhizal parameter.

Plant growth parameter	Treatments	Y intercept	ln P	$(\ln P)^2$	$(\ln P)^3$	P coefficients	F	P	R^2	Explanation by ln P (%)
Shoot size	M	23.869	2.368	0	-0.045	<0.005	35.3 (5,84)	$<10^{-5}$	0.808	55.4
	NM	23.869	2.368	0	0					
	M KCN	19.842	2.368	0	0					
	NM KCN	19.842	2.368	0	0					
	M SHAM	28.516	2.368	-0.189	0					
	NM SHAM	28.516	2.368	0	0					
Shoot FW	M	5.600	0	0.0492	-0.057	$<10^{-5}$	69.4 (4,85)	$<10^{-5}$	0.877	73.4
	NM	5.600	0	0.0492	-0.057					
	M KCN	2.108	0	0.0492	0					
	NM KCN	2.108	0	0.0492	0					
	M SHAM	5.600	0	0.0492	-0.051					
	NM SHAM	5.600	0	0.0492	0					
Root FW	NM and M	1.355	0.387	0	-0.006	<0.008	31.7 (5,84)	$<10^{-5}$	0.842	44.5
	M KCN	0.360	0.387	0	0.021					
	NM KCN	0.360	0.387	0	0					
	M SHAM	1.355	0.387	0	-0.010					
	NM SHAM	1.355	0.387	0	0					
Yield FW	NM and M	0.343	1.270	0	-0.011	$<10{-}4$	89.8 (5,84)	$<10{-}5$	0.950	52.0
	M KCN	0.343	0.015	0	0.031					
	NM KCN	0.343	0.015	0	0					
	M SHAM	0.343	0.880	0	0					
	NM SHAM	0.343	1.270	0	0					
Total FW + yield	NM and M	7.460	0	0.537	-0.061	<0.001	58.3 (5,84)	$<10^{-5}$	0.901	69.2
	M KCN	2.534	0	0.537	0.037					
	NM KCN	2.534	0	0.537	0					
	M SHAM	7.460	0	0.537	-0.060					
	NM SHAM	7.460	0	0.537	0					

2.4 Supplementary Table S4

Table S4. Correlation table between plant and fungal genes with mycorrhizal parameters and mycorrhizal plants responses in non-treated plant groups (MSD: Mycorrhizal Shoot fresh weight Dependency; MRD: Mycorrhizal Root fresh weight Dependency; MYD: Mycorrhizal Yield fresh weight Dependency; MGD: Mycorrhizal Growth Dependency which correspond to total plant fresh weight biomass).

	F%	M%	m%	A%	a%	V%	v%	H%	h%	Spore number	a%/ h%	Arum/ Paris type	MSD	MRD	MYD	MGD
StAOX1a	0,21	0,73	0,73	0,75	0,33	0,86	0,89	-0,78	0,23	0,30	0,88 *	0,33	0,50	0,51	0,82	0,68
StAOX1d	0,29	-0,17	-0,16	-0,14	0,42	-0,06	-0,02	-0,12	-0,36	0,59	0,16	0,29	-0,45	0,41	0,39	-0,13
StAOX2	-0,83	-0,98 ***	-0,98 ***	-0,98 ***	-0,77	-0,88 *	-0,83	0,96 *	-0,86	-0,24	-0,87	-0,80	-0,40	0,11	-0,47	-0,38
StCytC1	-0,96 *	-0,57	-0,58	-0,57	-0,93 *	-0,45	-0,40	0,70	-0,61	-0,50	-0,56	-0,89 *	0,15	0,18	-0,28	0,07
StCOX5b1	-0,94 *	-0,46	-0,47	-0,45	-0,86	-0,26	-0,18	0,53	-0,69	-0,37	-0,33	-0,76	0,24	0,46	0,02	0,27
StCOX5b2	-0,71	-0,65	-0,66	-0,63	-0,66	-0,39	-0,28	0,54	-0,97 *	-0,16	-0,34	-0,85	-0,02	0,74	0,23	0,20
StPT1	0,04	-0,49	-0,49	-0,47	0,07	-0,38	-0,33	0,26	-0,58	0,28	-0,20	-0,07	-0,46	0,38	0,14	-0,21
StPT2/6	0,72	0,42	0,42	0,40	0,33	0,35	0,32	-0,39	0,46	-0,34	0,30	0,29	0,39	-0,04	0,19	0,30
StPT3	0,87	0,93 *	0,93 *	0,92 *	0,78	0,77	0,70	-0,88 *	0,94 *	0,21	0,74	0,83	0,30	-0,31	0,28	0,22
StPT4	0,81	0,98 **	0,98 **	0,98 **	0,68	0,89 *	0,83	-0,93 *	0,87	0,09	0,84	0,72	0,50	-0,12	0,44	0,45
StPT5	0,78	0,99 **	0,98 **	0,98 **	0,63	0,95 *	0,92 *	-0,96 *	0,75	0,07	0,91 *	0,64	0,60	0,10	0,62	0,60
StPT8	-0,69	-0,39	-0,40	-0,42	-0,78	-0,45	-0,46	0,64	-0,13	-0,66	-0,63	-0,68	0,17	-0,33	-0,63	-0,12
StHA1	0,71	0,22	0,22	0,19	0,41	0,00	-0,07	-0,16	0,58	-0,20	-0,03	0,42	-0,03	-0,54	-0,31	-0,23
StAMT4	0,78	0,59	0,58	0,56	0,38	0,45	0,39	-0,48	0,70	-0,37	0,36	0,39	0,46	-0,24	0,09	0,28
StAMT5	0,78	0,85	0,85	0,83	0,69	0,64	0,55	-0,75	0,99 **	0,11	0,57	0,77	0,26	-0,51	0,05	0,09
StMPT1a	-0,93 *	-0,54	-0,54	-0,52	-0,76	-0,31	-0,22	0,53	-0,81	-0,16	-0,32	-0,78	0,06	0,54	0,09	0,19
StMPT1b	-0,71	-0,78	-0,78	-0,75	-0,56	-0,55	-0,45	0,63	-0,99 **	0,05	-0,44	-0,66	-0,29	0,59	0,08	-0,06
StMPT3	0,30	0,17	0,17	0,19	0,21	0,38	0,45	-0,36	-0,29	0,13	0,49	0,07	0,24	0,79	0,79	0,53
StPDC1	-0,98 **	-0,84	-0,84	-0,83	-0,89 *	-0,70	-0,63	0,87	-0,84	-0,31	-0,73	-0,89 *	-0,17	0,23	-0,33	-0,15
StPDC3	-0,94 *	-0,49	-0,50	-0,49	-0,85	-0,38	-0,33	0,62	-0,54	-0,38	-0,48	-0,80	0,12	0,15	-0,25	0,06
StLDH1	0,43	0,14	0,15	0,17	0,80	0,07	0,04	-0,37	0,17	0,98 **	0,28	0,77	-0,69	-0,18	0,11	-0,48
StLDH2	0,01	-0,66	-0,65	-0,66	0,13	-0,77	-0,79	0,52	-0,28	0,35	-0,63	0,09	-0,89 *	-0,40	-0,57	-0,86
StLDH1/ StPDC1	0,78	0,77	0,78	0,79	0,94 *	0,70	0,65	-0,89 *	0,64	0,70	0,80	0,94 *	-0,05	-0,07	0,47	0,08
StAOX1a/ StCytc	0,60	0,85	0,85	0,87	0,70	0,91 *	0,91 *	-0,95 *	0,45	0,49	0,98 **	0,67	0,33	0,35	0,81	0,53
StAOX1d/ StCytc	0,53	0,07	0,07	0,09	0,66	0,11	0,13	-0,34	-0,08	0,69	0,33	0,54	-0,43	0,26	0,41	-0,14
StAOX2/ StCytc	-0,22	-0,82	-0,82	-0,82	-0,15	-0,81	-0,79	0,67	-0,60	0,18	-0,68	-0,23	-0,74	-0,06	-0,41	-0,64
RiAOX	-0,32	-0,41	-0,39	-0,37	0,66	-0,43	-0,44	0,20	-0,24	0,91	-0,24	0,58	-0,91	-0,26	-0,26	-0,74
RiCytc	0,28	0,26	0,25	0,21	-0,71	0,18	0,16	0,03	0,40	-0,98 *	-0,05	-0,56	0,74	-0,11	-0,12	0,45
RiCOX	-0,13	0,14	0,13	0,17	-0,22	0,43	0,53	-0,32	-0,57	-0,07	0,52	-0,40	0,47	0,99 *	0,87	0,75
RiPT1	0,49	0,53	0,52	0,49	-0,54	0,48	0,47	-0,28	0,47	-0,89	0,27	-0,41	0,91	0,08	0,16	0,69
RiPT5	-0,99 *	-0,95 *	-0,95 *	-0,93	-0,36	-0,81	-0,74	0,80	-0,89	0,19	-0,28	-0,05	-0,66	0,14	-0,26	-0,51
RiPT7	-0,77	-0,69	-0,68	-0,65	0,15	-0,50	-0,44	0,40	-0,86	0,66	-0,68	-0,50	-0,73	0,32	0,07	-0,42
RiMPT	-0,98 *	-0,90	-0,90	-0,88	-0,51	-0,72	-0,64	0,76	-0,93	0,04	-0,61	-0,65	-0,48	0,29	-0,13	-0,32
RiHA5	-0,87	-0,71	-0,71	-0,68	-0,45	-0,46	-0,36	0,52	-0,99 *	0,07	-0,32	-0,64	-0,28	0,58	0,19	-0,04
RiLDH	0,98 *	0,89	0,89	0,87	0,42	0,71	0,63	-0,73	0,95 *	-0,13	0,57	0,58	0,53	-0,30	0,10	0,35
RiAOX/ Cytc	-0,33	-0,40	-0,39	-0,37	0,66	-0,41	-0,41	0,18	-0,28	0,92	-0,21	0,57	-0,90	-0,21	-0,21	-0,71

2.5 Supplementary Table S5

Table S5. Correlation table between plant and fungal genes with mycorrhizal parameters and mycorrhizal plants responses in KCN plant groups (MSD: Mycorrhizal Shoot fresh weight Dependency; MRD: Mycorrhizal Root fresh weight Dependency; MYD: Mycorrhizal Yield fresh weight Dependency; MGD: Mycorrhizal Growth Dependency which correspond to total plant fresh weight biomass).

	F%	M%	m%	A%	a%	V%	v%	H%	h%	a%/ h%	Arum/ Paris type	MSD	MRD	MYD	MGD
StAOX1a	0,34	0,77	0,63	0,88 *	0,62	0,88 *	0,83	-0,69	0,48	0,64	0,54	-0,02	0,24	0,64	0,20
StAOX1d	-0,04	0,54	0,56	0,89 *	0,87	0,85	0,70	-0,92 *	0,13	0,90 *	0,72	0,38	0,37	0,81	0,56
StAOX2	0,20	0,32	0,15	0,45	0,53	0,57	0,40	-0,56	0,11	0,49	0,31	0,15	0,36	0,52	0,31
StCytC1	0,77	0,05	-0,49	-0,30	-0,65	0,11	0,13	0,59	0,29	-0,56	0,02	-0,53	0,45	0,01	-0,27
StCOX5b1	0,34	-0,11	-0,56	-0,04	-0,06	0,41	0,22	0,00	-0,16	0,48	0,54	0,15	0,94 *	0,62	0,46
StCOX5b2	-0,32	0,22	0,44	0,49	0,38	0,31	0,34	-0,40	-0,01	0,08	0,59	0,30	0,04	0,36	0,33
StPT1	0,59	0,67	0,28	0,71	0,28	0,95 *	0,89 *	-0,38	0,47	0,42	0,79	-0,12	0,66	0,79	0,26
StPT2/6	-0,49	0,19	0,58	0,47	0,78	0,14	0,07	-0,74	-0,08	0,64	-0,04	0,39	-0,38	0,10	0,21
StPT3	0,86	0,89 *	0,55	0,60	-0,06	0,69	0,81	-0,04	0,89 *	-0,02	0,19	-0,66	0,03	0,20	-0,40
StPT4	0,91 *	0,63	0,20	0,24	-0,31	0,44	0,54	0,23	0,75	-0,29	-0,03	-0,73	0,07	0,01	-0,49
StPT5	0,91 *	0,67	0,26	0,28	-0,30	0,45	0,56	0,22	0,79	-0,29	-0,04	-0,75	0,02	0,00	-0,52
StPT8	-0,29	0,42	0,59	0,85	0,95 *	0,71	0,57	-0,98 **	-0,01	0,97 *	0,67	0,52	0,24	0,74	0,61
StHA1	0,86	0,87	0,48	0,63	-0,01	0,77	0,85	-0,09	0,83	0,05	0,33	-0,59	0,19	0,33	-0,28
StAMT4	0,97 *	0,81	0,40	0,39	-0,39	0,52	0,71	0,29	0,93 *	-0,32	0,09	-0,85	-0,01	0,01	-0,59
StAMT5	0,97 *	0,73	0,35	0,25	-0,51	0,36	0,59	0,43	0,93 *	-0,46	-0,09	-0,93 *	-0,14	-0,16	-0,72
StMPT1a	-0,73	0,01	0,50	0,42	0,83	0,07	-0,03	-0,78	-0,30	0,73	0,09	0,61	-0,30	0,18	0,41
StMPT1b	0,43	-0,28	-0,68	-0,52	-0,48	-0,16	-0,24	0,46	-0,07	-0,48	-0,22	-0,24	0,37	-0,10	-0,13
StMPT3	-0,08	-0,12	-0,33	0,24	0,53	0,53	0,23	-0,56	-0,39	0,60	0,71	0,58	0,89 *	0,84	0,81
StPDC1	0,20	-0,51	-0,88	-0,57	-0,36	-0,15	-0,32	0,35	-0,37	-0,31	0,03	0,07	0,63	0,12	0,20
StPDC3	-0,68	0,10	0,54	0,53	0,89 *	0,21	0,10	-0,85	-0,26	0,81	0,23	0,63	-0,19	0,31	0,48
StLDH1	-0,51	0,30	0,73	0,60	0,78	0,22	0,20	-0,75	-0,01	0,69	0,11	0,38	-0,40	0,16	0,23
StLDH2	0,83	0,38	-0,15	0,12	-0,28	0,47	0,46	0,20	0,46	-0,21	0,20	-0,47	0,48	0,27	-0,17
StLDH1/ StPDC1	-0,54	0,17	0,67	0,35	0,54	-0,09	-0,03	-0,49	-0,01	0,41	-0,21	0,23	-0,67	-0,19	-0,03
StAOX1a/ StCytc	-0,44	0,37	0,74	0,71	0,87	0,39	0,33	-0,86	0,02	0,80	0,26	0,42	-0,24	0,33	0,33
StAOX1d/ StCytc	-0,41	0,36	0,62	0,79	0,97 *	0,58	0,45	-0,97 *	-0,06	0,95 *	0,54	0,55	0,09	0,62	0,57
StAOX2/ StCytc	-0,46	0,21	0,51	0,57	0,90 *	0,34	0,20	-0,88 *	-0,14	0,80	0,20	0,52	-0,10	0,37	0,43
RiAOX	-0,94	-0,50	0,03	-0,14	0,41	-0,45	-0,52	-0,32	-0,64	0,33	-0,13	0,66	-0,33	-0,14	0,36
RiCytc	0,01	-0,36	-0,55	-0,32	0,07	-0,07	-0,29	-0,06	-0,36	0,01	-0,11	0,22	0,37	0,12	0,25
RiCOX	-0,04	-0,64	-0,88	-0,59	-0,17	-0,24	-0,46	0,19	-0,55	-0,17	-0,03	0,28	0,57	0,11	0,33
RiPT1	-0,34	0,34	0,52	0,78	0,98 *	0,65	0,48	-0,99 *	-0,09	0,97 *	0,61	0,58	0,24	0,72	0,64
RiPT5	-0,61	0,07	0,44	0,51	0,94	0,26	0,10	-0,90	-0,29	0,84	0,24	0,65	-0,07	0,39	0,54
RiPT7	0,71	0,51	0,08	0,35	0,03	0,62	0,57	-0,11	0,48	0,07	0,26	-0,34	0,41	0,38	-0,06
RiMPT	-0,44	-0,24	-0,17	0,17	0,37	0,23	0,09	-0,37	-0,48	0,51	0,72	0,64	0,58	0,61	0,74
RiHA5	0,41	-0,35	-0,72	-0,63	-0,81	-0,29	-0,25	0,79	-0,04	-0,71	-0,10	-0,34	0,35	-0,20	-0,22
RiLDH	-0,08	0,47	0,49	0,83	0,68	0,80	0,71	-0,73	0,10	0,80	0,90	0,37	0,45	0,83	0,59
RiAOX/ Cytc	-0,89	-0,45	0,08	-0,13	0,35	-0,46	-0,49	-0,27	-0,57	0,28	-0,15	0,58	-0,39	-0,19	0,28

2.6 Supplementary Table S6

Table S6. Correlation table between plant and fungal genes with mycorrhizal parameters and mycorrhizal plants responses in SHAM plant groups (MSD: Mycorrhizal Shoot fresh weight Dependency; MRD: Mycorrhizal Root fresh weight Dependency; MYD: Mycorrhizal Yield fresh weight Dependency; MGD: Mycorrhizal Growth Dependency which correspond to total plant fresh weight biomass).

	**F%**	**M%**	**m%**	**A%**	**a%**	**V%**	**v%**	**H%**	**h%**	**a%/ h%**	**Arum/ Paris type**	**MSD**	**MRD**	**MYD**	**MGD**
StAOX1a	-0,79	-0,64	-0,65	-0,33	-0,44	-0,67	-0,65	0,51	-0,68	-0,36	-0,83	0,24	0,72	0,41	0,43
StAOX1d	-0,98 **	-0,66	-0,66	-0,56	-0,57	-0,45	-0,37	0,55	-0,75	-0,51	-0,85	0,14	0,23	-0,20	0,10
StAOX2	0,04	-0,15	-0,14	-0,46	-0,35	0,10	0,17	0,23	-0,16	-0,39	0,25	-0,33	-0,58	-0,79	-0,57
StCytC1	-0,58	-0,52	-0,53	-0,09	-0,26	-0,59	-0,58	0,33	-0,58	-0,13	-0,84	0,53	0,89 *	0,66	0,73
StCOX5b1	-0,57	-0,51	-0,51	-0,09	-0,26	-0,61	-0,62	0,34	-0,54	0,10	-0,50	0,42	0,88 *	0,67	0,66
StCOX5b2	-0,57	-0,01	-0,02	0,06	0,10	0,05	0,07	-0,06	-0,09	-0,14	-0,78	0,21	0,05	0,05	0,18
StPT1	-0,83	-0,46	-0,46	-0,23	-0,29	-0,39	-0,35	0,32	-0,54	-0,22	-0,83	0,29	0,44	0,19	0,36
StPT2/6	0,56	0,86	0,86	0,97 *	0,97 *	0,80	0,76	-0,96 *	0,76	0,99 **	0,18	0,63	-0,11	0,40	0,53
StPT3	0,85	0,83	0,83	0,91 *	0,88 *	0,57	0,48	-0,82	0,87	0,86	0,57	0,24	0,00	0,57	0,32
StPT4	0,93 *	0,89 *	0,90 *	0,68	0,77	0,75	0,68	-0,76	0,96 *	0,67	0,90 *	-0,17	-0,54	0,00	-0,23
StPT5	0,91 *	0,96 *	0,96 *	0,85	0,90	0,79	0,72	-0,88 *	0,99 **	0,83	0,77	0,05	-0,40	0,19	0,00
StPT8	-0,94 *	-0,68	-0,69	-0,72	-0,68	-0,40	-0,30	0,62	-0,77	-0,65	-0,71	-0,04	-0,03	-0,51	-0,16
StHA1	0,75	0,59	0,60	0,48	0,51	0,27	0,17	-0,42	0,75	0,40	0,83	-0,42	-0,16	0,28	-0,25
StAMT4	0,59	0,42	0,41	0,71	0,58	0,09	-0,01	-0,47	0,46	0,62	0,20	0,36	0,54	0,91 *	0,61
StAMT5	0,86	0,60	0,61	0,47	0,50	0,32	0,22	-0,43	0,76	0,40	0,91 *	-0,41	-0,22	0,21	-0,27
StMPT1a	-0,88	-0,55	-0,56	-0,50	-0,49	-0,49	-0,45	0,53	-0,57	-0,49	-0,62	-0,15	0,21	-0,10	-0,07
StMPT1b	-0,95 *	-0,91 *	-0,91 *	-0,76	-0,82	-0,73	-0,65	0,80	-0,97 *	-0,74	-0,86	0,08	0,43	-0,13	0,11
StMPT3	-0,39	-0,14	-0,15	0,20	0,09	-0,30	-0,33	0,02	-0,16	0,15	-0,56	0,37	0,66	0,68	0,59
StPDC1	-0,13	-0,58	-0,58	-0,57	-0,62	-0,44	-0,39	0,55	-0,58	-0,57	-0,15	-0,06	0,15	-0,24	-0,09
StPDC3	0,77	0,65	0,64	0,86	0,76	0,50	0,44	-0,75	0,60	0,83	0,26	0,61	0,21	0,63	0,63
StLDH1	0,23	0,64	0,63	0,84	0,81	0,55	0,51	-0,77	0,54	0,85	-0,11	0,65	0,14	0,55	0,64
StLDH2	-0,94 *	-0,56	-0,57	-0,42	-0,44	-0,40	-0,33	0,44	-0,67	-0,37	-0,87	0,24	0,30	-0,06	0,22
StLDH1/ StPDC1	0,31	0,66	0,65	0,84	0,81	0,49	0,43	-0,74	0,61	0,83	0,02	0,49	0,16	0,61	0,55
StAOX1a/ StCytc	-0,82	-0,66	-0,66	-0,55	-0,58	-0,69	-0,67	0,65	-0,62	-0,57	-0,58	-0,22	0,41	0,08	-0,03
StAOX1d/ StCytc	-0,97 *	-0,63	-0,64	-0,56	-0,56	-0,41	-0,32	0,53	-0,73	-0,51	-0,83	0,13	0,16	-0,26	0,06
StAOX2/ StCytc	0,31	0,22	0,22	-0,18	-0,03	0,42	0,47	-0,09	0,21	-0,11	0,53	-0,37	-0,82	-0,81	-0,65
RiAOX	-0,96 *	-0,59	-0,60	-0,48	-0,49	-0,40	-0,32	0,47	-0,70	-0,43	-0,86	0,20	0,24	-0,14	0,16
RiCytc	-0,88	-0,54	-0,54	-0,68	-0,59	-0,29	-0,20	0,55	-0,60	-0,61	-0,52	-0,23	-0,22	-0,61	-0,36
RiCOX	-0,11	-0,59	-0,59	-0,34	-0,50	-0,58	-0,57	0,48	-0,59	-0,38	-0,35	0,24	0,62	0,29	0,36
RiPT1	0,86	0,43	0,43	0,53	0,45	0,20	0,12	-0,42	0,50	0,46	0,55	0,13	0,18	0,50	0,25
RiPT5	-0,96 *	-0,61	-0,62	-0,63	-0,59	-0,41	-0,33	0,57	-0,68	-0,81	-0,74	-0,10	0,03	-0,37	-0,15
RiPT7	-0,98 *	-0,87	-0,88	-0,84	-0,85	-0,68	-0,60	0,82	-0,91	-0,58	-0,68	-0,13	0,23	-0,32	-0,13
RiMPT	-0,98 *	-0,66	-0,66	-0,56	-0,57	-0,47	-0,39	0,56	-0,75	-0,52	-0,84	0,11	0,24	-0,18	0,09
RiHA5	-0,93	-0,89	-0,89	-0,67	-0,76	-0,76	-0,70	0,75	-0,94	-0,66	-0,90	0,16	0,56	0,04	0,24
RiLDH	0,88	0,73	0,73	0,75	0,73	0,67	0,63	-0,76	0,70	0,74	0,52	0,39	-0,18	0,24	0,28
RiAOX/ Cytc	-0,80	-0,43	-0,44	-0,15	-0,22	-0,32	-0,27	0,24	-0,55	-0,12	-0,92	0,50	0,49	0,24	0,53

3 Supplementary Document

Mini review about the link between the alternative oxidase pathway and fermentation in plant and fungi

Although it is reported that AOX pathways is engaged in various conditions such as stress, dormancy or development in organisms, its role remains still unclear. It is usually assumed that AOX is a non-conserving energy pathway because it does not contribute to ATP formation (Vanlerberghe, 2013), meaning that other metabolic functions must be involved to sustain basal general metabolic processes associating specific redox status ($NAD(P)^+$/NAD(P)H cell pool) in order to cope with energy demand. Among others, this energy seems to be provided mainly by fermentation metabolism activity and several clues can be found in plants and fungi. In plants, 3 clues support this hypothesis:

-The first clue considers that pyruvate, NADH and CO_2 can promote the AOX pathway (Vanlerberghe, 2013; Umbach & Siedow, 2000, Gonzàlez-Meler *et al.*, 1996). These cell compounds can also be connected to fermentation via pyruvate and the reductive potential (NADH) that drives the conversion, while CO_2 creates anaerobic conditions (inhibiting COX, Gonzàlez-Meler *et al.*, 1996) in favor of fermentation. The alternative glycolytic pathway plays likely a role, as NAD/NADP-specific malic enzymes convert malate into pyruvate, NADH and CO_2. Links between malic enzyme activities with higher plant fermentation rates have been reported (Sakano, 2001). In potato, lactate oxidation in mitochondria can increase mitochondrial NADH and pyruvate pool, thus providing more energy than pyruvate alone, which can be suggested to engage the AOX pathway (Hiser *et al.*, 1996; Paventi *et al.*, 2007; Passarella *et al.*, 2008; Zhao *et al.*, 2011).

-The second clue reflects the metabolic frame induced by stresses, a context well-known to be associated with AOX, in which pyruvate is accumulating (Wu *et al.*, 2003, Juszczuk & Rychter, 2002) and it is reported that higher fermentation occurs during adaptation to biotic and abiotic stresses, such as flowering, anoxia, cold, salt, wounding, pathogen infection or P-deficiency (Tadege *et al.*, 1999, Moyano *et al.*, 2004, Uhde-Stone *et al.*, 2003). Under P-deficiency, significant induction of genes encoding for alcohol dehydrogenase was observed in white lupine, with enhanced glycolysis (Massonneau *et al.*, 2001) and formate dehydrogenase (Uhde-Stone *et al.*, 2003), indicating a possible role of fermentative processes. Moreover, and in reference to the first clue during P deficiency, it was shown that activities of both NAD/NADP-specific malic enzymes (Juszczuk & Rychter, 2002, Le Roux *et al.*, 2006) and AOX protein (Plaxton & Podesta, 2006) were up-regulated.

- The third clue comes from the role of fermentation when using respiratory inhibitors or genetic engineering. Kato-Noguchi (2000) reported that blocking electron transfer with SHAM and antimycin A simultaneously induced ethanol but not lactate formation. In transgenic tobacco plants treated with antimycin A, Vanlerberghe *et al.* (1995) observed ethanol accumulation. Inducible antisense-inhibition of *AOX1a* gene in *Arabidopsis*, whose expression is predominant in roots, led to an increase of 8.8 fold of ethanol concentration under aerobic condition (Potter *et al.*, 2001), but ethanol overproduction was also obtained by KCN application (Solomos & Laties, 1976).

In the fungal kingdom, yeasts that do not possess AOX (or at least Cyanide-Resistant-Respiration) are able to switch between aerobic metabolism and fermentation under anaerobic conditions while many other fungal species do not even possess the mitochondrial respiratory chain complex I (Veiga *et al.*, 2000). In extreme cases, some aquatic fungi, which possess only AOX as terminal electron chain, are obligatory-fermentative aero-tolerant anaerobe organisms (Natvig & Gleason, 1983), and a patent

describes enhancement of ethanol production by using a mutant yeasts strain, possessing AOX and exhibiting reduced cytochrome *c* (Jeffries & Shi, 2000). *R. irregulare* possess the classic genes involved in fermentation (lactate dehydrogenase, pyruvate decarboxylase, alcohol dehydrogenase, aldehyde dehydrogenase, alanine aminotransferase - Tisserant *et al.*, 2013) and is able to use at least acetate (Bago *et al.*, 2003; Trépanier *et al.*, 2005; Bücking *et al.*, 2008). It is known that ethanol or acetate can enter into the glyoxylate pathway (linked to AOX in plant - Day *et al.*, 1995) generating trehalose, glycogen and glycerol, in many fungal species (Orlandi *et al.*, 2013), which corresponds also to the main carbon sources in AMF (Bago *et al.*, 2003; Pfeffer *et al.*, 1999). Acetate was proposed to be metabolized via this pathway during spore germination in AMF (Bücking *et al.*, 2008).

4 References

Bago, B., Pfeffer, P.E., Abubaker, J., Jun, J, Allen, J.W., Brouillette, J., Douds, D.D., Lammers, P.J., Shachar–Hill, Y. (2003). Carbon export from arbuscular .mycorrhizal roots involves the translocation of carbohydrate as well as lipid. *Plant Physiology* 131, 1–11.

Bücking, H., Abubaker, J., Govindarajulu, M., Tala, M., Pfeffer, P.E., Nagahashi, G., Lammers, P., Shachar-Hill, Y. (2008). Root exudates stimulate the uptake and metabolism of organic carbon in germinating spores of *Glomus intraradices*. *New Phytologist* 180, 684–695.

Campos, C., Cardoso, H., Nogales, A., Svensson, J., Lopez-Ráez, J.A., Pozo, M.J., Nobre, T., Schneider, C., Arnholdt-Schmitt, B. (2015). Intra and Inter-Spore Variability in *Rhizophagus irregularis* AOX Gene. *PLoS ONE 10*(11), e0142339.

Day, D.A., Whelan, J., Millar, A.H., Siedow, J.N., Wiskich, J.T. (1995). Regulation of the alternative oxidase in plants and fungi. *Australian Journal of Plant Physiology* 22, 497–509.

Draper, N.R., Smith, H. (1998). Applied regression analysis, 3rd ed. New York, US: Wiley.

Fiorilli V., Lanfranco L., Bonfante P. (2013). The expression of GintPT, the phosphate transporter of *Rhizophagus irregularis*, depends on the symbiotic status and phosphate availability. *Planta* 237, 1267–1277.

Gallou, A. (2011). Impact of *Rhizophagus sp.* (syn. *Glomus sp*.) and *Trichoderma harzianum* on the potato resistance against *Rhizoctonia solani* and *Phytophthora infestans*, two major potato pathogens. PhD thesis, Université catholique de Louvain (Belgium).

González-Guerrero, M., Azcón-Aguilar, C., Mooney, M., Valderas, A., MacDiarmid, C.W., Eide,D.J., Ferrol, N. (2005). Characterization of a *Glomus intraradices* gene encoding a putative Zn transporter of the cation diffusion facilitator family. *Fungal Genetics and Biology* 42, 130-140.

Govindarajulu, M., Pfeffer, P.E., Jin, H.R., Abubaker, J., Douds, D.D., Allen, J.W., Bucking, H., Lammers, P.J., Shachar-Hill, Y. (2005) Nitrogen transfer in the arbuscular mycorrhizal symbiosis. *Nature* 435, 819–823.

Hiser, C., Kapranov, P., McIntosh, L. (1996). Genetic modification of respiratory capacity in potato. *Plant Physiology* 110, 277–86.

Jeffries, T.W., Shi, N.Q. (2000). *Disruption of the cytochrome C gene in xylose–fermenting yeast.* US Patent nr. US6071729 A.

Juszczuk, I., Rychter, A.M. (2002). Alternative oxidase in higher plants. *Acta Biochimica Polonica* 50, 1257–1271.

Kato-Noguchi, H. (2000). Evaluation of the importance of lactate for the activation of ethanolic fermentation in lettuce roots in anoxia. *Physiologia Plantarum* 109, 28–33.

Lammers, P.J., Jun, J., Abubaker, J., Arreola, R., Gopalan, A., Bago, B., Hernandez-Sebastia, C., Allen, J.W., Douds, D.D., Pfeffer, P.E., Shachar-Hill, Y. (2001). The Glyoxylate Cycle in an Arbuscular Mycorrhizal Fungus. Carbon Flux and Gene Expression. *Plant Physiology*, 127:1287-1298.

Le Roux, M.R., Ward, C.L., Botha, F.C., Valentine, A.J. (2006). Routes of pyruvate synthesis in phosphorus-deficient lupin roots and nodules. *New Phytologist* 169, 399– 408

Massonneau, A., Langlade, N., Leon, S., Smutny, J., Vogt, E., Neumann, G., Martinoia, E. (2001). Metabolic changes associated with cluster root development in white lupin (*Lupinus albus* L.): relationship between organic acid excretion, sucrose metabolism and energy status. *Planta* 213, 534 – 542.

Moyano, E., Encinas-Villarejo, S., Lopez-Raez, J.A., Redondo-Nevado, J., Blanco-Portales, R., Bellido, M.L., Sanz, C., Caballero, J.L. and Munoz-Blanco, J. (2004). Comparative study between two strawberry pyruvate decarboxylase genes along fruit development and ripening, post-harvest and stress conditions. *Plant Sci*. 166, 835–845.

Natvig, D.O., Gleason, F.H. (1983). Oxygen uptake by obligately–fermentative aquatic fungi: Absence of a cyanide–sensitive component. *Archives of Microbiology* 134, 5–8

Orlandi, I., Ronzulli, R., Casatta, N., Vai, M. (2013). Ethanol and Acetate Acting as Carbon/Energy Sources Negatively Affect Yeast Chronological Aging. *Oxidative Medicine and Cellular Longevity* vol. 2013, Article ID 802870, doi:10.1155/2013/802870

Passarella, S., De Bari, L., Valenti, D., Pizzuto, R., Paventi, G., Atlante, A. (2008). Mitochondria and L–lactate metabolism. *FEBS Letters* 582, 3569–3576.

Paventi, G., Pizzuto, R., Chieppa, G., Passarella, S. (2007). L–lactate metabolism in potato tuber mitochondria. *FEBS Journal* 274, 1459–1469.

Pfeffer, P.E., Douds, D.D., Bécard, G., Shachar–Hill, Y. (1999). Carbon uptake and the metabolism and transport of lipids in an arbuscular mycorrhiza. *Plant Physiology* 120, 587–598.

Plaxton, W.C., Podestá, F.E. (2006). The Functional Organization and Control of Plant Respiration. Critical Reviews in Plant Sciences 25:159–198.

Potter, F.T., Wiskich, J.T., Dry, I.B. (2001). The production of an inducible antisense alternative oxidase (Aox1a) plant. *Planta* 212, 215–221.

Ruzicka, D.R., Hausmann, N.T., Barrios-Masias, F.H., Jackson, L.E., Schachtman, D.P. (2012). Transcriptomic and metabolic responses of mycorrhizal roots to nitrogen patches under field conditions. *Plant Soil* 350, 145–162.

Solomos, T., Laties, G.G. (1976). Effects of cyanide and ethylene on the respiration of cyanide–sensitive and cyanide–resistant plant tissues. *Plant Physiology* 58, 47–50.

Tamura, K., Stecher, G., Peterson, D., Filipski, A., Kumar, S. (2013). MEGA6: Molecular Evolutionary Genetics Analysis Version 6.0. *Mol Biol Evol.* 30, 2725–2729.

Uhde-Stone, C., Gilbert, G., Johnson, J. M.-F., Litjens, R., Zinn, K. E., Temple, S. J., Vance, C.P., Allan, D. L. (2003). Acclimation of white lupin to phosphorus deficiency involves enhanced expression of genes related to organic acid metabolism. *Plant and Soil 248*, 99–116.

Umbach, A.L., Siedow, J.N. (2000). The cyanide–resistant alternative oxidases from the fungi *Pichia stipitis* and *Neurospora crassa* are monomeric and lack regulatory features of the plant enzyme. *Archives of Biochemistry and Biophysics* 378, 234–245.

Gonzàlez-Meler, M.A., Ribas-Carbó, M., Siedow, J.N., Drake, B.G. (1996). Direct inhibition of Plant Mitochondrial Respiration by Elevated CO_2. *Plant Physiol.* 112, 1349-1355.

Sakano, K. (2001). Metabolic regulation of pH in plant cells: Role of cytoplasmic pH in defense reaction and secondary metabolism. *International Review of Cytology* 206, 1–44.

Tadege, M., Dupuis, I.I., Kuhlemeier, C. (1999). Ethanolic fermentation: new functions for an old pathway. *Trends in Plant Science* 4, 320–325.

Tisserant, E., Malbreil, M., Kuo, A., Kohler, A., Symeonidi, A., Balestrini, R., Charron, P., Duensing, N., Frei, N., Gianinazzi–Pearson, V., Gilbert, L.B., Handa, Y., Herr, J.R., Hijri, M., Koul, R., Kawaguchi, M., Krajinski, F., Lammers, P.J., Masclaux, F.G., Murat, C., Morin, E., Ndikumana, S., Pagni, M., Petitpierre, D., Requena, N., Rosikiewicz, P., Riley, R., Saito, K., San Clemente, H., Shapiro, H., van Tuinen, D., Bécard, G., Bonfante, P., Paszkowski, U., Shachar–Hill, Y.Y., Tuskan, G.A., Young, J.P.W., Sanders, I.R., Henrissat, B., Rensing, S.A., Grigoriev, I.V., Corradi, N., Roux, C., Martin, F. (2013). Genome of an arbuscular mycorrhizal fungus provides insight into the oldest plant symbiosis. *Proceedings of the National Academy of Sciences* 110, 20117–20122.

Trépanier, M., Bécard, G., Moutoglis, P., Willemot, C., Gagné, S., Avis, T.J., Rioux, J.A. (2005). Dependence of arbuscular–mycorrhizal fungi on their plant host for palmitic acid synthesis. *Applied and Environmental Microbiology* 71, 5341–5347.

Ury, H.K. (1976). A comparison of four procedures for multiple comparisons among means (pairwise contrasts) for arbitrary sample sizes. *Technometrics* 18, 89–97.

Vanlerberghe, G.C., Day, D.A., Wiskich, J.T., Vanlerberghe, A.E., McIntosh, L. (1995). Alternative Oxidase Activity in Tobacco Leaf Mitochondria – Dependence on Tricarboxylic Acid Cycle–Mediated Redox Regulation and Pyruvate Activation. *Plant Physiology* 109, 353–361.

Vanlerberghe, G.C. (2013). Alternative Oxidase: A Mitochondrial respiratory pathway to maintain metabolic and signaling homeostasis during abiotic and biotic stress in plants. *International Journal of Molecular Sciences* 14, 6805–6847.

Veiga, A., Arrabaca, J.D., Loureiro–Dias, M.C. (2000). Cyanide–resistant respiration is frequent, but confined to yeasts incapable of aerobic fermentation. *FEMS Microbiology Letters* 190, 93–97.

Wu, P., Ma, L., Hou, X., Wang, M., Wu, Y., Liu, F., Deng, X.W. (2003). Phosphate starvation triggers distinct alterations of genome expression in *Arabidopsis* roots and leaves. *Plant Physiology* 132, 1260–1271.

Zhao, Y., Jin, J., Hu, Q., Zhou, H–M., Yi, J., Yu, Z., Xu, L., Wang, X., Yang, Y., Loscalzo, J. (2011). Genetically encoded fluorescent sensors for intracellular NADH detection. *Cell Metabolism* 14, 555–566
